# The return of metabolism: biochemistry and physiology of glycolysis

**DOI:** 10.1111/brv.70104

**Published:** 2025-11-30

**Authors:** Nana‐Maria Grüning, Federica Agostini, Camila Caldana, Johannes Hartl, Matthias Heinemann, Markus A. Keller, Jan Lukas Krüsemann, Costanza Lamperti, Carole L. Linster, Steffen N. Lindner, Julia Muenzner, Jens Nielsen, Zoran Nikoloski, Bettina Siebers, Jacky L. Snoep, Hezi Tenenboim, Bas Teusink, Spencer J. Williams, Mirjam M. C. Wamelink, Markus Ralser

**Affiliations:** ^1^ Charité – Universitätsmedizin Berlin, Institute of Biochemistry Charitéplatz 1 Berlin 10117 Germany; ^2^ Max Planck Institute of Molecular Plant Physiology Am Mühlenberg 1 Potsdam‐Golm 14476 Germany; ^3^ Berlin Institute of Health at Charité – Universitätsmedizin Berlin Charitéplatz 1 Berlin 10117 Germany; ^4^ University of Groningen, Groningen Biomolecular Sciences and Biotechnology Institute Nijenborgh 7 Groningen 9747 AG The Netherlands; ^5^ Medical University of Innsbruck, Institute of Human Genetics Peter‐Mayr‐Str. 1 Innsbruck 6020 Austria; ^6^ Max Planck Institute for Terrestrial Microbiology Department of Biochemistry and Synthetic Metabolism Karl‐von‐Frisch‐Str. 10 Marburg 35043 Germany; ^7^ Division of Medical Genetics and Neurogenetics Fondazione IRCCS Neurological Institute ‘C. Besta’ Via Temolo 4 Milano 20126 Italy; ^8^ Luxembourg Centre for Systems Biomedicine (LCSB) University of Luxembourg 7 avenue des Hauts‐Fourneaux Esch‐sur‐Alzette L‐4362 Luxembourg; ^9^ BioInnovation Institute Ole Maaløes Vej 3 Copenhagen DK2200 Denmark; ^10^ Chalmers University of Technology Department of Life Sciences Chalmersgatan 4 Göteborg 412 96 Sweden; ^11^ University of Potsdam, Institute of Biochemistry and Biology Bioinformatics Department Karl‐Liebknecht‐Str. 24‐25 Potsdam 14476 Germany; ^12^ Molecular Enzyme Technology and Biochemistry (MEB), Environmental Microbiology and Biotechnology (EMB), Centre for Water and Environmental Research (CWE), Department of Chemistry University of Duisburg‐Essen Universitätsstrasse 5 Essen 45141 Germany; ^13^ Stellenbosch University Department of Biochemistry P.O. Box X1 Stellenbosch 7599 South Africa; ^14^ Vrije Universiteit Amsterdam, Systems Biology Lab AIMMS/A‐LIFE De Boelelaan 1085 HV Amsterdam 1081 The Netherlands; ^15^ University of Melbourne, School of Chemistry and Bio21 Molecular Science and Biotechnology Institute 30 Flemington Rd Parkville 3010 Victoria Australia; ^16^ Amsterdam University Medical Center (UMC), location Academic Medical Center (AMC) Laboratory Genetic Metabolic Diseases Meibergdreef 9 Amsterdam 1105 AZ The Netherlands; ^17^ Max Planck Institute for Molecular Genetics Ihnestrasse 73 Berlin 14195 Germany; ^18^ Department of Human Genetics, Nuffield Department of Medicine Roosevelt Dr, Headington Oxford OX3 7BN UK

**Keywords:** energy metabolism, glycolysis, biochemical pathway, Warburg effect, Pasteur effect, Crabtree effect, thermodynamics, metabolic regulation, computational modelling, metabolic diseases

## Abstract

Glycolysis is a fundamental metabolic pathway central to the bioenergetics and physiology of virtually all living organisms. In this comprehensive review, we explore the intricate biochemical principles and evolutionary origins of glycolytic pathways, from the classical Embden–Meyerhof–Parnas (EMP) pathway in humans to various prokaryotic and alternative glycolytic routes. By examining glycolysis across the tree of life, we explore its presence and adaptation in prokaryotes, archaea, bacteria, animals and plants, and the extension of glycolysis into sulfosugar metabolism. Further, we discuss the role of unwanted side reactions, thermodynamic principles, and metabolic control principles that underpin glycolysis and the broader metabolic network, and summarise advanced methods for quantifying glycolytic activity, including new analytical methods, alongside kinetic, constraint‐based, and machine‐learning based modelling. With a focus on the Pasteur, Crabtree, and Warburg effects, this review further discusses the roles of glycolysis in health and disease, highlighting its impact on global metabolic operations, inborn errors, and various pathologies as well as its role in biotechnology and metabolic engineering.

## I. INTRODUCTION

Glucose is present in all organisms, and can be catabolized to provide energy, electrons for reducing power, and metabolic intermediates for biosynthesis. Metabolic reactions that degrade glucose to release its chemical energy and fragments are ubiquitous and are found in all three domains of life: Eukarya, Bacteria, and Archaea. While the term ‘glycolysis’ itself describes the metabolic lysis (Old Greek: *λύσις*, lysis, ‘degradation’) of glycose (an older term for glucose, Old Greek: *γλυκύς*, *glykys*, ‘sweet’), the enzymatic sequences that convert monosaccharides such as glucose into the key intermediate pyruvate are commonly named ‘glycolysis’, which collectively refers to several related metabolic pathways in this context (Flamholz *et al*., 2013; Givan, 2007; Dandekar *et al*., 1999). Research on glycolysis remains highly topical despite it being one of the oldest studied pathways of biochemistry, molecular biology and biotechnology.

Prehistoric humans first became intrigued with the fate of glucose when fruit juices were transformed into alcoholic beverages. In fact, the beginnings of scientific glycolysis research were tightly intertwined with the wine and brewing industries in the 19th century, and established yeast as a model organism for glycolysis research (Grüning & Ralser, 2021; Raihofer *et al*., 2022; Barnett, 2000). The journey to complete elucidation of the first glycolytic pathway map consisted of innumerable small experiments alongside major breakthroughs, exemplified by the awarding of several Nobel Prizes. The Embden–Meyerhof–Parnas (EMP) pathway, the formal name of the most common pathway of glycolysis, memorialises three of its discoverers: Otto Meyerhof, who won the Nobel Prize in 1922; Gustav Embden, who was nominated 12 times; and Jakub Parnas. The results of this research journey revealed the metabolic conversion of glucose and has had major impacts on our understanding of general questions of metabolism – e.g. the existence and relevance of intracellular enzymes, metabolic intermediates, cofactors, cyclic energy transformation, allostery and metabolic regulation (Grüning & Ralser, 2021).

Glycolysis occurs in all domains of life in various forms, and its presence extends to the most extreme niches that are populated by (hyper)thermophiles and halophiles. EMP‐pathway reactions can be exchanged and/or supplemented by alternative, variant or additional enzymatic steps to form variant glycolytic pathways, e.g. the Entner–Doudoroff (ED) pathway in bacteria and some eukaryotes, and variants thereof in archaea (Entner & Doudoroff, 1952; Conway, 1992; Chen *et al*., 2016; Ahmed *et al*., 2005). Many reactions are reversible and can therefore be employed for the biosynthesis of glucose in gluconeogenesis, a pathway that also operates in all domains of life (Exton, 1972).

## II. BIOCHEMICAL PRINCIPLES AND EVOLUTIONARY ORIGINS OF GLYCOLYSIS

### (1) Biochemistry of glycolytic pathways

#### (a) The EMP pathway

**Fig. 1:**
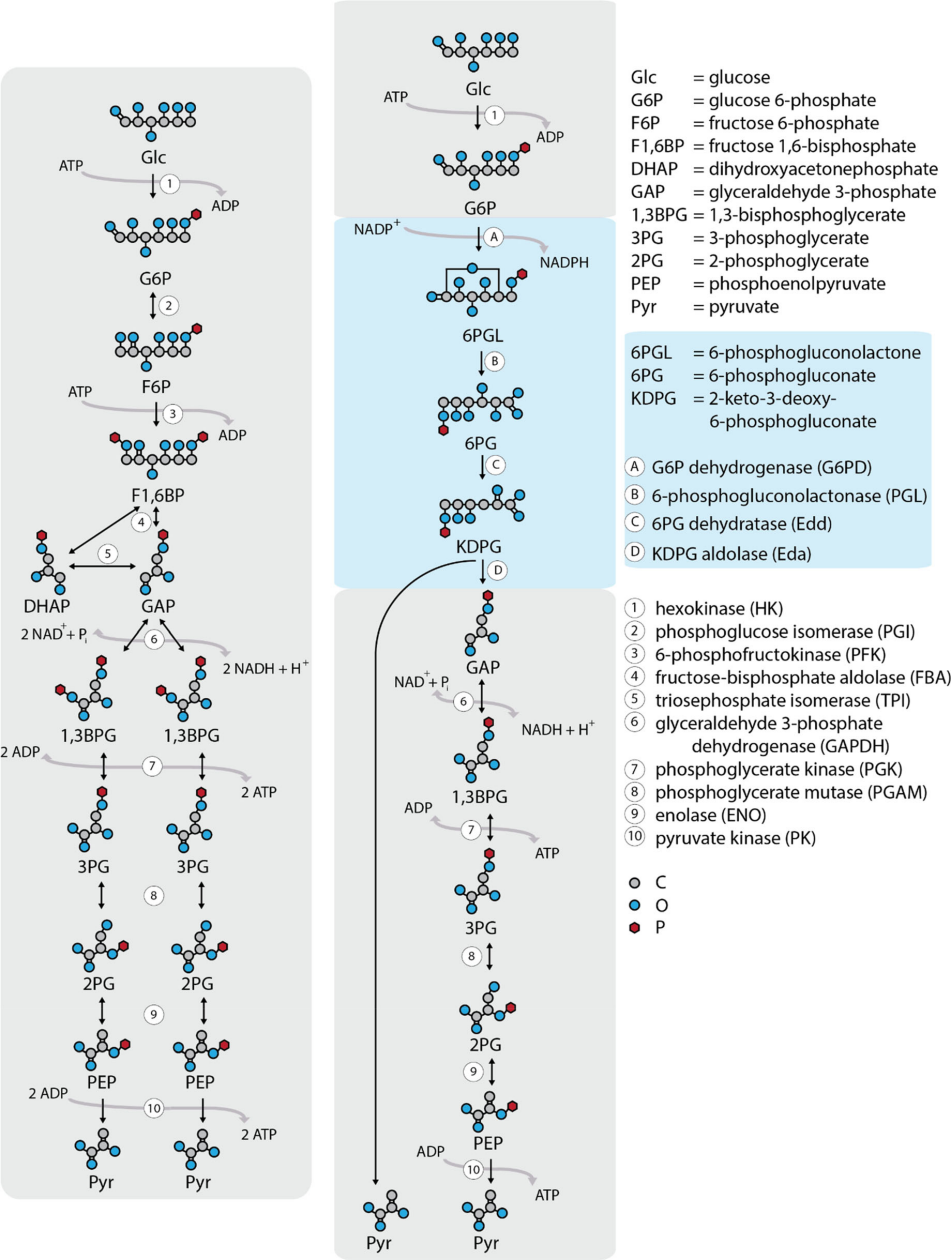
The Embden–Meyerhof–Parnas (EMP, left) and Entner–Doudoroff (ED, right) pathways. The EMP pathway involves ten enzymes and oxidises one molecule of glucose into two molecules of pyruvate, thus yielding net two molecules of adenosine triphosphate (ATP) and two molecules of reduced nicotinamide adenine dinucleotide (NADH). The classical ED pathway in bacteria replaces four EMP enzymes (phosphoglucose isomerase, PGI; 6‐phosphofructokinase, PFK; fructose‐bisphosphate aldolase, FBA; and triosephosphate isomerase, TPI) with alternative enzymes (glucose‐6‐phosphate dehydrogenase, G6PD or Zwf; 6‐phosphogluconolactonase, PGL; 6‐phosphogluconate dehydratase, Edd; and 2‐keto‐3‐deoxy‐6‐phosphogluconate aldolase, Eda). The ED pathway oxidises one molecule of glucose to two molecules of pyruvate, yielding one molecule each of ATP, NADH and NADPH.

The EMP pathway forms the backbone of cellular metabolism in many multicellular organisms, and is also present in unicellular organisms. Ten enzymatic steps, all of which occur in the cytosol in Eukaryotes, convert one molecule of glucose (C_6_H_12_O_6_) into two molecules of pyruvate (CH_3_COCOO^−^) (Table 1, Fig. 1). The net equation for glycolysis through the EMP pathway is:

$$\text{glucose}+2\;\mathrm{ADP}+{\text{2P}}_{i}+2\;{\mathrm{NAD}}^{+}\to 2\;\text{pyruvate}+2\;\mathrm{ATP}+2\;\text{NADH}+2\;H^{+}+2\;H_{2}O$$

**Table 1:** Hexose phosphorylation overview for the three domains of life.

Domain	Enzyme	Reaction	Example references
Eukarya	ATP‐HK	ATP + hexose → ADP + hexose 6P	Meyerhof (1927); Euler & Adler (1935); Colowick *et al*. (1941)
	ATP‐GK	ATP + glucose → ADP + glucose 6P	Larion & Miller (2012)
Bacteria	ATP‐GK	ATP + glucose → ADP + glucose 6P	Lunin *et al*. (2004)
	PP_i_‐GK	PolyP_n_ + glucose → PolyP_n‐1_ + glucose 6P	Tanaka *et al*. (2003)
	poly(P)/ATP‐GK	poly(P)/ATP + glucose → poly(P)‐1/ADP + glucose 6P	Mukai *et al*. (2004)
	GTP, UTP, CTP, PP_i_‐GK	GTP, UTP, CTP, polyphosphate‐dependent GK	Szymona & Widomski (1974)
	ROK‐HK	ATP + hexose → ADP + hexose 6P	Nakamura *et al*. (2012)
	PTS	PEP‐group translocation system	Kundig *et al*. (1964)
Archaea	ADP‐GK	ADP + glucose → AMP + glucose 6P	Kengen *et al*. (1995)
	ATP‐HK	ATP + hexose → ADP + hexose 6P	Nishimasu *et al*. (2007)
	CDP‐HK	CDP + hexose → CMP + hexose 6P	Guixé & Merino (2009)
	ADP‐GK/PFK	ADP‐dependent GK/PFK	Kengen *et al*. (1995)
	ATP‐GK	ATP + glucose → ADP + glucose 6P	Hansen *et al*. (2002b)
	ROK‐HK	ATP + hexose → ADP + hexose 6P	Dörr *et al*. (2003)
	PTS	PEP‐group translocation system	Pickl *et al*. (2012) in haloarchaea

The EMP pathway results in the release of chemical potential energy in an oxygen‐independent manner, which is stored in two molecules of adenosine triphosphate (ATP) and provides electrons stored in two molecules of reduced nicotinamide adenine dinucleotide (NADH). Under aerobic conditions, the NADH generated can serve as an electron carrier to either transfer electrons *via* the respiratory chain to generate ATP or to drive redox reactions. Under anaerobic conditions, the electrons stored in NADH need to be transferred to fermentation products (i.e. lactate) to prevent overaccumulation of NADH and to ensure constant flux of glycolysis to generate ATP. Furthermore, the intermediates of the EMP pathway can also be appropriated to supply the cell's biosynthetic machinery, stress response, and ATP production, and they provide allosteric control and serve as signalling molecules (Kierans & Taylor, 2024).

The overall reaction sequence can be divided into upper and lower glycolysis. Upper glycolysis is traditionally often subdivided into the preparatory (or investment) phase and the splitting phase. Lower glycolysis is also known as the payoff phase as it generates ATP and NADH (Akram, 2013; Berg, Tymoczko & Stryer, 2002).

For the reverse metabolic process – glucose biosynthesis through gluconeogenesis – seven of the ten enzymatic EMP pathway steps operate in the opposite direction, while the three ATP‐dependent enzymes are most commonly circumvented by other enzymes: pyruvate carboxylase and phosphoenolpyruvate (PEP) carboxykinase – or PEP synthetase in Bacteria and Archaea – for pyruvate kinase (PK); fructose‐1,6‐bisphosphatase (FBPase) for phosphofructokinase (PFK); and glucose 6‐phosphatase for hexokinase (HK) (Melkonian, Asuka & Schury, 2023).

Glycolytic pathways in bacteria and most archaea proceed through the same intermediates as in the classical EMP. However, in contrast to bacteria and eukaryotes, the enzymes involved in the metabolic conversions in archaea are often members of different enzyme families and do not share any sequence similarities with their respective eukaryotic and bacterial counterparts. Thus, so far, only modifications of the classical pathways (often with novel regulatory properties) have been identified in archaea (for a detailed review, see Bräsen *et al*., 2014).

##### (i) Glucose uptake and hexose phosphorylation

Glucose typically enters either by facilitated diffusion or through bidirectional transporters. In human cells, these are encoded by the glucose transporter (*GLUT*) or solute carrier 2A (*SLC2*) gene families, or *via* the sodium‐coupled glucose cotransporters of the *SGLT* or *SLC5* gene families (Wright, Hirayama & Loo, 2007). In general, species can have huge variability in these transporters and adjust their expression depending on cell intrinsic and environmental factors. For instance, in budding yeast, more than 20 different hexose transporter‐related genes have been identified thus far (Boles & Hollenberg, 1997), however only seven of them (HXT1–7) transport glucose. Glucose uptake is below the detection level in a *hxt1‐7* null strain of *Saccharomyces cerevisiae* (Reifenberger, Boles & Ciriacy, 1997).

The preparatory phase of the EMP pathway begins with attachment of a charged phosphate group to glucose to form glucose 6‐phosphate (G6P), thereby trapping the glucose inside the negatively charged cell membrane and helping to ensure that flux through the cell membrane favours import. In mammalian cells, this first reaction in glycolysis is catalysed by different ATP‐dependent hexokinase isozymes named hexokinases (HKs) or glucokinase (GK, which mainly functions in liver and pancreas) (Larion & Miller, 2012). Mammalian GK phosphorylates glucose with high specificity while other hexokinase isoforms are less specific and phosphorylate different hexoses (e.g. fructose, mannose and galactose). Various isomerases, mutases and epimerases interconvert non‐glucose/fructose sugars to produce either G6P or fructose 6‐phosphate (F6P), allowing them to enter glycolysis. Generally, HKs are allosterically or competitively inhibited by G6P and ATP (Berg *et al*., 2002), while in liver and pancreas GK can still function at high G6P concentrations (Matschinsky & Wilson, 2019).

In contrast to eukaryotic hexokinases, which show substrate promiscuity with hexoses other than glucose, the typical bacterial ATP‐dependent glucokinases are highly specific for glucose. They usually utilise only ATP as cofactor; however, in some bacteria, polyphosphate‐dependent glucokinases have been identified that may be either strictly polyphosphate‐utilising (Tanaka *et al*., 2003) or dual polyphosphate/ATP‐dependent enzymes (Table 1) (Romero‐Rodríguez *et al*., 2015). Glucokinase from *Mycobacterium tuberculosis* can use GTP, UTP, CTP and even polyphosphate as phosphoryl donor (Szymona & Widomski, 1974).

Furthermore, a fundamentally different mechanism called PEP group translocation (also called the phosphotransferase system, PTS) is used in many bacteria that couple glucose transport and phosphorylation with the transfer of phosphate from PEP to produce pyruvate (Deutscher, Francke & Postma, 2006; Kundig, Ghosh & Roseman, 1964). PEP group translocation involves both membrane‐bound and cytoplasmic enzymes.

In the domain of Archaea, genes encoding for proteins of the PTS have been identified and their transcription and encoded proteins characterised in some Haloarchaea for fructose uptake and degradation *via* glycolysis (Pickl, Johnsen & Schönheit, 2012; Cai *et al*., 2014), but mainly ADP‐dependent glucokinase and ATP‐dependent hexokinase activity has been described (Bräsen *et al*., 2014; Nishimasu *et al*., 2006). Alternatively, glucose phosphorylation may be accomplished by ADP‐dependent kinases, which may also use cytidine diphosphate (Guixé & Merino, 2009; Bräsen *et al*., 2014; Kengen *et al*., 1995). A bifunctional ADP‐dependent glucokinase/phosphofructokinase has been identified in glycogen‐forming mesophilic and hyperthermophilic methanogenic archaea such as *Methanocaldococcus jannaschii* (formerly *Methanococcus jannaschii*) (Verhees *et al*., 2001). Additionally, members of the ROK (repressor, open reading frame, kinase) family, which possess ATPase‐like domains along with an ATP‐dependent glucokinase domain occur in some bacteria and archaea (Titgemeyer *et al*., 1994; Romero‐Rodríguez *et al*., 2015; Dörr *et al*., 2003).

##### (ii) Phosphoglucose isomerase

In the second step of the EMP pathway, to prepare glucose for splitting, phosphoglucose isomerase (PGI; also called glucose 6‐phosphate isomerase) interconverts G6P and F6P by opening the glucose ring and isomerizing the aldehyde to a ketone *via* an enediol intermediate (Schray *et al*., 1973). The direction of flux is determined by the concentrations of G6P and F6P.

While many eukaryotic and bacterial PGI enzymes are considered highly specific for the interconversion of G6P and F6P, many archaeal enzymes can also catalyse the interconversion of mannose 6‐phosphate (M6P), resulting in a pool of M6P, G6P and F6P (Swan *et al*., 2004) (Table 2). M6P from this process may directly support the synthesis of GDP‐mannose (*via* mannose 1‐phosphate), which is a substrate for mannosyltransferases. These PGI enzymes are metal‐independent and operate through an enediolate intermediate with assistance from catalytic amino acids. An alternative enzyme class is the bacterial and archaeal cupin‐type PGIs (cPGI), which are strictly dependent on a divalent transition metal (e.g. Fe^2+^ or Ni^2+^) and are specific for G6P and F6P interconversion (Hansen *et al*., 2005).

**Table 2:** Phosphoglucose isomerase in the three domains of life.

Domain	Enzyme	Reaction	Example references
Eukarya	PGI	G6P ↔ F6P	Lohmann (1933)
Bacteria	PGI	G6P ↔ F6P	Schreyer (1980)
	cPGI	G6P ↔ F6P	Hansen *et al*. (2005)
Archaea	PGI	G6P ↔ F6P	Berrisford *et al*. (2003)
	PGI/PMI	G6P/M6P ↔ F6P	Swan *et al*. (2004)
	cPGI	G6P ↔ F6P	Hansen *et al*. (2005)

##### (iii) 6‐phosphofructokinase

The third enzyme of glycolysis, 6‐phosphofructokinase (PFK), uses ATP as a co‐substrate and generates fructose 1,6‐bisphosphate (F1,6BP) in Eukarya including humans. Since both G6P and F6P can be diverted into other metabolic pathways branching off from glycolysis [e.g. the pentose phosphate pathway (PPP), hexosamine and glycogen synthesis], and because of the large free energy release accompanying consumption of ATP, this reaction is unidirectional under glycolytic conditions and is considered the first committed step of glycolysis (Weber, 1977). Under gluconeogenesis, the reverse reaction is catalysed by FBPase and forms P_i_ (Gomori, 1943).

PFK is a key controlling step for the glycolytic flux, and it is subject to complex regulation. PFK is controlled by numerous activators and inhibitors from glycolysis and other pathways, collectively allowing the enzyme to sense and respond to cellular metabolic status, and thus integrating the activity of glycolysis with the needs of other pathways. Fructose 2,6‐bisphosphate (F2,6BP) is PFK's most potent activator in mammalian cells, and is synthesised from F6P and ATP by a bifunctional enzyme containing PFK‐2 and FBPase‐2 (Van Schaftingen, Hue & Hers, 1980; Pilkis *et al*., 1981). ATP, although being the PFK co‐substrate, is an inhibitor of PFK at high concentrations (Gaebler, 1956). This effect is opposed by F2,6BP, which prevents complete shutdown of glycolysis when cellular energy is high (Hers & Van Schaftingen, 1982). Adenosine monophosphate (AMP) is another activator (Passonneau & Lowry, 1962), and therefore the AMP/ATP ratio fine tunes flux through glycolysis in response to the energy state of the cell, which is especially important in tissues like liver or skeletal muscle that experience frequently fluctuating energy levels (Mor, Cheung & Vousden, 2011; Okar & Lange, 1999; Yalcin *et al*., 2009). Other modulators of PFK include F1,6BP (Tornheim, 1980; Boiteux, Hess & Sel'kov, 1980; Van Schaftingen *et al*., 1981), citrate (Parmeggiani & Bowman, 1963; Garland, Randle & Newsholme, 1963; Passonneau & Lowry, 1963), cyclic AMP (cAMP) (Mansour & Mansour, 1962), inorganic phosphate (P_i_) (Passonneau & Lowry, 1962), PEP (Kemp, 1971; Colombo *et al*., 1975), adenosine diphosphate (ADP) (Passonneau & Lowry, 1962), cyclic guanosine monophosphate (cGMP) (Pinilla & Luque, 1981; Beitner, Haberman & Cycowitz, 1977) and 3‐phosphoglycerate (3PG) (Kemp, 1971; Colombo *et al*., 1975).

Eukaryotic and bacterial ATP‐dependent PFKs and archaeal pyrophosphate (PP_i_)‐dependent PFKs are members of the phosphofructokinase superfamily (known as PFK‐A), whereas ATP‐ and ADP‐dependent PFKs from archaea (e. g. the ATP‐PFK from *Desulfurococcus amylolyticus*) (Hansen & Schönheit, 2000) and the minor ATP‐dependent PFK from *Escherichia coli* are members of the ribokinase superfamily (PFK‐B). Bacterial ATP‐dependent PFKs are usually tetramers and are regulated by PEP (inhibition) and ADP (activation) (Blangy, Buc & Monod, 1968). The archaeal ATP‐dependent PFK adopts a tetrameric structure, accepts different nucleoside triphosphates as phosphoryl donors and exhibits no allosteric regulation (Hansen & Schönheit, 2000). ADP‐dependent PFKs have also been reported in archaea (Tuininga *et al*., 1999; Currie *et al*., 2009). Some archaeal ADP‐dependent PFKs can also utilise UDP, GDP, ATP and GTP to a limited extent (Tuininga *et al*., 1999). Recently, ADP‐dependent PFKs of the PFK‐A family have been described in cyanobacteria and alphaproteobacteria (Shen *et al*., 2024); additionally, there are bacterial (e.g. *Amycolatopsis methanolica*), archaeal (e.g. *Thermoproteus tenax*) and eukaryotic (e.g. protists and plants) PFKs that use PP_i_ as phosphoryl donor (Carnal & Black, 1983; Siebers, Klenk & Hensel, 1998; Alves *et al*., 2001; Saavedra *et al*., 2007). These enzymes catalyse a reversible reaction and are involved in both glycolysis and gluconeogenesis (Table 3). Additionally, it was shown that ATP‐dependent PFK can also function in the gluconeogenic direction *in vivo*, even in the presence of FBPase activity, by supramolecular organisation and metabolic channelling in organelle‐like structures, so‐called ‘glycosomes’, in *Trypanosoma brucei* (Plazolles *et al*., 2025).

**Table 3:** Fructose 6‐phosphate phosphorylation in the three domains of life.

Domain	Enzyme	Reaction	Example references
Eukarya	ATP‐PFK‐A	F6P + ATP → F1,6BP + ADP	Dunaway *et al*. (1988)
	ATP‐PFK‐A	F6P + ATP ↔ F1,6BP + ADP	Plazolles *et al*. (2025) in protists
	PP_i_‐PFK‐A	F6P + PP_i_ ↔ F1,6BP + P_i_	Mertens *et al*. (1993) in protists Carnal & Black (1983) in plants
Bacteria	ATP‐PFK‐B	F6P + ATP → F1,6BP + ADP	Wu *et al*. (1991)
	ATP‐PFK‐A	F6P + ATP → F1,6BP + ADP	Hansen *et al*. (2002a)
	ADP‐PFK‐A	F6P + ADP → F1,6BP + AMP	Shen *et al*. (2024) in cyanobacteria and alphaproteobacteria
Archaea	PP_i_‐PFK‐A	F6P + PP_i_ ↔ F1,6BP + P_i_	Alves *et al*. (2001)
	PP_i_‐PFK‐A	F6P + PP_i_ ↔ F1,6BP + P_i_	Siebers *et al*. (1998)
	ATP‐PFK‐B	F6P + ATP → F1,6BP + ADP	Hansen & Schönheit (2000)
	ADP‐PFK‐B	F6P + ADP → F1,6BP + AMP	Verhees *et al*. (2001)
	ADP/ATP/GDP/GTP/UDP‐PFK‐B	F6P + ADP/ATP/GDP/GTP/UDP → F1,6BP + AMP/ADP/GMP/GDP/UMP	Tuininga *et al*. (1999)

This three‐step preparatory phase has trapped glucose within the cell, invested two molecules of ATP, and edited glucose in preparation for the two‐step splitting phase.

##### (iv) Aldolase

The fourth step of glycolysis, which is the splitting stage, involves reversible cleavage of the aldol (i.e. beta‐hydroxy ketone/aldehyde) F1,6BP into the triose phosphate isomers dihydroxyacetone phosphate (DHAP) and glyceraldehyde 3‐phosphate (GAP), catalysed by fructose‐bisphosphate aldolase (FBA, often called simply aldolase). Aldolases are subdivided into two classes based on the reaction mechanism. Class I aldolases use an active‐site lysine residue to generate a protonated Schiff base intermediate that undergoes a retro‐aldol reaction, whereas Class II aldolases use divalent metal cations such as Zn^2+^ for catalysis *via* an enediol intermediate (Marsh & Lebherz, 1992; Perham, 1990) (Table 4). Only class I aldolases occur in mammals. Notably, a non‐enzymatic aldol condensation that occurs in ice and during hydration‐desiccation is accelerated by lysine, indicating that the enzyme mechanism could have had a simple origin in evolution (Messner *et al.,* 2017).

**Table 4:** Splitting of fructose 1,6‐bisphosphate (F1,6BP) into the triose phosphate isomers in the three domains of life.

Domain	Enzyme	Reaction	Example references
Eukarya	class I aldolase	F1,6BP ↔ DHAP + GAP	Warburg & Christian (1943); Bergmeyer & Bernt (1974)
	class II aldolase		Simpson *et al*. (1971) in yeast Galkin *et al*. (2009) in protists Gross *et al*. (1994) in algae
Bacteria	class II aldolase		Heron & Caprioli (1975)
	class I aldolase		Baldwin & Perham (1978)
Archaea	class IA aldolase		Lorentzen *et al*. (2005)
	class II aldolase		Krishnan & Altekar (1991) in halophilic archaea

Class I aldolases are broadly found in eukaryotes, bacteria and archaea, while class II aldolases are less widespread, and have thus far been described in a subset of bacteria, a subset of halophilic archaea (Krishnan & Altekar, 1991), yeast, certain protists and algae (Simpson *et al*., 1971). The archaeal class I aldolases show structural but limited sequence similarity to classical class I aldolases, form a decamer (dimerization of two pentamers) instead of a tetramer, and have been described as a separate class altogether (archaeal type FBA class IA) (Lorentzen *et al*., 2004, 2005). A later study showed that archaea as well as the deeply branching bacterial lineages contain a bifunctional F1,6BP aldolase/phosphatase with both F1,6BP aldolase and F1,6BP phosphatase activity. This enzyme is heat stable and ensures that heat‐labile triosephosphates are converted into heat‐stable F6P (Say & Fuchs, 2010).

##### (v) Triosephosphate isomerase

The fifth step of glycolysis is catalysed by triose phosphate isomerase (TPI). This enzyme interconverts the triose phosphates DHAP and GAP, making DHAP available for further glycolytic breakdown. At equilibrium, 96% of the triose phosphates are present as DHAP. However, the subsequent glycolytic reactions remove GAP, which is replenished by the action of TPI (Berg *et al*., 2002).

TPI has been described as a ‘perfect enzyme’, as its catalytic efficiency (*k*_cat_/*K*_M_) is close to the diffusion‐controlled limit (Knowles & Albery, 1977). Archaeal TPI sequences are approximately 20 amino acids shorter than their bacterial/eukaryotic counterparts. Most mesophilic TPIs are homodimers while higher oligomerization states (i.e. homotetramers) occur in hyperthermophilic archaeal and bacterial TPIs, suggesting a function in thermo‐adaptation (Walden *et al*., 2004) (Table 5). The sequence conservation of TPI is one argument for an early evolutionary origin of a single glycolytic pathway that later split into different lineages.

**Table 5:** Triose phosphate isomerases in the three domains of life.

Domain	Enzyme	Reaction	Example references
Eukarya	homodimeric TPI	DHAP ↔ GAP	Meyerhof & Kiessling (1935); Meyerhof & Beck (1944)
Bacteria	homodimeric TPI		Mathur *et al*. (2006)
	homotetrameric TP		Schurig *et al*. (1995a) in hyperthermophiles
Archaea	homodimeric TPI		Walden *et al*. (2001)
	homotetrameric TPI		Kohlhoff *et al*. (1996) in hyperthermophilic archaea

Thus far in glycolysis, two molecules of ATP have been invested, but no energy in the form of ATP or NADH has been harvested. From one molecule of glucose, two molecules of GAP are now ready to enter the payoff phase.

##### (vi) Glyceraldehyde 3‐phosphate dehydrogenase

The payoff reaction sequence starts with the conversion of GAP to 1,3‐bisphosphoglycerate (1,3BPG) by the sixth enzyme of glycolysis, glyceraldehyde 3‐phosphate dehydrogenase (GAPDH). This is the only redox reaction in the EMP pathway, and it requires the nicotinamide cofactor NAD^+^ in humans (Table 6). GAPDH catalyses two reactions in a bidirectional fashion: the oxidation of GAP to form glycerate with concomitant generation of NADH from NAD^+^, and the condensation of this carboxylic acid with P_i_ to form the acyl phosphate 1,3BPG (Berg *et al*., 2002). GAPDH is sensitive to rising hydrogen peroxide levels. It contains active‐site cysteine residues that undergo oxidation, leading to reversible inactivation of the enzyme (Shenton & Grant, 2003). This reversible and rapidly induced metabolic block involves an evolved structural domain, which functions as a redox relay, through which hydrogen peroxide is scavenged before it can oxidise other proteins (Peralta *et al*., 2015). Once activated, this redox switch creates a bottleneck in glycolysis, leading to accumulation of upstream metabolites and/or increased flux in the adjacent PPP (Ralser *et al*., 2007, 2009). The PPP produces reduced nicotinamide adenine dinucleotide phosphate (NADPH), which is required to counter the oxidative stress that caused the block in glycolysis in the first place (reviewed in Stincone *et al*., 2015). The GAPDH redox switch plays a role in several physiological situations, e.g it protects skin fibroblasts from ultraviolet (UV) radiation (Kuehne *et al*., 2015) and allows cancer cells to survive matrix detachment (Talwar *et al*., 2023).

**Table 6:** Glyceraldehyde 3‐phosphate (GAP) oxidation in the three domains of life.

Domain	Enzyme	Reaction	Example references
Eukarya	GAPDH	GAP + P_i_ + NAD^+^ ↔ 1,3BPG + NADH+H^+^	Warburg & Christian (1939)
		GAP + P_i_ + NAD(P)^+^ ↔ 1,3BPG + NAD(P)H + H^+^	Verho *et al*. (2002) in fungi Wolosiuk & Buchanan (1976) in plants
	GAPN	GAP + NAD(P) + → 3PG + NAD(P)H + H^+^	Zaffagnini *et al*. (2013); Habenicht (1997) in higher plants
Bacteria	GAPDH	GAP + P_i_ + NAD^+^ ↔ 1,3BPG + NADH+H^+^	Kopeckova *et al*. (2020)
	GAPN	GAP + NAD(P) + → 3PG + NAD(P)H + H^+^	Marchal & Branlant (2002); Iddar *et al*. (2005)
Archaea	GAPDH	GAP + P_i_ + NAD^+^ ↔ 1,3BPG + NADH+H^+^	Prüss *et al*. (1993)
		GAP + P_i_ + NAD(P)^+^ ↔ 1,3BPG + NAD(P)H + H^+^	Charron *et al*. (2000)
	GAPN	GAP + NAD(P) + → 3PG + NAD(P)H + H^+^	Ettema *et al*. (2008); Brunner *et al*. (1998)
	GAPOR	GAP + ferredoxin (ox) → 3PG + ferredoxin (red)	van der Oost *et al*. (1998); Mukund & Adams (1995)

In higher plants, archaea and some bacteria the step involving 1,3BPG formation is omitted by employment of a non‐phosphorylating version of GAPDH (GAPN) (Habenicht 1997; Marchal & Branlant, 2002; Ettema *et al*., 2008), or the glyceraldehyde‐3‐phosphate ferredoxin oxidoreductase (GAPOR) (van der Oost *et al*., 1998), both of which directly generate the next intermediate of the EMP pathway, 3PG (described in Section II.1.*b*).

##### (vii) Phosphoglycerate kinase

In the seventh step of glycolysis, phosphoglycerate kinase (PGK) reversibly transfers the phosphoryl group from 1,3BPG to ADP, thus forming 3PG and ATP (Table 7). With its high phosphoryl transfer potential, 1,3BPG is an effective phosphoryl donor, and this process is named ‘substrate‐level phosphorylation’ as it involves transfer from a phosphorylated intermediate – as opposed to the proton gradient‐mediated ATP formation from ADP and P_i_ seen in oxidative cellular respiration (Berg *et al*., 2002; Mitchell, 1961).

**Table 7:** Phosphoglycerate kinase reaction in the three domains of life.

Domain	Enzyme	Reaction	Example references
Eukarya	PGK	ADP + 1,3BPG ↔ ATP + 3PG	Huang *et al*. (1980)
Bacteria	PGK	ADP + 1,3BPG ↔ ATP + 3PG	D'Alessio & Josse (1971)
	not required for glycolysis if GAPN/GAPOR are used		Iddar *et al*. (2005)
Archaea	PGK	ADP + 1,3BPG ↔ ATP + 3PG	Crowhurst *et al*. (2001)
	not required for glycolysis if GAPN/GAPOR are used		Ettema *et al*. (2008)

In higher plants this step is carried out only by the enzymes GAPN, whereas in archaea (and in some bacteria) it can be substituted by GAPN and sometimes also by GAPOR in the catabolic/glycolytic direction as described above and in Section II.1.*b*.

Since two molecules of GAP are generated from one molecule of glucose, the two ATP molecules that are formed by PGK compensate for the two ATPs consumed during the investment phase. The net ATP balance is now zero when the PGK reaction is present.

##### (viii) Phosphoglycerate mutase

In the eighth step of glycolysis, 3PG is isomerized to 2PG (2‐phosphoglycerate). This reversible shuffling of the phosphoryl group between the C‐3 and C‐2 oxygens is catalysed by phosphoglycerate mutase (PGAM). In vertebrates, some eubacteria and yeast, catalysis involves a phosphorylated histidine residue in the active site of PGAM. This phosphoryl group is transferred from the histidine to the C‐2 oxygen of 3PG to give the enzyme‐bound intermediate 2,3‐bisphosphoglycerate (2,3BPG). Next, the C‐3 phosphoryl group is transferred back to the active site histidine of PGAM, restoring its phosphorylation. Thus, in these organisms, the intermediate 2,3BPG is required in catalytic amounts as a cofactor, and the enzyme is termed ‘cofactor‐dependent PGAM’ (dPGAM, Table 8) (Britton & Clarke, 1969; Meyerhof & Kiessling, 1935; Campbell, Watson & Hodgson, 1974; Guerra *et al*., 2004).

**Table 8:** Phosphoglycerate mutase reaction in the three domains of life.

Domain	Enzyme	Reaction	Example references
Eukarya	dPGAM	3PG + P‐enzyme ↔ 2,3BPG + enzyme ↔ 2PG + P‐enzyme	Sakoda *et al*. (1988); Wang *et al*. (2004)
	iPGAM	3PG ↔ 2PG	Zhao & Assmann (2011) in plants Poonperm *et al*. (2003) in invertebrates
Bacteria	dPGAM	3PG + P‐enzyme ↔ 2,3BPG + enzyme ↔ 2PG + P‐enzyme	Foster *et al*. (2010)
	iPGAM	3PG ↔ 2PG	Foster *et al*. (2010)
Archaea	dPGAM	3PG + P‐enzyme ↔ 2,3BPG + enzyme ↔ 2PG + P‐enzyme	Johnsen & Schönheit (2007)
	iPGAM	3PG ↔ 2PG	Johnsen & Schönheit (2007)

Both 2,3BPG cofactor‐dependent (dPGAM) and ‐independent (iPGAM) enzymes have been identified in bacteria (Foster *et al*., 2010) and archaea (Johnsen & Schönheit, 2007). dPGAMs and iPGAMs belong to two different enzyme families and are structurally unrelated, and thus represent convergent evolution. iPGAMs are stimulated by divalent metal ions and are believed to act by reversible intramolecular phosphoryl transfer between the C‐3 and C‐2 oxygens of phosphoglycerate *via* a phosphoserine intermediate (Jedrzejas *et al*., 2000).

##### (ix) Enolase

The penultimate step of glycolysis is catalysed by enolase (ENO, also named phosphopyruvate hydratase), typically a metallo‐enzyme that uses two magnesium ions, which are bound to its active site, to catalyse reversibly the dehydration of 2PG into PEP (Table 9) (Lohmann & Meyerhof, 1934; Kang *et al*., 2008). This dehydration reaction enhances the phosphoryl group transfer potential and thus enables the final step of glycolysis.

**Table 9:** 2‐phosphoglycerate (2PG) dehydration in the three domains of life.

Domain	Enzyme	Reaction	Example references
Eukarya	homodimeric Mg^2+^‐dependent ENO	2PG ↔ PEP + H_2_O	Lohmann & Meyerhof (1934); Hoorn *et al*. (1974)
Bacteria	homodimeric Mg^2+^‐dependent ENO		Spring & Wold (1971)
	octameric ENO		Stellwagen *et al*. (1973) in thermophiles
Archaea	homodimeric Mg^2+^‐dependent ENO		Peak *et al*. (1994)
	octameric ENO		Schurig *et al*. (1995b) in thermophilic archaea

Enolases from all domains of life belong to the highly conserved enolase superfamily (Verma & Dutta, 1994), which suggests that this enzyme was present in the Last Universal Common Ancestor (LUCA) of Bacteria and Archaea (Zadvornyy *et al*., 2015). Enolase has an eight‐strand α/β barrel fold that differs slightly from the conserved barrel topology seen in TPI (Lebioda, Stec & Brewer, 1989), and is mainly present as homodimer but has been observed as an octamer in thermophilic bacteria (Stellwagen, Cronlund & Barnes, 1973) and archaea (Schurig *et al*., 1995b). Some enolases moonlight as regulators of central cellular processes such as transcription, apoptosis and virulence, which are frequently mediated by cell‐surface enolases (Henderson & Martin, 2011).

##### (x) Pyruvate kinase

The tenth and final reaction of the EMP involves substrate‐level phosphorylation *via* the enzyme pyruvate kinase (PK). PK catalyses the transfer of a phosphoryl group from the highly reactive enol PEP to ADP, forming the more stable ketone pyruvate and ATP. Hence, only in the final step does glycolysis deliver a net yield of two molecules of ATP per molecule of glucose. The thermodynamic equilibrium of this reaction strongly favours pyruvate by a factor of around 10^4^ (Nageswara Rao, Kayne & Cohn, 1979). Hence, under physiological conditions, PK operates exclusively in the direction of glycolysis.

PK is ubiquitous across the three domains of life, and is a homotetrameric protein composed of 50‐kDa subunits. Most eukaryotic and bacterial PKs require monovalent cations such as K^+^ or NH_4_^+^ for optimal activity. Moreover, the dependency on various allosteric regulators, including the aforementioned F2,6BP (e.g. in *Trypanosoma brucei*; van Schaftingen, Opperdoes & Hers, 1985), and various sugar phosphates (Table 10), places the PK reaction among the most regulated steps in the EMP pathway (Berg *et al*., 2002). Furthermore, PK activity can be stress responsive. The human PK isoform PKM2, which is expressed in most tissues except skeletal muscle, and its yeast paralogue PYK2, for instance, have oxidation‐sensitive thiol groups that reduce the enzyme's activity under oxidative stress conditions (Irokawa *et al*., 2021).

**Table 10:** Phosphoenolpyruvate (PEP) to pyruvate conversion in the three domains of life.

Domain	Enzyme	Described reaction specifics	Example references
Eukarya	PK	PEP + ADP → pyruvate + ATP requires monovalent cations (K^+^, NH_4_^+^) allosteric regulation by sugar phosphates	Tietz & Ochoa (1958)
	PPDK	PEP + AMP + PP_i_ ↔ pyruvate + ATP + P_i_	Bruderer *et al*. (1996) in protists
Bacteria	PK	PEP + ADP → pyruvate + ATP requires monovalent cations (K^+^, NH_4_^+^) allosteric regulation by sugar phosphates	Veith *et al*. (2013)
	PPDK	PEP + AMP + PP_i_ ↔ pyruvate + ATP + P_i_	Reeves *et al*. (1968)
	PEPS	Pyruvate + ATP + H_2_O → PEP + AMP + P_i_	Cooper & Kornberg (1969)
Archaea	PK	PEP + ADP → pyruvate + ATP no requirement for monovalent cations (K^+^, NH_4_^+^) no allosteric regulation by sugar phosphates	Schramm *et al*. (2000); Johnsen *et al*. (2003)
	PPDK	PEP + AMP + PP_i_ ↔ pyruvate + ATP + P_i_	Tjaden *et al*. (2006)
	PEPS	Pyruvate + ATP + H_2_O → PEP + AMP + P_i_ PEP + AMP + Pi → pyruvate + ATP + H_2_O	Tjaden *et al*. (2006); Haferkamp *et al*. (2019) Hutchins, Holden & Adams ( 2001); Sakuraba *et al*. (2001) in Thermococcales

Conversely, many archaeal PKs do not require monovalent cations or undergo allosteric regulation by these effectors; exceptions include the AMP‐activated PK from *Thermoplasma acidophilum* (Potter & Fothergill‐Gilmore, 1992; Schramm *et al*., 2000; Johnsen, Hansen & Schönheit, 2003), the ATP‐ and isocitrate‐inhibited PK from *Sulfolobus solfataricus* (Haferkamp *et al*., 2019) and the 3PG‐activated PK from *Archaeoglobus fulgidus* (Solomons *et al*., 2013).

There are two other pathways for the interconversion of PEP and pyruvate: pyruvate, phosphate dikinase (PPDK) and PEP synthetase (PEPS). PPDK catalyses a reversible reaction with a bias towards glycolysis in some species (e.g. *Thermoproteus tenax* and *Giardia lamblia*), and a gluconeogenic function has been proposed in plants. This enzyme is found in all three domains of life alongside PP_i_‐dependent PFK. PP_i_ has been considered to be a cellular waste product that is quickly hydrolysed by cytoplasmic pyrophosphatases to drive anabolic reactions (e.g. DNA synthesis) (Kornberg, Rao & Ault‐Riché, 1999). However, PP_i_ and polyphosphates have also been proposed as primordial energy carriers in a pre‐ATP world (Nicholls *et al*., 2023), and participate in various pyrophosphorolysis reactions (Rozovskaya *et al*., 1989). Furthermore, many organisms utilising PP_i_ as phosphoryl donor in glycolysis also produce a V‐type H^+^‐translocating pyrophosphatase (H^+^‐PP_i_‐ase), which allows for the establishment of a proton‐motive force by PP_i_ hydrolysis and thus ATP formation *via* ATP synthase (Drozdowicz & Rea, 2001).

Most bacterial and archaeal PEPS operate in the reverse, anabolic direction. However, the reaction is close to thermodynamic equilibrium, and a catabolic function has been observed in the PEPS from hyperthermophilic archaea like *Pyrococcus furiosus* and *Thermococcus kodakarensis* (Hutchins, Holden & Adams, 2001; Sakuraba *et al*., 2001). It is proposed that production of ATP from AMP *via* PEPS, rather than from ADP *via* PK, is used in the glycolytic direction to avoid competition with sugar kinases for ADP and to enhance glycolytic flux. The net yield of the modified glycolytic pathway in *Thermococcales* thus varies between zero and two ATPs depending on the use of PK or PEPS. AMP can be interconverted with ATP to two molecules of ADP in a reaction catalysed by adenylate kinase (ATP + AMP ↔ 2ADP), providing energy recharge as well as ADP for the kinase reactions. The anabolic PEPS from *Thermoproteus tenax* and *Sulfolobus solfataricus* are inhibited by AMP and α‐ketoglutarate (Tjaden *et al*., 2006; Haferkamp *et al*., 2019). PEPS is absent in eukaryotes, which instead use PEP carboxykinase to catalyse the GTP‐ or ATP‐dependent reversible decarboxylation of oxaloacetate (OAA) to PEP. PEP carboxykinase is also found in bacteria and has been identified in a few archaea (Fukuda *et al*., 2004). These studies in archaea suggest that there is a close link between the phosphoryl donor utilised in the preparatory phase and the energy‐generating reaction used at the level of PEP–pyruvate conversion in order to optimise glycolytic flux and energy gain [for more detailed discussion, see Imanaka *et al*. (2006), Bräsen *et al*. (2014) and Haferkamp *et al*. (2019)].

#### (b) Variants of the EMP pathway

**Fig. 2:**
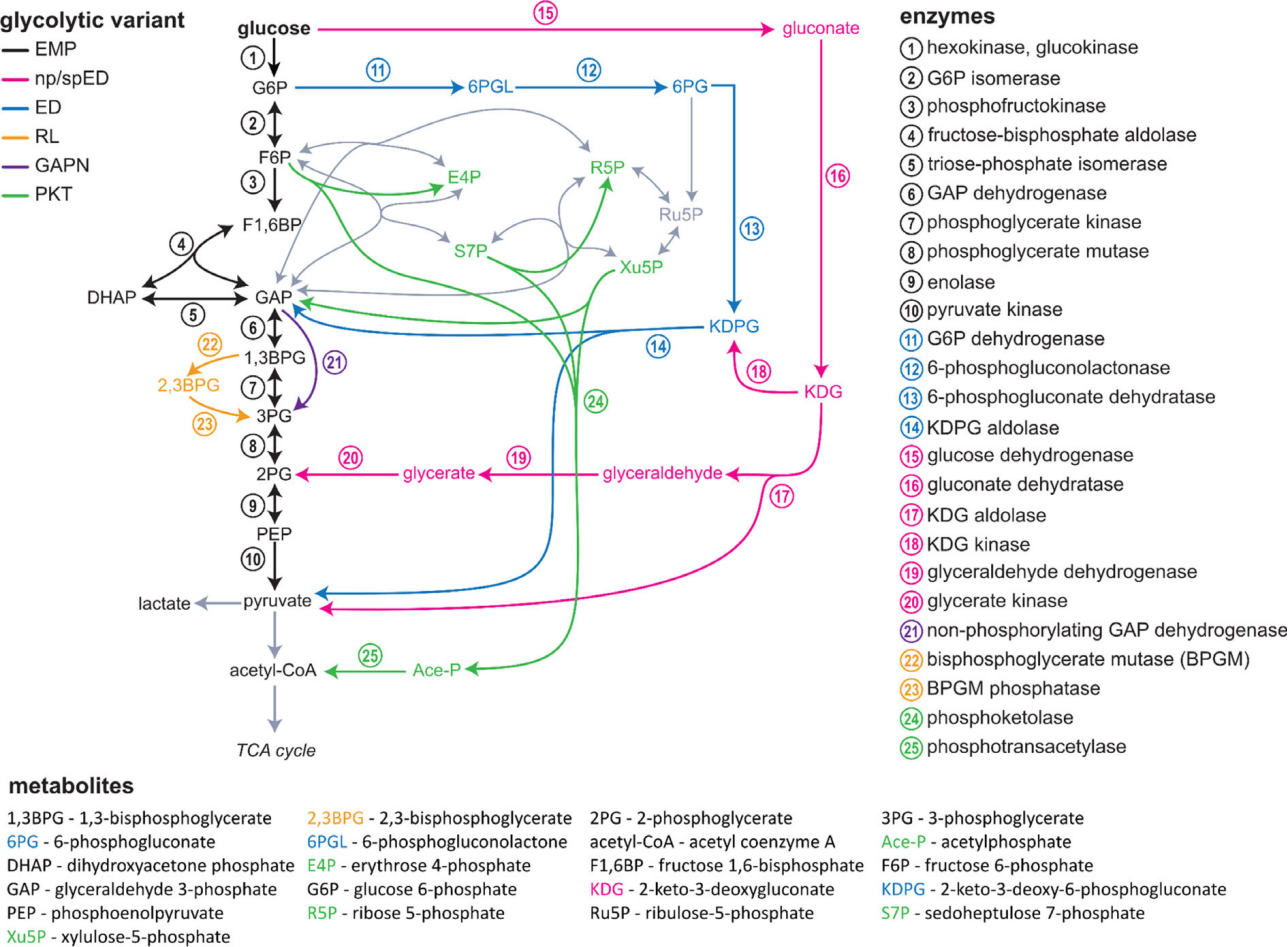
The Embden–Meyerhof–Parnas (EMP, in black) pathway and alternative glycolytic pathways found in the three domains of life. The Entner–Doudoroff pathway (ED, in blue) is typical of aerobic bacteria and diverges from the EMP pathway after the first step of glucose phosphorylation. Archaea exploit two variations of the ED pathway, i.e. the non‐phosphorylative ED pathway (npED, in pink) and the semi‐phosphorylative ED pathway (spED, also in pink) or combinations thereof. The first two joined reactions convert glucose into 2‐keto‐3‐deoxy‐gluconate (KDG), then the spED merges into the classical ED pathway by phosphorylation of KDG to 2‐keto‐deoxy‐6‐phosphogluconate (KDPG), while the npED proceeds to eventually convert KDG into pyruvate and 2‐phosphoglycerate, thus merging into the lower part of the EMP pathway. The bifunctional KD(P)G aldolase would cover reaction 14 and 17. The Rapoport–Luebering shunt (RL, in yellow) is a variant of the EMP pathway which enables red blood cells to produce 2,3‐bisphosphoglycerate (2,3BPG), mediating oxygen release from haemoglobin. A further variant of the EMP pathway found in some plants employs a non‐phosphorylating version of GAPDH (GAPN, in purple) which bypasses the formation of 1,3‐bisphosphoglycerate (1,3BPG), whereas in archaea (and some bacteria) the bypass can involve GAPN and sometimes GAPOR. Finally, the phosphoketolase or Bifido shunt (PKT, in green) includes a group of reactions that allow lactic acid bacteria of the human gut to convert intermediates of upper glycolysis directly into acetyl‐CoA.

In red blood cells, the Rapoport–Luebering (RL) shunt operates in parallel with the EMP pathway, bypassing PGK and thus substrate‐level phosphorylation. Therefore, in contrast to the EMP pathway, no ATP is produced for the proportion of the flux that goes through the RL shunt. The shunt is carried out by the multifunctional enzyme bisphosphoglycerate mutase (BPGM). BPGM catalyses the formation of 2,3‐bisphosphoglycerate (2,3BPG, also named 2,3‐diphosphoglycerate) from 1,3BPG and also acts as a phosphatase, promoting the hydrolysis of 2,3BPG to 3PG (Rapoport & Luebering, 1952, 1951) (Fig. 2). The generated 2,3BPG allosterically affects the conformation of haemoglobin chains in red blood cells and thereby helps to release oxygen from haemoglobin, ensuring efficient oxygen delivery to tissues (Chiarelli *et al*., 2012).

A variant of the EMP pathway that possesses a non‐phosphorylating version of GAPDH (GAPN) is found in some higher plants (Zaffagnini *et al*., 2013; Habenicht, 1997) and in most (hyper)thermophilic archaea. This NAD(P)^+^‐dependent enzyme oxidises GAP directly to 3PG to generate NADPH for biosynthetic processes and thereby bypasses the phosphorylating GAPDH‐catalysed reaction (Fig. 2). GAPN also occurs in bacteria such as *Streptococcus mutans* (Marchal & Branlant, 2002; Iddar *et al*., 2005) and belongs to the aldehyde dehydrogenase superfamily (Brunner, Siebers & Hensel, 2001; Reher, Gebhard & Schönheit, 2007). It has no close functional or structural relationships with phosphorylating GAPDHs (Habenicht, Hellman & Cerff, 1994). Anaerobic archaea use the glyceraldehyde‐3‐phosphate ferredoxin oxidoreductase (GAPOR) as well (Mukund & Adams, 1995). The use of unidirectional GAPN or GAPOR instead of GAPDH/PGK provides a higher thermodynamic driving force but omits substrate‐level phosphorylation, and thus glycolysis produces no ATP. This pathway avoids formation of the most thermolabile intermediate in glycolysis, 1,3BPG, and is therefore favoured in hyperthermophilic archaea (Schmerling *et al*., 2022).

#### (c) The PKT/Bifido‐shunt

The enzyme phosphoketolase (PKT) promiscuously cleaves xylulose 5‐phosphate (Xu5P), F6P or sedoheptulose 7‐phosphate to generate acetyl‐phosphate (Ace‐P) and the aldose phosphates GAP, erythrose 4‐phosphate (E4P) and ribose 5‐phosphate (R5P), respectively (Tittmann, 2014). Although most PKT enzymes exhibit higher specificities towards Xu5P and F6P, some variants act on all three substrates (Krüsemann *et al*., 2018) (Fig. 2). Through these reactions, PKT provides an alternative pathway to EMP glycolysis for glucose metabolism. The pathway is mainly found in lactic acid bacteria that populate the human gut (Palframan, Gibson & Rastall, 2003). The combined activities of PKT (Krüsemann *et al*., 2018) allow increased production of acetyl‐CoA from glucose *via* Ace‐P, which has inspired research to establish the so‐called non‐oxidative glycolysis (NOG) pathway (Lin *et al*., 2018; Bogorad, Lin & Liao, 2013).

#### (d) The classical ED pathway in prokaryotes

The ED pathway is often preferred by aerobes, and might be evolutionarily older than the EMP pathway (Romano, Saier & Mortlock, 1992; Conway, 1992; Romano & Conway, 1996). This oxidative pathway uses four enzymes that are distinct from the EMP pathway: G6P dehydrogenase (G6PD, or Zwf), 6‐phosphogluconolactonase (PGL), 6‐phosphogluconate dehydratase (Entner–Doudoroff dehydratase, Edd) and 2‐keto‐3‐deoxy‐6‐phosphogluconate (KDPG) aldolase (Entner–Doudoroff aldolase, Eda). These enzymes replace PGI, PFK, FBA and TPI used in the EMP pathway. As in the EMP pathway, the classical ED pathway starts with the phosphorylation of glucose to G6P, catalysed by HK. In the next step however, G6P is oxidised by Zwf to form 6‐phosphogluconolactone, which is subsequently hydrolysed spontaneously or by the activity of PGL to 6‐phosphogluconate, as in the PPP. Notably, the isomeric compound, γ‐6‐phosphogluconolactone, a side product of the oxidative phase of the PPP, is a signalling molecule that leads to activation of AMP kinase. Next, Edd catalyses the dehydration of 6‐phosphogluconate into KDPG, the characteristic metabolite of the ED pathway. KDPG possesses important regulatory functions (e.g. Wang *et al*. 2023; Campilongo *et al*., 2017), while elevated KDPG levels are reported to be toxic for the cell (Conway, 1992). KDPG is cleaved by the aldolase Eda into GAP and pyruvate. Pyruvate is channelled into the common lower part of the EMP pathway, while GAP enters the payoff phase of glycolysis. Unlike the EMP pathway, the ED pathway therefore produces only one molecule each of ATP, NADPH (by Zwf) and NADH (by GAPDH) from each glucose molecule (Conway, 1992). ED‐dependent organisms can supplement their ATP yield through aerobic respiration (Spector, 2009). Utilisation of the classical ED pathway is thought to exhibit an exergonic advantage at a lower proteomic cost, and it produces more NADPH for anabolic purposes and metabolites with essential regulatory functions (i.e. KDPG). There is evidence that the ED pathway is used by prokaryotes with greater access to non‐glycolytic energy sources; the ED pathway can also take over an alternative function when both the ED and EMP pathways are present in the same organism. Thus, in *Escherichia coli*, the ED pathway is used for utilisation of gluconate but not of glucose, which favours the EMP pathway instead (Eisenberg & Dobrogosz, 1967). Notably, growth on gluconate compared to glucose enhances the metabolic flux to pyruvate while halving the production of NADH and ATP.

The net equation for glycolysis through the ED pathway is:

$$\text{glucose}+\mathrm{ADP}+P_{i}+{\mathrm{NAD}}^{+}+{\text{NADP}}^{+}\to 2\;\text{pyruvate}+\mathrm{ATP}+\text{NADH}+\text{NADPH}+H^{+}+H_{2}O$$

#### (e) Variants of the ED pathway

Whereas the classical ED pathway is commonly preferred in bacteria, variants of the ED pathway are found in all three domains of life, with Archaea lacking the classic ED altogether. Variations of the ED pathway include the non‐phosphorylative ED (npED) and semi‐phosphorylative ED (spED) pathways as well as a combination of both in a branched pathway (for review see Bräsen *et al*., 2014). In these modified pathways, the initial phosphorylation of glucose is omitted. Instead, glucose is oxidised by glucose dehydrogenase to yield NADPH and gluconate, which is subsequently converted to 2‐keto‐3‐deoxygluconate (KDG) (Spaans *et al*., 2015). KDG represents the branching point of the different ED pathway variants (Siebers *et al*., 2004).

In the npED pathway, no phosphorylation of KDG takes place. Instead, KDG is cleaved by KDG aldolase to form pyruvate and glyceraldehyde (Fig. 2). The latter is oxidised to glycerate in a reaction catalysed by either an NAD(P)^+^‐ or a ferredoxin‐dependent dehydrogenase (Kehrer *et al*., 2007). Glycerate is then phosphorylated by ATP‐dependent glycerate kinase to 2PG, which then follows the EMP pathway to pyruvate. The npED pathway has been reported in thermoacidophilic archaea and some bacteria (De Rosa *et al*., 1984; Bräsen *et al*., 2014).

In the spED pathway, KDG is phosphorylated to KDPG by KDG kinase, connecting this pathway with the canonical ED pathway. The spED pathway operates in extreme halophilic Euryarchaea and Clostridia (Johnsen *et al*., 2001; Hochstein, 1974). A modified branched pathway with both npED and spED being active was observed in (hyper)thermophilic archaea such as *Sulfolobus* species and *Thermoproteus tenax*. Notably, the aldolase is active on KDG and KDPG thus representing a bifunctional KD(P)G aldolase. There is also some bioinformatic evidence that this branched ED pathway is common in archaea (Bräsen *et al*., 2014).

#### (f) Other glycolysis pathways

Besides the natural alternatives to the canonical glycolytic pathways, several other options exist that naturally do not involve glycolysis but instead act mainly as sinks preventing metabolite accumulation (e.g. multiple routes to generate pyruvate) (Feuer *et al*., 2012). Some of these routes have been engineered as synthetic pathways to replace EMP glycolysis, including pathways *via* methylglyoxal or involving serine biosynthesis and degradation (Iacometti *et al*., 2022).

#### (g) Extending glycolysis to sulfosugar metabolism: sulfoglycolysis

**Fig. 3:**
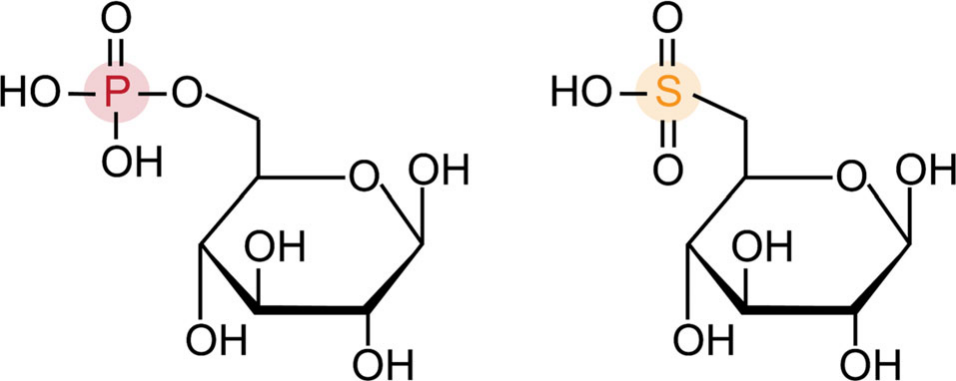
Glucose 6‐phosphate (G6P, left) and sulfoquinovose (SQ, right) differ by the replacement of phosphate with a sulfonate.

Diverse environmental and gut bacteria utilise sulfoglycolytic pathways to catabolise the sulfosugar sulfoquinovose (6‐deoxy‐6‐sulfo‐D‐glucose; SQ) as a source of carbon, energy (as ATP) and reducing power (as NAD(P)H) (Snow *et al*., 2021) (Fig. 3). SQ is primarily produced by photosynthetic organisms as part of sulfolipid biosynthesis, and is therefore present where plant and algal matter is degraded, e.g. in the soil and oceans, and is assimilated as a component of plant‐ and algae‐based food (Snow *et al*., 2021).

**Fig. 4:**
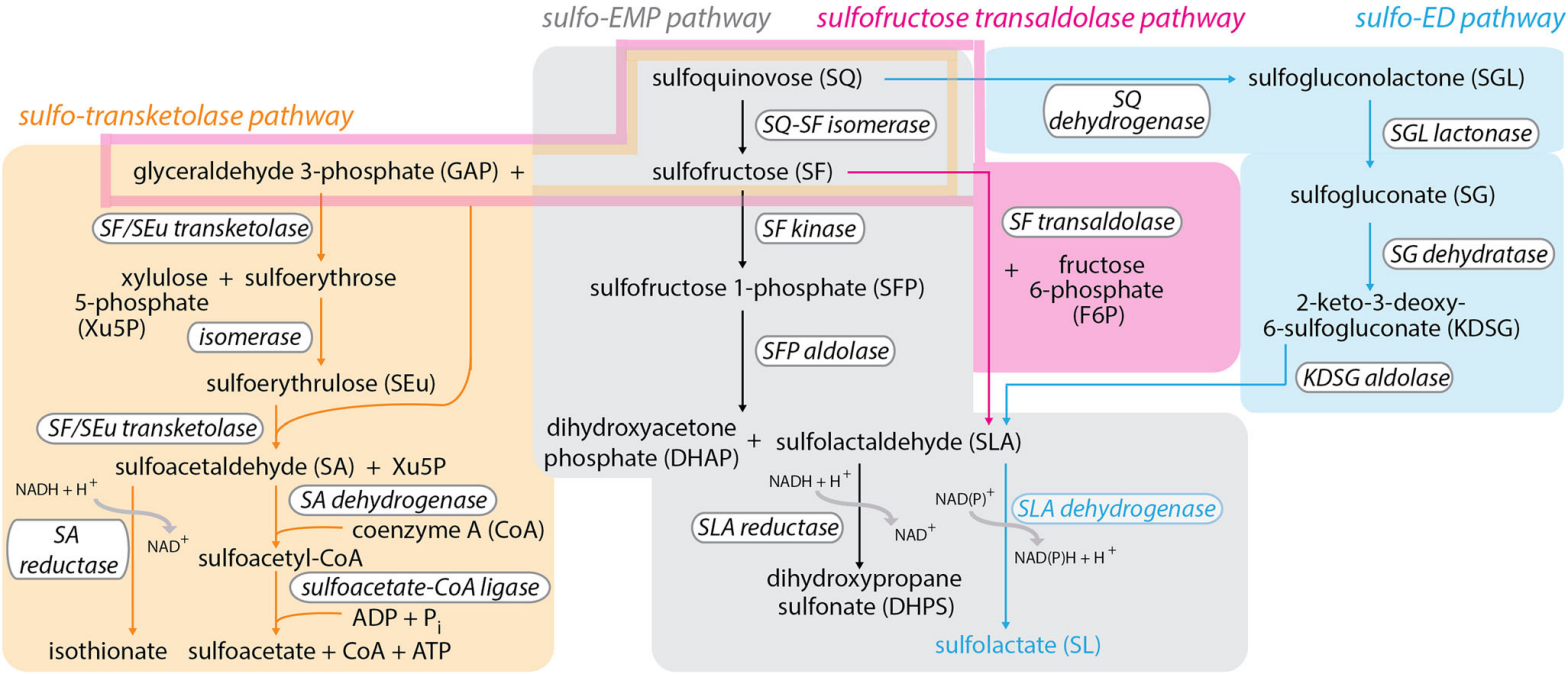
Sulfoglycolytic pathways and the catabolism of sulfoquinovose (SQ). Environmental and gut bacteria can break down SQ into a series of products: dihydroxypropane sulfonate (DHPS) by the sulfo‐EMP pathway, sulfolactate (SL) by either the sulfo‐ED pathway or by the sulfofructose transaldolase pathway, or isothionate or sulfoacetate by the sulfo‐transketolase pathway.

Bacterial sulfoglycolytic pathways are unusual because they are purely catabolic and operate exclusively in the cleavage direction, as all intermediates possess the sulfonate group and cannot be used in other pathways. Thus, there is no reverse pathway analogous to gluconeogenesis known to date, and no sulfonated intermediates are siphoned off to feed into other pathways. Four sulfoglycolytic pathways have been described that mirror features of the classical bacterial EMP, ED and PPP pathways (Fig. 4), but with a sulfonate group in place of the phosphate of G6P. As the sulfonate is inherent in the substrate, all of these pathways lack an equivalent of hexokinase.

The sulfo‐EMP pathway in *Escherichia coli* splits the 6‐carbon chain of SQ into two 3‐carbon fragments, sulfolactaldehyde (SLA) and DHAP, and generates 2,3‐dihydroxypropane sulfonate (DHPS) (Fig. 4). DHPS does not undergo further metabolism in this organism and is excreted (Denger *et al*., 2014). In some organisms, SLA is oxidised to sulfolactate (SL) and then excreted (Kaur *et al*., 2022).

The sulfofructose (SF) transaldolase pathway also begins with SQ conversion to SF, but then SF undergoes a SF transaldolase‐catalysed reaction with GAP to produce F6P and SLA. SLA is subsequently either oxidised to SL or reduced to DHPS, both of which are excreted (Frommeyer *et al*., 2020).

The sulfo‐ED pathway converts SQ, via 2‐keto‐3‐deoxy‐6‐sulfogluconate (KDSG), to SL which is excreted (Felux & Spiteller, 2015).

In all cases, as a result of excreting half the carbon of SQ as SL or DHPS, sulfoglycolytic yields of pyruvate/DHAP (or F6P in the SF transaldolase pathway) are halved compared to glycolysis of glucose. The effects on ATP and NADH synthesis are more complex. In the sulfo‐EMP pathway, NADH is consumed by the reduction of SLA to DHPS and produced by its oxidation to SL, with the outcome for the SF transaldolase pathway dependent on whether the pathway operates in aerobic (SL; production of NADH) or anaerobic (DHPS; consumption of NADH) bacteria.

Finally, the sulfo‐transketolase (sulfo‐TK) pathway proceeds from SQ, *via* sulfoerythrose and sulfoerythrulose (SEu) to either isethionate + NAD^+^ (Liu *et al*., 2021) or sulfoacetate + coenzyme A (CoA) + ATP (Chu *et al*. 2023). This reaction splits two carbon fragments from the 6‐carbon chain of SQ. The end products, isethionate or sulfoacetate, are excreted.

Evidently sulfoglycolytic organisms use gluconeogenesis to satisfy the demands of the PPP and cell‐wall biogenesis (Mui *et al*., 2023). It is therefore likely that all sulfoglycolytic enzymes exhibit high selectivity for the sulfonated substrate variant while the unidirectional gluconeogenic enzymes exhibit high selectivity for the phosphorylated intermediates. Enzymes of the sulfo‐EMP pathway exhibit high specificity for sulfonated substrates *versus* the analogous phosphorylated species from glycolytic/gluconeogenic metabolism (Sharma *et al*., 2020, 2021).

SQ is also metabolised through sulfolytic pathways that involve the direct cleavage of the C–S bond to produce sulfite and glucose. In these pathways, the key enzymes are flavin‐dependent SQ monooxygenases or Fe^2+^ and α‐ketoglutarate dependent dioxygenases that convert SQ to 6‐dehydroglucose (glucose‐6‐aldehyde) and an NADPH‐dependent reductase that reduces 6‐dehydroglucose to glucose, which can enter glycolysis (Liu *et al*., 2021; Sharma *et al*., 2022; Mui *et al*., 2023).

#### (h) Glycolysis in plants

**Fig. 5:**
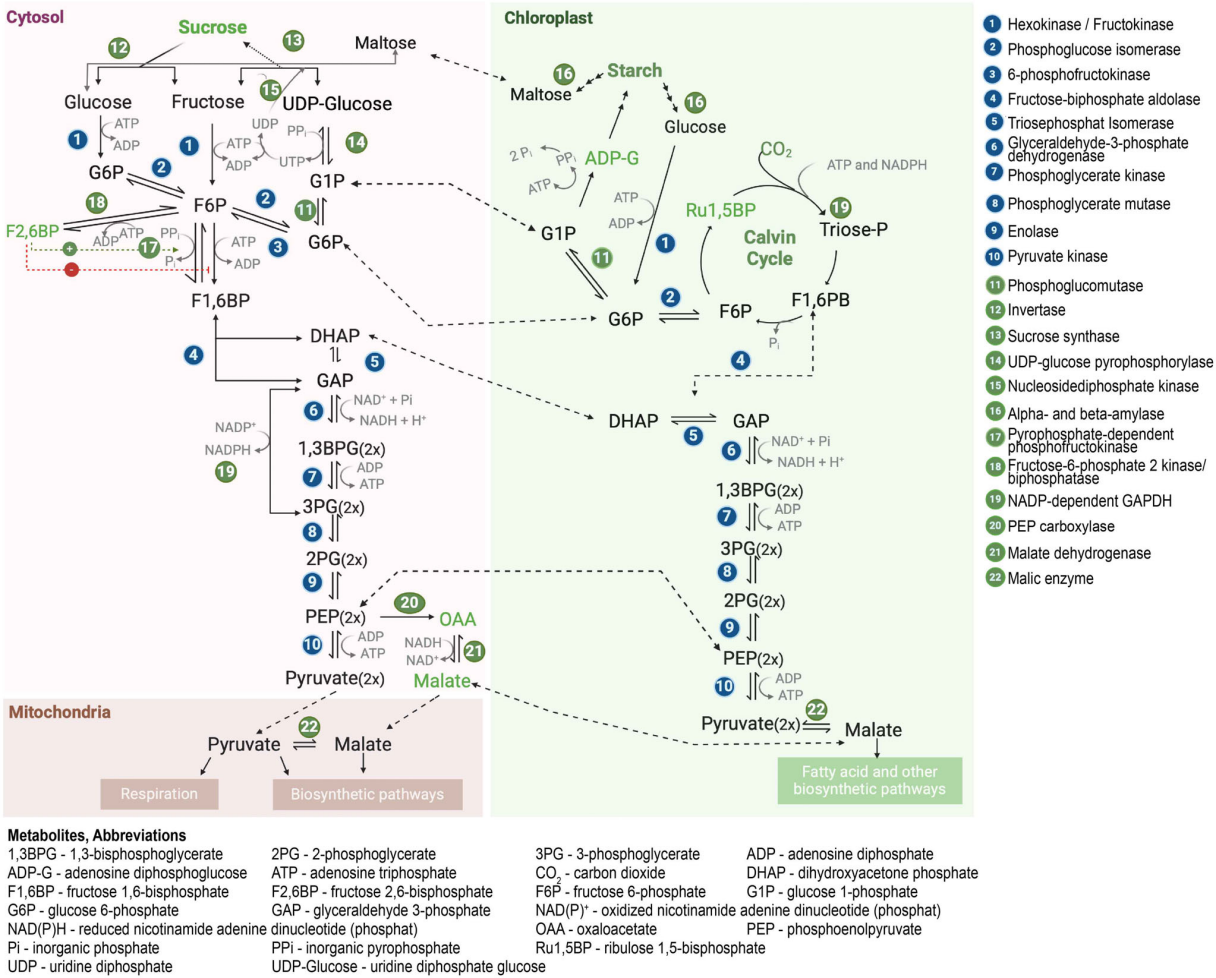
Compartmentalisation and regulation of glycolysis in plants. Plants conduct glycolysis in both the cytosol (left) and chloroplasts/plastids (right).

Glycolytic pathways in photoautotrophic plants are, next to the PPP, intertwined with the Calvin–Benson–Bassham (CBB) cycle (Bassham *et al*., 1954) that facilitates carbon fixation from aerial CO_2_ on the basis of sugar phosphate intermediates, and show adaptations to different cellular compartments. The triose phosphates from the CBB cycle in the chloroplast are used either for glycolysis or for generating the main substrates of plant glycolysis: sucrose in the cytosol and starch in the chloroplast. Glycolysis in plants is compartmentalised both physically and functionally between cytosol and plastids (Rees & Ap Rees, 1988). Despite the similarity of the chloroplastic, plastidic and cytosolic isoenzymes, differences in their kinetic and regulatory properties allow simultaneous action of these glycolytic pathways without competing for the same substrates, thereby optimising enzymatic reactions and preventing futile cycles (Linka & Weber, 2010). Plastidic glycolysis operates independently of light in a linear pathway, supplying energy and carbon to non‐photosynthetic cells (e.g. roots) and supporting biosynthetic processes such as fatty acid and amino acid synthesis. On the other hand, chloroplastic glycolysis is intricately connected to photosynthesis, recycling CBB cycle intermediates and maintaining carbon flux within light‐dependent metabolism. This process, regulated by metabolites and the redox status generated by light reactions, predominantly takes place in autotrophic cells, such as those in leaves. During photosynthesis, carbon dioxide (CO_2_) assimilation produces 3PG, which is converted into triose phosphates (Fig. 5).

In contrast to the plastidic glycolytic pathway, the complete set of enzymes in the chloroplastic pathway has been debated, as the generation of 3PG could also be seen as an overflow of photosynthesis, rather than a product of glycolysis (Anoman *et al*., 2015). Although the cytosolic and plastidic glycolytic pathways are independently regulated, their integration into cell metabolism is achieved by highly selective transporters present in the inner plastid envelope, allowing the exchange of intermediates such as G1P, G6P, DHAP, 3PG and PEP. In chloroplasts and non‐photosynthetic plastids, glycolysis uses mainly starch, a polymer of glucose, as substrate. The actions of different enzymes are required to release glucose monomers that must first be phosphorylated by HK to proceed into glycolysis (Streb & Zeeman, 2012).

The cytosolic glycolytic pathway is driven by a complex network of bypass reactions that increase ATP yield. The presence of alternative reactions controlling the metabolisation of sucrose, F6P, PEP and GAP increases plasticity of the network, enabling metabolic acclimation during drastic environmental or developmental changes. One important aspect of this flexibility is the use of the pyrophosphate/orthophosphate (PP_i_/P_i_) system instead of ATP/ADP in energy transduction *via* sucrose synthase and PP_i_‐dependent PFK (Plaxton, 1996; Givan, 2007). The levels of PP_i_, a byproduct of anabolism, are high in the cytosol due to the lack of pyrophosphatase, resulting in stable concentrations regardless of environmental conditions. During photosynthesis, triose phosphates generated by the CBB cycle are exported to the cytosol *via* the triose phosphate/phosphate translocator (TPT), which simultaneously imports inorganic phosphate (P_i_) into the chloroplast. In the cytosol, triose phosphates are utilised for sucrose synthesis. A reduction in chloroplastic P_i_ levels limits the export of triose phosphates, redirecting carbon flux towards starch synthesis within the chloroplast. As the principal substrate for glycolysis in the cytosol, sucrose is typically hydrolysed by the ATP‐dependent invertase to release glucose and fructose (Koch, 2004), which are respectively phosphorylated by HK and fructokinase and then enter glycolysis. However, sucrose synthase becomes the preferred route under energy‐limited conditions as it uses PP_i_ and UDP rather than ATP (Xu *et al*., 1989).

The reversible interconversion of F6P and F1,6BP, catalysed by PFK, is an exclusive and key regulatory step in cytosolic glycolysis. Its activity is promoted *via* allosteric regulation by the metabolite F2,6BP. Similar to mammalian cells, the levels of this metabolite are governed by the bifunctional enzyme F2KP, which possesses both 6‐phosphofructo‐2‐kinase as well as fructose‐2,6‐bisphosphatase activities and interconverts F2,6BP and F6P (Huber, 1986). F2,6BP promotes glycolysis by activating PFK and suppressing cytosolic FBPase (cyt‐FBPase) in non‐photosynthetic organisms; however, this regulation appears to be less relevant in plants (Nielsen, Rung & Villadsen, 2004). In plants, F2,6BP plays a distinct role by activating pyrophosphate‐dependent phosphofructokinase (PFP), which is critical for coordinating carbon partitioning during photosynthesis (Nielsen *et al*., 2004). The cytosolic P_i_:3PG ratio induces the synthesis of F2,6BP. During starch mobilisation at night, maltose or hexose sugars are exported from the chloroplast, bypassing the need for cyt‐FBPase and regulation by F2,6BP in sucrose synthesis. PFP becomes activated under energy‐limiting conditions or elevated cytosolic pyrophosphate (PP_i_) levels, providing an ATP‐independent pathway to sustain glycolytic flux. This mechanism ensures metabolic plasticity during stress or periods of high metabolic demand.

Another feature in plant metabolism is the higher demand for NADPH to support various biosynthetic processes. As sessile organisms, they depend on photosynthesis and their surrounding environment, leading to greater fluctuations in energy and redox states compared to other organisms. In this context, the cytosolic NADP‐dependent GAPDH plays a crucial role in maintaining redox balance by recycling NADPH. This function complements plastidic pathways, reinforcing metabolic flexibility and integration across cellular compartments. The glycolytic intermediate PEP, a branching point for many primary and secondary metabolic pathways, can be transformed into OAA by PEP carboxylase (PEPC) (Bandurski & Greiner, 1953; O'Leary, 1982). Reduction of OAA into malate by malate dehydrogenase (MDH) allows malate to enter the mitochondria or plastids, where it can be decarboxylated to pyruvate, bypassing the PK reaction. This alternative route occurs under stress conditions when ADP is rate‐limiting for PK activity (Burnell & Hatch, 1988).

A notable peculiarity of the cytosolic glycolytic pathway in plants is its bottom‐up regulation, including the draining of PEP by PK and PEPC and the interconversion of F6P and F1,6BP by PFK and pyrophosphate‐dependent phosphofructokinase (Plaxton, 1996; Givan, 2007). Increased activity of PK or PEPC minimises the inhibition of PFK, allowing hexose phosphate flux into glycolysis. By contrast, reduced PEP or 3PG levels alleviate F2KP inhibition and consequently increase F2,6BP levels. This regulation permits plants to coordinate glycolytic flux to pyruvate independently of related processes such as the CBB cycle and sucrose–triose phosphate–starch conversion.

### (2) The evolutionary origins of glycolytic reactions

Why do all organisms use glucose, and why do even the most divergent glycolytic reaction sequences share topological properties? The deep roots of glycolytic reactions within the metabolic network of every living organism indicate their ancient evolutionary origins (Romano & Conway, 1996; Jeong *et al*., 2000). Protein sequences and tertiary structures of glycolytic enzymes have been used to characterise genetic and structural relationships across species as an estimate of their evolutionary distance (Fothergill‐Gilmore & Michels, 1993), revealing that the core structures of some of the glycolytic enzymes had an early origin, evolving from a limited set of precursor domains with simpler functions (Rossman, 1981). This notion is supported by the tight entanglement between glycolytic and gluconeogenic reactions and the CBB cycle in plants and cyanobacteria, whose evolutionary predecessors drove the oxygenation of our planet (Nisbet *et al*., 2007). Glycolytic enzymes also have much simpler structural characteristics in comparison to the complexity of catabolic ATP generation through oxidative phosphorylation. Additionally, indispensable building blocks would have been necessary also for the earliest proposed life scenarios (Hordijk, Hein & Steel, 2010). As sugars and sugar phosphates are integral components of the backbones of RNA and DNA alike, these intermediates must have been either present in abundance (Kitadai & Maruyama, 2018; Schönheit, Buckel & Martin, 2016), or replenished by a continuous, pre‐biochemical process (Ralser, 2018). The supply of such building blocks is crucial for a living and self‐replicating system and is universally provided by glycolysis and the PPP in modern organisms, indicating their earlier development.

In light of these points, how could glycolysis have arisen? At the level of metabolism, it is important to note that positive selective pressure can only be driven by functional products (Ralser, 2014; Horowitz, 1945). This means that a metabolic pathway such as glycolysis could not develop sequentially, enzyme by enzyme, if only dead‐end intermediates were formed. Instead, the reaction products of every individual evolutionary step would be needed to confer a selective advantage in the first place (Noda‐Garcia, Liebermeister & Tawfik, 2018; Ralser, 2018).

The origin of glycolysis has been systematically evaluated by studying the intrinsic chemical reactivity of glycolytic intermediates in reaction environments similar to the geochemical environment of the early Earth (Keller, Turchyn & Ralser, 2014; Keller *et al*., 2016). These iron‐rich and oxygen‐poor conditions facilitate glycolysis‐like, non‐enzymatic molecular interconversions, stabilising and accelerating glycolysis‐like reactions along specific pathways. Intriguingly, these routes form a pathway structure reminiscent of the metabolic network organisation in extant species. There is evidence of the formation of key components under plausible mild conditions reflecting the environment of our cells, including glycolytic reactions (Coggins & Powner, 2017) and other core metabolic pathways including gluconeogenesis and the citric acid cycle (Messner *et al*., 2017; Keller *et al*., 2017; Varma *et al*., 2018; Muchowska, Varma & Moran, 2019). In short, the most likely origin of glycolysis reflects a combination of the stability of glucose and the interconversion reactions mediated by metallo‐catalysis, driven by the high Fe^2+^ concentration in the early Earth environments (Aulakh, Varma & Ralser, 2022; Keller *et al*., 2014, 2016). Signatures of such ancient reactivity landscapes have been identified by computational ‘metabolic archaeology’ (Goldford *et al*., 2017; Meringer & Cleaves, 2017). Although sometimes overlooked, non‐enzymatic metabolic reactivity is still a part of modern metabolism (Keller, Piedrafita & Ralser, 2015) and becomes particularly important when cellular metabolism is thrown out of equilibrium by factors such as stress conditions or high temperatures. Notably, the ability of the pentose interconversion pathways to form a broad panel of life‐essential metabolic precursors indicates that pentose rather than hexose metabolism might have been at the core of the early metabolic structure (Lindner & Ralser, 2025). It is thus possible that glycolysis and gluconeogenesis branched from early pentose interconversion pathways, or were selected in parallel to them, in order to enable the storage of the equivalents of the central sugar phosphates.

### (3) Unwanted side reactions of glycolysis and metabolite repair

Potentially detrimental consequences of spontaneous (non‐enzymatic) reactions or enzymatic side reactions in metabolism are prevented by so‐called metabolite repair enzymes (Linster, Van Schaftingen & Hanson, 2013; Van Schaftingen *et al*., 2013; Hanson *et al*., 2016). In general, metabolic enzymes display high substrate and reaction specificity, maintaining carbon fluxes within well‐defined pathways that interlink to form metabolic networks. Enzyme specificity is never perfect, and virtually all enzymes can act, although often with much lower efficiency, on substrates other than their primary physiological ones (substrate promiscuity, Wohlfarter *et al*., 2022) or can catalyse non‐canonical reactions (reaction promiscuity). While enzyme promiscuity can serve as a starting point for the evolution of new enzyme functions (Khersonsky & Tawfik, 2010), enzymatic side activities usually form products that are useless or even toxic to cells. For example, due to the limited structural diversity among small metabolites, the average human metabolic enzyme is competitively inhibited by at least five unrelated cellular metabolites (Alam *et al*., 2017). Many promiscuously formed metabolites thus can interfere with metabolic function by inhibiting other metabolic reactions. Therefore, while enzyme promiscuity creates opportunities for metabolic evolution, it also necessitates the presence of metabolite repair enzymes that mitigate the effects of non‐canonical side products, ensuring the proper functioning of metabolic pathways that coexist in living cells. These repair enzymes are often widely conserved, highlighting their critical importance in all forms of life. In humans, the importance of metabolite repair enzymes is illustrated by the devastating disorders that stem from loss‐of‐function mutations in their corresponding genes (Veiga‐da‐Cunha, Van Schaftingen & Bommer, 2020). In fact, the concept of metabolite repair emerged from studies of the molecular cause of L‐2‐hydroxyglutaric aciduria, an inborn error of metabolism (Rzem *et al*., 2004, 2007; Rzem, Van Schaftingen & Veiga‐da‐Cunha, 2006). L‐2‐hydroxyglutarate (L2HG), the toxic metabolite accumulating in this disease, is formed by minor side activities of MDH and lactate dehydrogenase (LDH), whose main substrates are oxalacetate and pyruvate, respectively. Both enzymes can slowly reduce α‐ketoglutarate to L2HG, which, in the absence of the metabolite repair enzyme L2HG dehydrogenase, accumulates to levels that are high enough to trigger a severe neurometabolic disease (Van Schaftingen, Rzem & Veiga‐da‐Cunha, 2009). D2HG, the enantiomer of L2HG, accumulates in another disorder of metabolite repair caused by deficient D2HG dehydrogenase. In this case, while D2HG is formed by enzymatic side activities, it is also a product of the main physiological activity of certain enzymes, such as the transhydrogenase activity of the yeast Ser3 and Ser33 enzymes (Paczia *et al*., 2019). The latter initiate *de novo* serine synthesis from the glycolytic intermediate 3PG and concomitantly shuttle reducing equivalents from the cytosol to the mitochondrial respiratory chain *via* D2HG formation and the coupling with two additional enzymes (Dld3 and Dld1) (Becker‐Kettern *et al*., 2016).

Metabolite damage and repair are pervasive and affect all metabolic pathways, but many of the currently known examples cluster around glycolysis (Bommer, Van Schaftingen & Veiga‐da‐Cunha, 2020; Schmerling *et al*., 2022) and the citric acid cycle (Niehaus & Hillmann, 2020). This is consistent with the high metabolic flux carried by these pathways; slow but unwanted side reactions form unfavourable products in higher absolute amounts. Moreover, for less central metabolic pathways, as exemplified by polyamine metabolism, efflux remains a main mechanism to clear the cell of promiscuously formed metabolites (Olin‐Sandoval *et al*., 2019) and thus no specific repair enzyme is necessary in these cases. The metabolite repair concept emerged relatively recently, and it is likely that many metabolite repair enzymes still remain to be discovered (Ellens *et al*., 2017; de Crécy‐Lagard, Haas & Hanson, 2018; Griffith, Walvekar & Linster, 2021). Below, we describe a subset of the metabolite damage and repair reactions linked to glycolysis; for a more complete description, see the more comprehensive review of Bommer *et al*. (2020).

Hexokinases and ADP‐dependent glucokinase, catalysing the first step of glycolysis, can promiscuously phosphorylate 1,5‐anhydroglucitol (AG), a glucose analogue originating from food. The resulting non‐canonical AG‐6‐phosphate (AG6P) can in turn inhibit the first step of glycolysis. In healthy cells, AG6P is transported into the endoplasmic reticulum by the G6P transporter (G6PT), where it is then dephosphorylated by a repair phosphatase called G6PC3 (Veiga‐da‐Cunha *et al*., 2019). Deficiency of either G6PT or G6PC3 causes inherited disorders characterised by neutropenia: neutrophils, which have virtually no mitochondria and are particularly dependent on glycolysis for proper function, become dysfunctional and die because of AG6P accumulation. Lowering AG levels in the blood *via* administration of the kidney glucose reuptake inhibitor Empaglifozin rescues this neutropenia in G6PT‐ and G6PC3‐deficient patients; this treatment is now used in a majority of cases (Wortmann *et al*., 2020; Veiga‐da‐Cunha *et al*., 2023).

Probably one of the earliest known examples of metabolite damage and repair is the conversion of the glycolytic triose phosphate intermediates GAP and DHAP to the reactive dicarbonyl methylglyoxal. This reaction occurs spontaneously but can also be catalysed by a side activity of TPI (Richard, 1993). Methylglyoxal can form covalent adducts *via* reaction with amino groups in nucleic acids, proteins and metabolites, leading to the formation of advanced glycation end products (AGEs) (Scheckhuber, 2019). This can be efficiently prevented *via* the glyoxalase system, consisting of the highly conserved GLO1 and GLO2 enzymes that convert methylglyoxal to D‐lactate in a glutathione‐dependent manner (Sousa Silva *et al*., 2013).

GAPDH is involved in two known metabolite damage reactions. In addition to its physiological glycolytic substrate GAP, GAPDH can also act on the PPP intermediate E4P.This leads to the formation of 4‐phosphoerythronate, which is a strong modulator of the PPP *via* its inhibition on 6‐phosphogluconate dehydrogenase (Collard *et al*., 2016). This potential negative crosstalk between glycolysis and the PPP is prevented by the repair enzyme phosphoglycolate phosphatase (known as PGP in mammals and Pho13 in yeast), which efficiently dephosphorylates 4‐phosphoerythronate. PGP also eliminates a second byproduct of glycolysis, 2‐phospholactate, which is formed *via* promiscuous phosphorylation of lactate by PK. 2‐Phospholactate can slow glycolysis by inhibiting PFK‐2, the enzyme producing a potent stimulator of glycolysis (F2,6BP). Finally, PGP acts on a third potential glycolytic byproduct (also formed by PK *via* a side activity on glycolate), namely 2‐phosphoglycolate, which inhibits several other metabolic enzymes (Gerin *et al*., 2019). Interestingly, 4‐phosphoerythronate phosphatase activity has also been identified as a moonlighting activity of the *Bacillus subtilis* CpgA protein, primarily known for its checkpoint function in ribosome assembly (Sachla & Helmann, 2019). By maintaining low levels of 4‐phosphoerythronate, the metabolite repair activity of CpgA prevents inhibition of 6‐phosphogluconate dehydrogenase, which in turn prevents glycolytic blockage *via* 6‐phosphogluconate at the level of PGI.

In addition to its side reaction with E4P, GAPDH converts its cofactor NADH to a derivative known as NADHX (Rafter, Chaykin & Krebs, 1954), which is a hydroxylated form of NADH resulting from the addition of water to the 5–6 double bond in the nicotinamide ring (Oppenheimer & Kaplan, 1974). Later, researchers identified a conserved metabolite repair system, comprising an ATP‐dependent *S*‐NADHX dehydratase (NAXD) and an epimerase (NAXE), that catalyses the interconversion of the *S*‐ and *R*‐epimers of NADHX (Marbaix *et al*., 2011). Deficiency in either NAXE or NAXD leads to a lethal febrile‐induced encephalopathy usually starting in early childhood (Spiegel *et al*., 2016; Kremer *et al*., 2016; Van Bergen *et al*., 2019). The NAXE and NAXD enzymes also act on hydrated NADPH (NADPHX), which is even more prone to this type of damage than NADH (Marbaix *et al*., 2011).

Another damage cascade that can be initiated from a glycolytic intermediate was recently discovered using liquid chromatography–mass spectrometry (LC–MS)‐based metabolomic analyses of extracts derived from cell and animal models deficient in the Parkinson's disease‐linked protein PARK7 (also called DJ‐1) (Heremans *et al*., 2022). Here, the metabolic damage manifests in the accumulation of glycerate adducts of a number of metabolites containing free amino groups, including glutamate, glutathione, glycerophosphoethanolamine and lysine. These adducts were found to form *via* a reactive 1,3BPG derivative (thought to be cyclic 1,3‐phosphoglycerate) that avidly reacts with amino groups, while PARK7 maintained homeostasis by eliminating this derivative.

### (4) The thermodynamic basis of glycolysis

A derivative from the second law of thermodynamics is that a biochemical reaction only occurs if it is overall energetically favourable. The amount of energy required to form a compound is described by the ‘Gibbs formation energy’, while the driving force of chemical interconversions (e.g. anabolic or catabolic reactions of metabolism) is commonly referred to as ‘Gibbs free energy of reaction’, or just Gibbs energy (Held & Sadowski, 2016). A favourable reaction is thus one where the Gibbs energy is released, i.e. where the change in Gibbs energy (Δ*G*) is less than 0 (Chandel, 2021). The Δ*G* of a reaction is the difference between the Gibbs formation energies of reactants and products (Alberty, 1998). As a biochemical reactant (e.g. ATP) often consists of multiple species (e.g. ATP^4−^ and HATP^3−^), the Gibbs formation energy is dependent not only on the concentration of the reactant itself, but also on the biophysical environment, including the pH and the presence of other ions (Alberty, 2003). Thus, a reaction may change its direction depending on the concentration of reactants and products, the pH and the ionic strength of the environment. However, if a reaction has a large negative Δ*G*, it is possible that it cannot be reversed within the concentration range of metabolites in a cell (e.g. from μM to double‐digit mM concentrations) (Bennett *et al*., 2009).

Indeed, if we consider each reaction during steady‐state operation of a metabolic pathway (i.e. when there is a constant flux through the pathway and metabolite levels do not change), as being characterised by a momentary, steady‐state Δ*G* that depends on the concentration of reactants and products, pH and ionic strength, we can distinguish two cases. If a reaction operates close to equilibrium (Δ*G* of reaction is only minimally different from 0), it is indicated that there is sufficient enzyme activity to equilibrate the reaction compared to the up‐ and downstream reactions. This is the case for enzymes that have a high maximal activity compared to other enzymes in the pathway, so that they can convert substrates into products at the same rate by which they are produced and consumed by the up‐ and downstream reactions, respectively. Such reactions operating close to equilibrium are often reversible and changes in the activity or abundance of enzymes operating close to equilibrium have hardly any effect (Kümmel, Panke & Heinemann, 2006). By contrast, if a reaction has a highly negative, momentary Δ*G*, it means that this reaction operates far from equilibrium and is, under physiological conditions, not reversible. This happens, for instance, when an enzyme has a low maximal activity compared to the other enzymes in the pathway. In this case, the substrate accumulates and the product is swiftly converted into the next metabolite by the downstream reaction, thus leading to an imbalance between the concentration of substrate and product that pulls the reactions forward.

The Δ*G* values of glycolytic reactions in different organisms and under varying environmental conditions have been studied considering physiological metabolite levels (Park *et al*., 2019; Kümmel *et al*., 2006; Canelas *et al*., 2011; Vojinović & von Stockar, 2009; Maskow & von Stockar, 2005). While the enzymes of lower glycolysis (with the exception of PK) all operate very close to equilibrium, the reactions of HK, PFK in the upper glycolysis and PK operate far from equilibrium and are irreversible. PFK and PK have consistently been found to undergo regulation by small molecule effectors in species across kingdoms (Reznik *et al*., 2017). In mammalian cells, HK and PFK (but no reactions of lower glycolysis) and hexose and lactate transport were shown to exert flux control over glycolysis (Tanner *et al*., 2018).

Intriguingly, at physiological metabolite concentrations, the Δ*G* of the gluconeogenic enzyme FBPase indicates simultaneous occurrence with the glycolytic PFK reaction. If these two reactions indeed run at the same time, cyclic interconversions of F6P and F1,6BP would involve wasteful ATP hydrolysis. In order to limit energy losses through such a futile cycle, regulatory mechanisms, such as the inhibition of F1,6BPase by F2,6BP (Navas, Cerdán & Gancedo, 1993), are used to inhibit either the glycolytic or the gluconeogenic reaction (Locasale, 2018). However, one study (Zhao *et al*., 2012) investigated such futile cycles in the fungal species *Penicillium chrysogenum* and found substantial flux through FBPase during glycolytic mode (on the order of 50% of PFK flux).

The amount of Gibbs energy that a metabolic reaction dissipates over time can be estimated by multiplying Δ*G* by its flux. This calculation essentially quantifies the loss of the ability to do work and indicates how wasteful a reaction is. Values for Gibbs energy dissipation rates have been determined for *Escherichia coli* and *Saccharomyces cerevisiae* grown under different environmental conditions (Niebel, Leupold & Heinemann, 2019). Glycolysis contributes only about 5% to the overall cellular Gibbs energy dissipation rate in budding yeast growing with fully respiratory metabolism and low glycolytic rate. However, this contribution rises to 57% at high glycolytic rates and a fully aerobic fermentative metabolism, demonstrating how the same metabolic pathway can have very different contributions to the energetics of a cell under different conditions.

### (5) Metabolic control analysis of glycolysis

In almost all biochemical textbooks, one will find the statement that HK, PFK and PK are the rate‐limiting steps in glycolysis. This statement is loosely, and erroneously, based on the strong regulation of these enzymes and the high negative Δ*G* of their reactions. However, whereas perturbations of reactions that are close to equilibrium are unlikely to have large effects on steady‐state flux (as discussed in Section II.4), this does not automatically mean that reactions that are far from equilibrium are rate‐limiting steps (Noor *et al*., 2014; van Niekerk *et al*., 2023). In addition, regulation tends to decrease control of an enzyme (Sauro, 2017), i.e. if one makes a perturbation in the enzyme activity the regulation tends to negate the effect of the perturbation, leading to a smaller effect on flux. Lastly, the idea of multiple rate‐limiting steps indicates some sort of distribution of flux control, and it is unclear how to define a degree of control based on Δ*G* and the strength of allosteric regulation. Metabolic control analysis (MCA) was specifically developed to define the extent of control of reactions clearly and how that control can be interpreted in terms of enzyme sensitivities for substrates, products and regulators. For more in‐depth discussions of MCA, see (Hofmeyr, 2001; Kacser & Burns, 1995; Fell, 1997).

The control of flux has historically been somewhat loosely defined as the extent to which a reaction limits the flux. Here, we will use a more precise definition in terms of flux control coefficients (FCCs), as used in MCA. An FCC of an enzyme is defined as the percentage change in steady‐state flux upon a 1% change in the enzyme's activity. Experimentally, such perturbations in enzyme activity can be made *via* controlled overexpression of the enzyme using genetic techniques (Jensen, Michelsen & Westerhoff, 1993) or *via* inhibitor titrations (Groen *et al*., 1982; Kouril *et al*., 2023). This section will focus on experimental control analysis of glycolysis, while the application of mathematical models for MCA will be treated in later sections that focus on modelling of glycolysis.

MCA has applications beyond quantifying the effects of enzyme perturbation, as is evident from its summation and connectivity theorems, e.g. all FCC in a biochemical reaction network considered under steady‐state conditions add up to 1 (summation theorem). Thus, if an FCC of 0.4 is obtained for HK, then this step holds 40% of glycolytic flux control. FCCs are system properties, as they are dependent on the complete system, but they can be expressed in elasticities, which quantify the sensitivities of enzymes for effectors *via* connectivity theorems. Elasticities are local properties and can be determined on isolated enzymes removed from the system. Generally, enzymes with a high elasticity (e.g. enzymes close to equilibrium) will have little flux control.

The application of MCA to glycolysis has led to several insights. MCA requires that the system under study is well defined (including boundary conditions and mass balance for all metabolites) so that a steady state can be reached. In a strict sense, the boundary metabolites of glycolysis would be glucose and pyruvate, and it would be hard to study the pathway in intact organisms, as pyruvate is usually not excreted and cannot be kept constant. The boundary conditions are usually extended to external substrates and products, e.g. glucose, and ethanol and CO_2_ for yeast. While this pathway is redox‐neutral (i.e. balanced in terms of NADH production *via* GAPDH and NADH oxidation *via* alcohol dehydrogenase), it is not balanced in terms of ATP, and ATP consumption is part of the system as well.

While textbooks state that the kinases are the rate‐limiting steps in glycolysis, experimental evidence at hand paints a much more nuanced picture. Many studies refer to an early publication where control was distributed over HK and PFK (Torres *et al*., 1986), but in that study only three enzymes were analysed: HK, PGI and PFK. Since the FCC of a pathway adds up to 1, it is within expectations to find high control for the kinases (and not for the near‐equilibrium PGI reaction) in this three‐enzyme system, but such a system falls short of mimicking the *in vivo* situation. No increase in glycolytic flux was observed upon systematic overexpression of each of the individual enzymes for yeast glycolysis. Several mechanisms may explain such a situation: for instance, control could be distributed over many enzymes, rendering the flux control coefficient a condition dependent property. Initially, it was difficult to obtain experimental proof, and perturbing one enzyme at a time did not reflect this assumption (Schaaff, Heinisch & Zimmermann, 1989; Hauf, Zimmermann & Müller, 2000). However, supporting evidence for the dynamic distribution of flux control was obtained in functional genomic experiments. When studying yeast metabolism upon the deletion of protein kinases, it became evident that multiple enzymes of glycolysis undergo strong expression changes, thus indicating dynamic flux control redistribution. Factoring the dynamically changing FCCs in a MCA model rendered it predictive about measured glycolytic metabolite concentrations in the kinase knock‐outs (Zelezniak *et al*., 2018).

Other explanations remain plausible as well, and may contribute to metabolic regulation *in vivo*. For example, overexpression of glycolytic enzymes, which generally have high expression levels in fast‐fermenting organisms such as yeast and *Zymomonas mobilis*, might increase flux but cause a protein burden effect (Snoep *et al*., 1995). In the latter organism, for instance, an initial positive effect on flux upon overexpression of the first three glycolysis enzymes was negated upon further increasing their expression (Snoep *et al*., 1996). Another possible explanation is control mechanisms acting *in trans*, i.e. control mechanisms that lie outside the glycolytic pathway itself. Indeed, at the scale of the metabolic network, a broad set of metabolites are balanced. If glycolysis is seen as a pathway delivering ATP when needed, it could be that flux control lies in the demand (i.e. in ATP hydrolysis) (Westerhoff & Van Dam, 1987; Hofmeyr & Cornish‐Bowden, 2000). Indeed, for *Escherichia coli*, most of the flux control was found to reside in the demand for ATP (Koebmann *et al*., 2002a). This control by ATP demand depended on the organism and culture conditions – i.e. in *Lactococcus lactis*, no flux control was observed during growth, while a high flux control was observed in non‐growing cells (Koebmann *et al*., 2002b). For a multi‐functional pathway such as glycolysis, it is not surprising that flux control distribution differs between organisms and can shift upon changes in external conditions.

The distribution of flux control in cancer cells is of particular interest. Many but not all cancer cells use anaerobic metabolic reactions despite the presence of oxygen, a phenomenon often referred to as the ‘Warburg effect’ (discussed in more detail in Section V.1.*a*) (Warburg, 1956b). The increased glycolytic flux observed in these cells might alter flux control compared to normal cells and provide potential drug targets. Several approaches have been undertaken for MCA on cancer cells. Systematic upregulation of all individual glycolytic enzymes, including substrate and product transport steps, revealed high control for glucose transport, HK, PFK and lactate export in two cancer cell lines (Tanner *et al*., 2018). By contrast, through a study involving inhibitor titration, full control of glycolytic flux was measured for GAPDH (Shestov *et al*., 2014). Although GAPDH activities were calculated and not directly measured in this study, an effect on flux upon inhibition of GAPDH was observed. This was later confirmed in a follow‐up study on many cancer cell lines, showing a wide range of sensitivities for GAPDH inhibition (Liberti *et al*., 2017). Two more recent studies reported negligible flux control for GAPDH in cervical, gastric, colon, liver, lung and breast cancer cell lines (Zhu *et al*., 2021; Kouril *et al*., 2023). In both of these studies, significant decreases in glycolytic flux were observed only after GAPDH activities were reduced to 20% of uninhibited activity, which is well within the physiological decline of GAPDH activity in stress situations (Talwar *et al*., 2023; Ralser *et al*., 2009). In the triple‐negative breast cancer cell line used in (Kouril *et al*., 2023), a high flux control of 0.5 was measured for HK. Thus, whereas the high flux control of GAPDH measured in Shestov *et al*. (2014) could indicate a specific drug target in cancer cells, this might be highly dependent on the specific cancer cell line. The more traditional control distribution observed in Tanner *et al*. (2018) and Kouril *et al*. (2023) does not point to a specific cancer drug target in glycolysis. As aforementioned, the GAPDH redox switch supports cancer cell survival when they are exposed to endogenous oxidative stress (Talwar *et al*., 2023). The individual role of an enzyme's control over flux is thus likely also condition dependent.

### (6) Autonomous glycolytic oscillations

Under steady‐state conditions, glycolysis is a pathway that can show oscillatory dynamics (reviewed in Goldbeter, 1996), meaning that the concentrations of the glycolytic metabolites undergo periodic fluctuations, even under constant external conditions. Glycolysis in yeast was first shown to exhibit oscillatory behaviour in the 1960s, and it was found that these oscillations could be maintained for long periods of time in intact cells and cell extracts (Ghosh & Chance, 1964; Chance, Schoener & Elsaesser, 1965). In this section, we review the subsequent experimental studies on such oscillatory behaviour. Kinetic modelling of the oscillations is treated in Section IV.1.*a*.

Glycolytic oscillations in intact cells and cell extracts can be followed *via* online measurement of NADH fluorescence, but measurement of intermediate concentrations has also been used. Studied glycolytic intermediates showed the same frequency but different amplitudes and phases. Interestingly, yeast cell populations display synchronised oscillations, and acetaldehyde has been suggested to be the communicating agent between the cells (Richard *et al*., 1996). A good mechanistic understanding of the oscillations in yeast makes this an ideal system for mathematical modelling, but glycolytic oscillations have also been observed in many other cell types including pancreatic β‐cells (Chou, Berman & Ipp, 1992), heart cells (O'Rourke, Ramza & Marban, 1994), and cancer cells (e.g. Amemiya & Yamaguchi, 2022).

A balance between glucose influx and ATP hydrolysis is needed to obtain glycolytic oscillations – likely to keep ATP and AMP levels within critical boundaries for switching between inhibition and activation of PFK activity. Thus, yeast cells must be harvested at diauxic shift and starved for glucose to observe sustained oscillations in intact cells, and cell extracts must be supplied with a constant, low influx of glucose. Typical periods for glycolytic oscillations in intact yeast cells are around 1 min; in cell extracts, this can be 10‐fold longer, which can be related to the much lower protein concentrations in extracts compared to the cytosol in intact cells.

The feedback loops that are necessary for a system to show oscillatory behaviour could result from allosteric and competitive interactions that constitute the regulatory structure of glycolysis. Such inhibitory interactions are frequent in metabolism and often emerge due to the limited structural diversity between cellular metabolites (Alam *et al*., 2017). However, while these feedback loops can plausibly explain the oscillations observed in intact cells and cell extracts, they do not explain the phenomenon of synchronous oscillations in a cell population that emerge in yeast under nutrient starvation (Tu *et al*., 2005, 2007). The oscillations may provide a mechanism for cells to synchronise their metabolic activity – this could be particularly important for synchronisation of insulin production in pancreatic β‐cells or for muscle contraction, although it is unclear how it would benefit yeast.

If glycolytic oscillations allow cells to synchronise their metabolic activities, one could wonder whether isolated cells can still oscillate or whether a critical biomass is necessary – i.e. are these oscillations a quorum‐dependent population effect, or are they a set of individually oscillating cells that can synchronise? The dynamical quorum sensing position (De Monte *et al*., 2007) was supported by the inability to show oscillations in individual cells isolated from an oscillatory culture (Poulsen, Petersen & Olsen, 2007; Chandra, Buzi & Doyle, 2011). However, later direct detection of oscillations in isolated cells using a microfluidic system (Gustavsson *et al*., 2012), and the observation of non‐synchronised but oscillatory cells using fluorescent microscopy (Weber *et al*., 2012; Weber, Zuschratter & Hauser, 2020) have shifted the balance back to the latter position, that synchronisation can be achieved also partially within a population. This behaviour appears to follow a mathematical Kuramoto model of oscillation (Strogatz, 2000).

When measuring the average signal from a population, one is limited to studying synchronised cultures (since, in non‐synchronised cultures, the oscillatory signals from individual cells cancel each other out), whereas single‐cell measurements allow observation of individually oscillating but not necessarily synchronised cells. Classic phase response curves for isolated cells revealed that cells adapt their phase towards synchronisation upon perturbation with acetaldehyde (van Niekerk *et al*., 2019), confirming this as a possible mechanism for synchronisation. A similar mechanism seems to underlie synchronisation waves and community formation in diffusion‐limited microfluidic systems (Mojica‐Benavides *et al*., 2021).

Thus, while the benefit of glycolytic oscillations for isolated cells is not clear, they might play important roles in coordinating metabolic activity in organs (e.g. heart and pancreatic β‐cells) and in community formation in unicellular organisms.

## III. QUANTIFYING GLYCOLYSIS

### (1) From indirect assessment of glycolytic activity to its quantification using LC–MS, biosensors and NMR

Even before the reaction sequence of the glycolytic EMP pathway was mapped, its physiological importance was already apparent from the first examples of metabolic regulation affecting glycolytic activity: the Pasteur, Crabtree and Warburg effects (see Section V.1.*a*). These discoveries were driven by advances in volumetric, gravimetric and colorimetric methods for estimating glycolytic activity based on the extracellular levels of glycolysis substrates and end products (e.g. glucose and lactate) in muscle tissue (Clausen, 1922; Evans, 1925). The principle underlying these advances is that a relative increase in glycolytic activity leads to more glucose consumption and production of respective electron acceptors such as lactate (Tanner *et al*., 2018). By contrast, parameters reflecting respiration, such as O_2_ consumption and CO_2_ production, decrease (Mookerjee & Brand, 2015). While providing only indirect and relative measures of glycolytic activity, the advantage of these parameters is the ease, non‐invasiveness and simplicity of the options available for assessing them (TeSlaa & Teitell, 2014). For instance, glucose and fermentation products can be quantified in biological fluids and culture media using colorimetric assays that couple their enzymatic conversion to compounds such as NAD(P)H, which can be monitored by spectrophotometry (TeSlaa & Teitell, 2014).

To assess glucose uptake in cells or tissues, non‐metabolisable glucose analogues such as 2‐deoxyglucose (2‐DG) can be quantified by using fluorescent (Speizer, Haugland & Kutchai, 1985) or radioactive (Sokoloff *et al*., 1977) labels or by following their enzymatic conversion *via* photometric or luminescent readouts (Yoshioka *et al*., 1996). Each specific detection method and glucose analogue brings various pitfalls and special considerations. Most importantly, several of the metabolite analogues influence the pathways they aim to report. For instance, 2‐DG itself acts as a glycolytic inhibitor and has pleiotropic effects on other metabolic pathways, in particular the PPP (Ralser *et al*., 2008; reviewed in Yamamoto *et al*., 2015).

The relative contributions of glycolytic and oxidative activity can also be determined by measurements that rely on the extracellular acidification rate (ECAR) and oxygen consumption rate (OCR) in cultured cells (Mookerjee & Brand, 2015; Zhang *et al*., 2012; TeSlaa & Teitell, 2014). The ECAR indicates fermentation under certain conditions where the catabolic breakdown of glucose to lactate leads to concomitant translocation of protons and extracellular acidification. However, other activities that acidify the extracellular environment (e.g. carbonic acid production *via* respiration) must be accounted for. Quantification of the OCR as an indicator of respiratory activity allows correction for its impact on the ECAR, and the glycolytic rate can hence be estimated. These measurements can be further refined by chemical inhibitors of glycolysis or respiration. Notably, such inhibitor‐based glycolytic stress tests also allow inferences about the glycolytic reserve, i.e. the available but temporarily unused flux capacity of glycolysis. There are several methods for determining ECAR and OCR in cell cultures, including dedicated analysers that employ O_2_‐sensitive dyes and pH probes to assess oxygen levels and pH in parallel in microtiter format (Zhang & Zhang, 2019; reviewed in Zhang *et al*., 2012; Mookerjee, Nicholls & Brand, 2016; TeSlaa & Teitell, 2014).

Direct measurement of the abundance and turnover of glycolytic intermediates, although more laborious, is often required for inferring glycolytic flux. This includes determining concentration changes of key metabolites such as F1,6BP across organisms, whose levels have been shown to correlate with the glycolytic rate (Jang, Chen & Rabinowitz, 2018; Christen & Sauer, 2011; Kochanowski *et al*., 2013). Intracellular levels of F1,6BP can be followed *via* biosensors, which have the advantage of working *in vivo* and offer the opportunity of identifying subpopulations with different glycolytic flux behaviours. For instance, such a biosensor was developed for *Saccharomyces cerevisiae* based on a transcription factor from *Bacillus subtilis* (Monteiro *et al*., 2019). Alternatively, an *in vitro* selected aptamer for F1,6BP was engineered into a riboswitch that generates ratiometric fluorescent readout of glycolytic flux (Ortega *et al*., 2021), and this system was used successfully for monitoring yeast metabolism during industrial processes (Torello Pianale, Rugbjerg & Olsson, 2021). F1,6BP‐based sensors were also developed for mammalian cells (Geraci *et al*., 2022; Koberstein *et al*., 2022).

Finally, for obtaining a more comprehensive picture of the metabolic activities within glycolysis, LC or gas chromatography (GC) mass spectrometry and nuclear magnetic resonance (NMR) can quantify multiple intermediate metabolites. These can be combined with stable isotopic tracers, and measuring their incorporation into glycolytic intermediates in steady state can further help to elucidate changes in glycolytic activity. For quantification of actual flux, dynamic labelling experiments and measurements of the levels of glycolytic intermediates are required (Wittmann, 2002; Lehmann, 2017). Both metabolomics and tracing experiments typically rely on parallel identification and analysis of glycolytic intermediates by chromatographic separation followed by MS‐based detection. For chromatography, a range of methods including GC, capillary electrophoresis and LC are used. Among these, GC and LC prevail, as they can retain and separate the highly polar sugar phosphates with high sensitivity (Büscher *et al*., 2009). LC, in particular, stands out for its advantages: it requires no chemical derivatisation, allowing for simpler and faster preparation. Moreover, LC enables high throughput using high‐performance LC (HPLC) and can achieve even greater sensitivity using, e.g. nano‐flow LC (Kiefer, Portais & Vorholt, 2008). A range of LC methods, including ion‐pairing reverse phase (Mathon, Barding & Larive, 2017), ion exchange (Schwaiger *et al*., 2017) and hydrophilic interaction chromatography (HILIC) and HILIC with zwitterionic functionalities (ZIC‐HILIC) have been developed (Teleki, Sánchez‐Kopper & Takors, 2015; Mathon *et al*., 2017; Jorge & António, 2018). Chromatography is usually connected online via electrospray ionisation to a mass spectrometer as a detector for quantification, identification, and to enable separation of mass isotopologues originating from tracer experiments (Wittmann, 2002; Kasarla *et al*., 2024).

For studying glycolysis, NMR has the advantages of being non‐invasive and non‐destructive, and in some instances can be performed *in vivo* without the need for metabolite extraction (den Hollander *et al*., 1979). First applications of NMR in the glycolysis field trace back to the 1970s and pioneering studies include one analysis in *Saccharomyces cerevisiae* (den Hollander *et al*., 1979) and another in *Escherichia coli* (Ugurbil *et al*., 1978) using ^13^C and ^31^P NMR, respectively; these provided insights into the kinetics of key enzymes (FBA and TPI) in yeast and the relationship between pH levels and ATP production rates in *Escherichia coli*. NMR spectroscopy has been used to study glycolysis in various clinical settings, e.g. in patients with muscular myopathies caused by enzyme deficiencies (Duboc *et al*., 1987); in ischemic myocardial contracture, linking glycolytic ATP production with the onset of contracture (Kingsley *et al*., 1991); for analysing metabolic changes in the brain following acute stroke, detecting continued anaerobic glycolysis (Bruhn *et al*., 1989); in cancer, studying the biochemical network that allows tumour cells to synthesise essential compounds *via* glycolysis and the citric acid cycle (DeBerardinis *et al*., 2007); and in malaria patients treated with various antimalarial agents, studying the viability of *Plasmodium* parasites as a function of glycolytic activity (Shivapurkar *et al*., 2018). NMR spectroscopy has also been used in biotechnology research to characterise the kinetics of glycolysis in *Lactobacillus lactis*, of relevance for food fermentation (Neves *et al*., 1999).

The introduction of hyperpolarised tracers has dramatically improved detection sensitivities and capabilities of NMR (in a medical context, magnetic resonance imaging, MRI) (Golman *et al*., 2003; Ardenkjaer‐Larsen *et al*., 2003; Laustsen *et al*., 2013). ^13^C probes are used most frequently, but other nuclei such as ^15^N and ^31^P are also available. (1‐^13^C)pyruvate is the most commonly used tracer, as the conversion of (1‐^13^C)pyruvate to either (1‐^13^C)lactate or (^13^C)bicarbonate indicates whether fermentation or oxidative phosphorylation has occurred. The alteration in chemical shift that accompanies these conversions can be detected with very high temporal resolution *via* hyperpolarised MRI, e.g. the increased accumulation of lactate – indicative of the Warburg effect, as discussed in Section V.1.*a*. While positron emission tomography (PET) detects glucose uptake, hyperpolarised (1‐^13^C)pyruvate MRI completes the picture by determining the subsequent fate of this glucose (Gutte *et al*., 2015). An example in the context of cardiac metabolism was provided by (Rider *et al*., 2020) demonstrating the complementarity of these two technologies and their value for biomedical research. Glucose itself can also be hyperpolarised and detected with MRI, which has delivered insights into tumour glycolysis (Rodrigues *et al*., 2014).

### (2) Quantifying activity and regulation of glycolysis through the lens of its enzymes

Another option for studying glycolysis and its regulation is the analysis of glycolytic enzymes. This includes quantification of their activities, abundance, post‐translational modifications (PTMs) and the formation of isoforms. These analytics are simplified by the abundance of glycolytic enzymes in the cell (Carroll *et al*., 2011; Fraenkel, 2003), which facilitates measurement of enzyme activity in cell extracts (e.g. by colorimetric means) (TeSlaa & Teitell, 2014). However, this approach has limited throughput and may not reflect *in vivo* rates (Teusink *et al*., 2000). A few examples of assays for high‐throughput measurement of glycolytic enzyme activity can be found in the cancer field (Cho *et al*., 2018) and in crop science (Gibon *et al*., 2004).

With proteomics becoming quantitative and covering major PTMs such as phosphorylation (Oliveira & Sauer, 2012), direct quantification of the intracellular concentration of glycolytic enzymes is increasingly feasible using proteomics. Despite the multifactorial relationship between enzyme levels and metabolic phenotypes, recent advances show that if considered together, the levels of multiple glycolytic enzymes are predictive of the concentration of glycolytic intermediates (Zelezniak *et al*., 2018). Combined with knowledge of PTMs that contribute to metabolic regulation, proteomics experiments yield valuable insights into changes in metabolism (Tan *et al*., 2017; Sacco *et al*., 2019).

In summary, evaluation of changes in glycolytic activity requires a multifaceted approach, including measurements of intermediates and products of glycolysis, enzymatic assays, proteomic analyses, quantitative metabolomics and metabolic flux measurements. The utilisation of LC–MS stands out in this context, offering high sensitivity and specificity in quantifying glycolytic intermediates and products, its enzymes, and their modifications. Additionally, biosensors allow monitoring of glycolytic flux down to the single‐cell level, and NMR is the method of choice to study dysregulations of glycolysis *in vivo* and their contribution to diseases.

## IV. METHODS FOR STUDYING GLYCOLYTIC DYNAMICS AND REGULATION

### (1) Computational modelling

As glycolytic enzymes are tightly regulated and are responsive to a series of feedback loops and metabolite–protein interactions, the pathway shows complex behaviour *in vitro* and *in vivo*. Several modelling approaches have thus far been applied in order to capture and predict its metabolic properties. For example, kinetic modelling approaches that integrate information on enzyme kinetics to study pathway dynamics are especially applicable to a relatively short pathway like glycolysis. Kinetic modelling has a particularly rich history in *Saccharomyces cerevisiae*, not only because of the commercial importance of this organism for industrial fermentation, but also because modelling needs large sources of data, which are generally available for this organism (reviewed in Lao‐Martil *et al*., 2022). Another approach is to use genome‐scale metabolic models that are based on reaction stoichiometry. These ‘constraint‐based models’ map gene‐to‐protein‐to‐reaction relationships using bioinformatics tools, based on genomic and physiological data (reviewed in Bordbar *et al*., 2014). Such models attempt to provide a global picture of the metabolic potential of a given organism or cell type – as such, they are particularly useful for studying the impact of glycolysis or its variants in the context of the overall metabolic network. More recently, machine learning (ML)‐based approaches have become increasingly popular, and allow prediction of metabolite concentration changes and monitoring of basic metabolic properties such as catalytic properties of enzymes or the likelihood of interaction with small molecules (Zelezniak *et al*., 2018; Li *et al*., 2022a; Kroll *et al*., 2021; Hackett *et al*., 2016).

#### (a) Kinetic modelling of glycolysis

A kinetic model is a tool to simulate the dynamic behaviour of a metabolic pathway. The rates of individual reactions are explicitly described as a function of the concentrations of the reactants and regulators. Challenges arise due to the possible array of kinetic parameters of the enzymes involved, which need to be determined beforehand. Specifically in the case of glycolysis, sophisticated analyses are necessary to understand complex dynamical behaviours such as oscillations (see Section II.6) or bistabilities (see below in this section). However, what the models yield is a much deeper mechanistic understanding of the regulation of the pathway and thereby the ability to predict the outcome of perturbations, e.g. caused by drugs or metabolic engineering.

The most common approach to kinetic modelling is to use mass balances for each metabolite in the pathway, taking into account all the reactions that affect its concentration. The model therefore consists of the set of balances for all metabolites in the pathway. When the kinetic parameters needed to specify the reaction rates [e.g. velocity constant (*k*_cat_), Michaelis–Menten constant (*K*_m_), allosteric regulation binding constants of activation (*K*_*A*_) or inhibition (*K*_*I*_) for enzymes regulated by allosteric effectors, initial metabolite concentration] are provided, the balance equations can be solved numerically. The solution of a set of such equations, i.e. the output of the model, returns the concentrations of metabolites as a function of time – and thus the rates of the reactions.

Typically, upon starting a time simulation from some initial condition, the system will show some dynamic behaviour called the ‘trajectory’ before settling into a steady state where all metabolites are balanced, meaning that the rates of producing reactions equal the rate of consuming reactions. In this state, all the balances are zero and the metabolite levels remain constant in time, while a constant flux runs through the pathway. Depending on the research question, the initial trajectory may be ignored and only the steady states are analysed. In biotechnology, for example, the steady‐state fermentation rate (hence, the glycolytic flux) may be the objective, and researchers may want to understand how these chemical flows change upon metabolic engineering. For such purposes, the popular approach of flux balance analysis is the method of choice. In contrast to kinetic modelling, flux balance analysis allows the investigation of whole genome‐scale metabolic networks because it deals only with steady state and ignores the complexity that comes with dynamic changes and looking at kinetics (Orth, Thiele & Palsson, 2010; Santos, Boele & Teusink, 2011). Kinetic modelling, on the other hand, excels at elucidating the fine‐tuned regulation of metabolic pathways and their dynamics over time.

The first step, particularly relevant for central pathways such as glycolysis, is defining the boundaries of the system (Liebermeister, Baur & Klipp, 2005). For example, the ATP produced by glycolysis is consumed by many different reactions. It is also important to model correctly all the branches of glycolysis as this has a large impact on the models' outcome (Teusink *et al*., 2000; Lao‐Martil *et al*., 2023). Then, many kinetic parameters will be uncertain or unknown. Trying to fit all of the glycolytic parameters to dynamic, information‐rich data sets has been attempted with modest success (Levering *et al*., 2012; Lao‐Martil *et al*., 2023), and some *in vitro* enzyme kinetic data are often required as an initial estimate. However, only a few parameters will have a substantial impact on the behaviour of the model (for a representative example in cancer metabolism, see Kelly *et al*., 2018). The number of parameters in a dynamic model can grow rapidly and requires a high number of simulations.

Despite the complexity of the task, kinetic models of glycolysis have been described for many organisms and cell types and can be shared using standardised formats (Hucka *et al*., 2003; Olivier, Swat & Moné, 2016) in databases such as *BioModels* (Malik‐Sheriff *et al*., 2020) and *JWS Online* (Peters *et al*., 2017).

In particular, there are a number of models for *Saccharomyces cerevisiae* in which glycolysis has been extensively studied (reviewed in Lao‐Martil *et al*., 2022). Two models from the early 2000s (Teusink *et al*., 2000; Hynne, Danø & Sørensen, 2001) were later widely used for the development of systems biology methods. Notably, one of the models (Teusink *et al*., 2000) led to the discovery of glycolytic bistability, which is the coexistence of two stable states with very different physiological consequences. One state corresponds to a global steady state with normal growth on glucose; the other, instead, is an imbalanced state in which the preparatory phase of glycolysis outpaces the payoff phase. This imbalance is maintained by the autocatalytic stoichiometry of glycolysis, resulting in very low phosphate and ATP levels and a continuous accumulation of F1,6BP. These two states were shown to correspond to two distinct subpopulations of yeast cells (Van Heerden *et al*., 2014). This discovery explained enigmatic yeast cell phenotypes that had been observed many years previously. For example, addition of low concentrations of glucose to mutants in trehalose metabolism in a galactose excess medium led to apparent growth inhibition (Neves *et al*., 1995), but in reality pushes a subpopulation into the non‐growing imbalanced state (Van Heerden *et al*., 2014). The role of phosphate in the behaviour of glycolysis was also emphasised in a comparative study between two lactic acid bacteria, *Lactococcus lactis* and *Streptococcus pyogenes*, even though imbalanced states were not found in these organisms (Levering *et al*., 2012).

Overall, applications of kinetic models of glycolysis are manifold. As they allow the simulation of a pathway's dynamics over time, adaptation of the Teusink model explained glycolytic oscillations (du Preez *et al*., 2012). Kinetic models of glycolytic oscillations have been recently reviewed by van Niekerk *et al*. (2024). Moreover, kinetic models of glycolysis can predict metabolic rates and metabolite concentrations in the steady state; e.g. explaining metabolic differences between healthy and diseased states (Schuster & Holzhütter, 1995) or between different physiological conditions (Schuster, Holzhütter & Jacobasch, 1988). Finally, sensitivity analysis can identify the key parameters having an impact on pathway behaviour (Fell, 1992), allowing ‘network‐based drug design’ that leverages on differences in parameter sensitivity between host and pathogen to select promising drug targets (Gerber *et al*., 2008; Saavedra *et al*., 2019). This feature was exploited in parasitology to suggest treatment targets against *Trypanosoma brucei*, the parasite causing African sleeping sickness, for example glucose transporters or glycolysis enzymes such as GAPDH (Bakker *et al*., 1997, 1999, Haanstra *et al*., 2017). Other parasites are being modelled in similar ways, such as *Trypanosoma cruzi* (reviewed in Saavedra *et al*., 2019) and the malaria parasite *Plasmodium falciparum* (Penkler *et al*., 2015).

Glycolysis models have also been widely applied to human cells, particularly skeletal muscle (Schmitz *et al*., 2010, 2013) and cancer cells (Maier *et al*., 2010; Marín‐Hernández *et al*., 2011; Shestov *et al*., 2014; Liberti *et al*., 2017; Li *et al*., 2020b). In skeletal muscles, maintaining ATP homeostasis requires fast buffering systems as well as rapid activation and shutdown of metabolic activities upon sudden changes in ATP demand. Kinetic modelling was used to interpret high‐resolution dynamic data obtained by *in vivo* NMR studies in whole muscles (Schmitz *et al*., 2013), and led to the hypothesis that binding of PFK to the cytoskeleton may be responsible for the fast changes in activity (Marinho‐Carvalho *et al*., 2009; Schmitz *et al*., 2010).

In cancer, kinetic models have been developed to investigate the increased glycolytic flux known as the Warburg effect (see Section V.1.*a*). In liver cancer, incorporation of a time series of metabolite concentrations into a dynamic model (Maier *et al*., 2010) revealed GAPDH to be a potential drug target. This notion is supported by modelling and inhibitor studies (Shestov *et al*., 2014; Liberti *et al*., 2017). GAPDH was recently independently targeted with a new inhibitor that appears to have mild selectivity towards breast cancer cells (Li *et al*., 2020b). Additionally, a glycolytic model of rat hepatoma indicated HK, PGI and glucose transport as main controlling steps and suggested them as drug targets (Marín‐Hernández *et al*., 2011).

It is noteworthy mentioning that some models have not only consolidated our understanding of glycolysis, but revealed new concepts. These include (*i*) the model of Van Heerden *et al*. (2014), that elucidated the ‘start up’ problem of glycolysis, or (*ii*) the work of Zelezniak *et al*. (2018), that illustrated that metabolic control jumps between different enzymes under different conditions, and that this explains the low correlation of enzyme abundance with flux.

#### (b) Constraint‐based modelling of glycolysis

Constraint‐based metabolic modelling links the metabolic phenotype – comprising reaction rates (i.e. fluxes), enzyme abundances and metabolite concentrations – to physiological phenotypes, including specific growth rates, biomass yields and exchanges with the environment. This modelling framework forgoes mathematical descriptions of enzyme kinetics that specify how enzyme and metabolite concentrations determine reaction fluxes; instead, it imposes constraints on the metabolic phenotype capturing basic physicochemical principles, such as mass balance and thermodynamic feasibility. As a result, constraint‐based modelling is based on the key assumption that the modelled metabolic network operates at steady state, whereby the rates of consumption and production of each internal metabolite are balanced. To link the metabolic with a physiological phenotype, constraint‐based modelling also assumes that the modelled metabolic network optimises a physiological outcome (e.g. growth, energy consumption). The physiological outcome can be expressed either (*i*) as the rate of an artificial reaction describing the draining of metabolic precursors needed for growth or (*ii*) as the rate of an exchange reaction denoting the network's input/output relations with the environment. Taken together, these assumptions and constraints are the basis of flux balance analysis – the seminal representative of the constraint‐based modelling framework (Orth *et al*., 2010; Bordbar *et al*., 2014).

Constraint‐based modelling allows the study of glycolysis alone or integrated into metabolic models of organelles (Ramakrishna *et al*., 2001) or of entire cells and organisms (Bordbar *et al*., 2014; Yizhak *et al*., 2015). For instance, one of the earliest constraint‐based models of *Escherichia coli* metabolism included a partial two‐step representation of glycolysis, transforming the input triose phosphate into PEP and in turn into pyruvate (Majewski & Domach, 1990; Reed & Palsson, 2003). To build a mass‐balanced model of glycolysis, the set of eleven reactions that transform D‐glucose to pyruvate and lactate must be augmented with additional exchange reactions (i.e. import/export of glucose, cofactors, water, protons and phosphate) and reactions from AMP metabolism (Palsson, 2011). In such a pathway‐centric model, glycolysis can be linked to growth by considering additional side reactions that drain glycolytic intermediates for production of biomass precursors (e.g. serine) (Dai *et al*., 2016). Since glycolytic intermediates participate in other metabolic pathways, understanding the effects of glycolysis on the metabolic and physiological phenotypes necessitates studying this pathway's function in the context of large‐scale metabolic models.

Constraint‐based analysis of large‐scale metabolic models has been used to address three questions related to glycolysis: (*i*) do steady‐state fluxes in a genome‐scale metabolic network allow reconstitution of the textbook glycolytic pathway? (*ii*) Which constraints provide an explanation for the Warburg effect, the Crabtree effect (see Section V.1.*a*) and overflow metabolism in general (i.e. when cells undergo aerobic fermentation rather than respiration and excrete fermentation metabolites in the process, reviewed in Section V.1) as counterintuitive phenomena? (*iii*) Can integration of ‐omics data clarify the kinetic properties of enzymes involved in glycolysis?

As a central metabolic pathway across organisms from all kingdoms of life, it is unsurprising that glycolysis is included in virtually all large‐scale metabolic models (Norsigian *et al*., 2020; Lachance *et al*., 2021; Masid, Ataman & Hatzimanikatis, 2020; Hädicke & Klamt, 2017; Küken *et al*., 2021; Smith *et al*., 2017). It is therefore important to determine whether the relationship between the fluxes of the 11 glycolytic reactions in genome‐scale metabolic networks can recover the textbook glycolytic pathway. To this end, early application of flux balance analysis to large‐scale models of *Escherichia coli* metabolism demonstrated that some of the glycolytic reactions and intermediates are involved in the high‐flux metabolic backbone across different environmental conditions (Almaas *et al*., 2004). Additionally, recent analyses have demonstrated that steady‐state flux distributions from genome‐scale metabolic models of *Escherichia coli* and *Saccharomyces cerevisiae* can partly recover the structure of this pathway under scenarios with and without imposing optimisation of a growth rate (Küken, Langary & Nikoloski, 2022). Therefore, the flux phenotype of glycolysis seems to be an emerging phenotype from the function of the entire metabolic network.

Constraint‐based modelling of metabolism has also provided important insights into the link between physiological outcomes and glycolysis. For example, early constraint‐based modelling analysis of the Warburg effect was based on a highly reduced model of cell metabolism in which the glucose uptake flux is partitioned into the flux of aerobic glycolysis, oxidative phosphorylation and the production of precursor metabolites needed for growth (Vazquez & Oltvai, 2011; Vazquez *et al*., 2010). More recently, a simplified flux balance model of glycolysis (Dai *et al*., 2016) was employed to determine redox‐balancing conditions necessary for the Warburg effect and its correlation with proliferation rate. Importantly, this model also considered constraints on volume occupied by the enzymes supporting metabolic fluxes, which are incorporated in flux balance analysis with molecular crowding (FBAwMC) (Beg *et al*., 2007). The key prediction from FBAwMC is that, at low glucose uptake rates, ATP is entirely produced by the oxidative phosphorylation pathway and there is no lactate production (and excretion); this continues with increasing glucose uptake rate up to a threshold value above which further increase in ATP production through oxidative phosphorylation is limiting. However, additional glucose uptake may be diverted towards aerobic glycolysis, resulting in a linear increase in lactate production even in presence of oxygen. A similar approach, incorporating enzymatic constraints that bound fluxes in terms of enzyme turnover numbers and abundances, was used in a study that considered large‐scale models of human metabolism (Shlomi *et al*., 2011). Further refinement of FBAwMC resulted in the imposition of membrane occupancy constraints that provide another possible explanation for the Warburg effect (Zhuang, Vemuri & Mahadevan, 2011). Trade‐offs in protein allocation (Hashemi, Laitinen & Nikoloski, 2023), considered in constraint‐based modelling by integration of turnover numbers, have been used to offer an explanation of the Crabtree effect in *Saccharomyces cerevisiae* (Oftadeh *et al*., 2021; Sánchez *et al*., 2017) and the bacterial Crabtree effect in *Escherichia coli* (Basan *et al*., 2015). These phenomena are discussed in more detail in Section V.1.*a*.

Lastly, constraint‐based modelling has provided important insights into the *in vivo* turnover numbers of enzymes (Ferreira, da Silveira & Nikoloski, 2024). Data on fluxes and enzyme abundances for a reaction under different conditions allow us to calculate apparent catalytic rates (Valgepea *et al*., 2013). The maximum apparent catalytic rates for an enzyme over these different conditions can then be used as a proxy for the *in vivo* turnover number of the enzyme. For instance, such an approach applied with fluxomics and quantitative proteomics data from *Escherichia coli* showed that the proxies for *in vivo* turnover numbers matched the *in vitro* values only for GAPDH; conversely, the *in vitro* turnover numbers were underestimated for PFK and overestimated for PGI, TPI and enolase (Davidi *et al*., 2016). A similar discrepancy was observed when imposing the constraint of non‐idle enzymes (Xu, Razaghi‐Moghadam & Nikoloski, 2021). As a result, usage of metabolic phenotypes from constraint‐based modelling with quantitative proteomics data paves the way for obtaining reliable estimates of kinetic parameters that can improve kinetic models of glycolysis.

### (2) Machine learning applications in studies of glycolysis

Constraint‐based modelling allows us to predict metabolic phenotype and investigate its association with glycolysis; it does not, however, provide information about how metabolites affect reaction fluxes by interacting with glycolytic enzymes. Moreover, for many pathways from peripheral to central metabolism, and for most species that are not common model organisms, there is only limited experimental enzymological data available, creating the risk of overfitting any model when estimating parameters. This challenge can be addressed by applying ML frameworks to predict metabolite–protein interactions and their properties. Specifically, applying ML to glycolysis addresses three problems related to metabolite–protein interactions: (*i*) determining whether or not a given metabolite interacts with a protein; (*ii*) quantifying the binding affinity of the metabolite to the protein and the subsequent effects on fluxes; and (*iii*) specifying the metabolite–protein interaction sites.

Addressing these problems requires accurate representations of the input protein–metabolite pairs. This is achieved by: (*i*) using known protein features, such as global features of the protein sequence or features of the amino acids, and known compound features, including global chemical properties and/or fingerprinting codes describing substructures in the metabolite (Lim *et al*., 2021; Xu *et al*., 2020); or (*ii*) relying on protein and compound representations produced by deep learning, for instance by graph neural networks (Huang *et al*., 2021; Zhou *et al*., 2021).

Supervised ML of metabolite–protein interactions also requires access both to confirmed interactions, gathered in databases like *BioSnap* (Leskovec & Sosič, 2016) and *STITCH* (Kuhn *et al*., 2008), and to data on non‐interacting metabolite–protein pairs, obtained by experimental chemoproteomic workflows (Piazza *et al*., 2018; Veyel *et al*., 2018). The lack of reliable gold standard data for non‐interacting metabolite–protein pairs has resulted in adoption of several strategies to emulate such pairs, from random selection (i.e. choosing random metabolite–protein pairs and assuming they do not interact) to learning of ML models (i.e. training models to predict or identify non‐interacting pairs based on patterns in the data). To increase interpretability of these models further, particularly with respect to metabolite–protein interaction sites, different attention mechanisms have already been applied in training deep learning models for prediction of metabolite–protein interactions (Li *et al*., 2020a; Agyemang *et al*., 2020; Campana & Nikoloski, 2023). Lastly, to assess adequately the generalisability of the model, one must assess its performance in a double‐blind setting in which the model is tested on metabolites and proteins that have not been seen in the training of the model; so far, this has been performed in only a few studies (Campana & Nikoloski, 2023; Li *et al*., 2020a). Models with attention mechanisms are specifically trained to focus on the parts of the data (e.g. certain features of proteins or metabolites) that are most relevant and seem to exhibit good generalisability even to proteins and metabolites from organisms whose data were not used in model training (Campana & Nikoloski, 2023). While these approaches pave the way towards discovery of yet‐unknown regulators of glycolytic enzymes, focused studies are still rare.

A ML model was recently used to attempt the prediction of *K*_m_ from different metabolite and enzyme features for documented substrate–enzyme combinations (Kroll *et al*., 2021); the resulting model explained around 40% of the variance in the test set, indicating the possibility of further advances. In this direction, a data‐fitting approach to predict the likelihood of metabolites acting as enzyme activators or inhibitors was recently developed (Hackett *et al*., 2016). This approach was based on fitting and discriminating models of increasing complexity based on reversible Michaelis–Menten kinetics with random binding, meaning that the models (*i*) accounted for both the forward reaction (i.e. enzyme binding to the substrate to form a product) and the reverse reaction (i.e. product reverting back to substrate) and (*ii*) did not set a strict order to how enzyme and substrate bind, thus making the model more versatile. Such models were then applied with metabolomic and proteomic data along with fluxomic predictions from constraint‐based modelling in *Saccharomyces cerevisiae* under different growth conditions. The results provided a ranking of particular metabolic steps to be regulated by at least one metabolite (e.g. one of the findings suggested that PK is inhibited by citrate).

Finally, a deep learning approach enabled the genome‐scale prediction of *k*_*cat*_ from substrate structural features and protein sequences. The newly predicted *k*_*cat*_ improved existing metabolic models, and a trend of *k*_*cat*_ distribution discriminating between Crabtree‐positive and negative yeasts emerged. Remarkably, Crabtree‐positive species showed higher predicted *k*_*cat*_ values of pyruvate kinase (in agreement with a higher rate of glycolysis and fermentation), while Crabtree‐negative species exhibited higher predicted *k*_*cat*_ values of citrate synthase (which marks the entrance into the citric acid cycle and favours respiratory energy metabolism) (Li *et al*., 2022a). These ML approaches, in combination with predictions of metabolite–protein interactions, can provide valuable input for the generation of kinetic models of glycolysis.

Another key application of ML‐based approaches is to decipher the regulatory landscape surrounding metabolism. For example, because the correlation of individual enzyme levels and metabolic fluxes is generally weak in glycolysis, it resulted in the textbook assumption that glycolysis would typically not be controlled by enzyme abundance. However, simple protein–metabolite correlation analyses fail to account for the situation that, usually, multiple glycolytic enzymes change expression levels simultaneously. When taking these multifactorial relationships into account, the enzyme abundance patterns become highly predictive about metabolite levels (Zelezniak *et al*., 2018).

## V. THE ROLES OF GLYCOLYSIS AND ITS REGULATION IN HEALTH AND DISEASE

### (1) Glycolytic activity impacts global metabolic operations

**Fig. 6:**
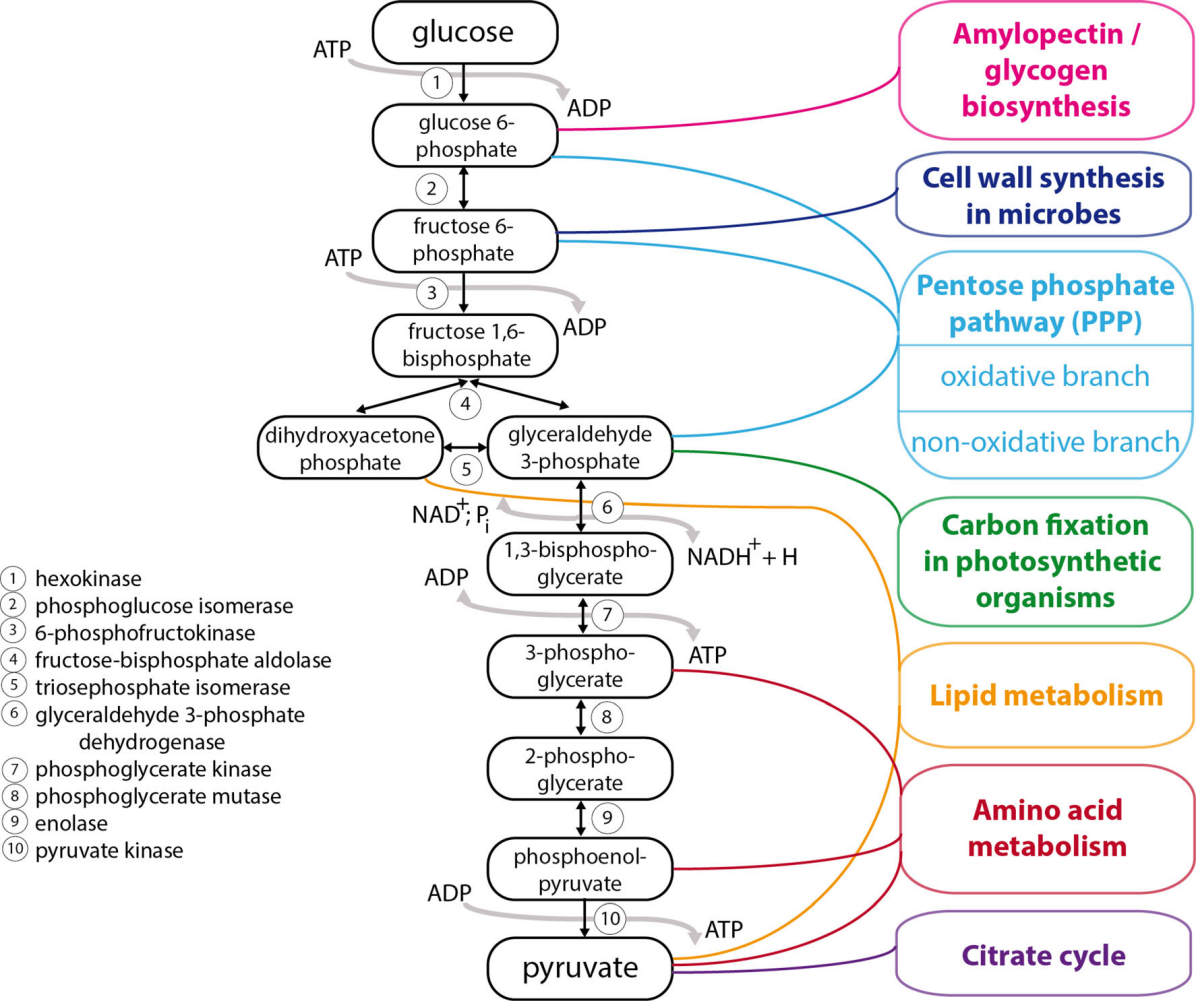
Schematic overview of glycolysis [Embden–Meyerhof–Parnas (EMP)] pathway and a selection of branching and indirectly connected pathways. The central role of glycolysis is exemplified by the high number of branching pathways for the downstream synthesis of metabolic intermediates. The strong interconnections of metabolic pathways requires a tight albeit dynamic regulation.

Many pathways branch out from glycolysis – through these branches, glycolysis can affect the activity of a broad range of metabolic pathways (Fig. 6). Pathways and reactions that branch from glycolysis include the PPP, carbon fixation in photosynthetic organisms, amylopectin and glycogen synthesis, lipid metabolism and the extraction of F6P to support the synthesis of 2‐amino sugars (GlcNAc, GalNAc and ManNAc) and GDP‐mannose and their downstream metabolites. Pyruvate supports the production of amino acids such as alanine, valine, leucine and isoleucine; 3PG supports serine biosynthesis (Amelio *et al*., 2014). Finally, glycolysis fuels cellular respiration and fermentation. Due to the many interconnections within the metabolic network and competition for metabolites between different pathways, the direction of fluxes and metabolite levels of glycolysis are subject to many layers of regulation, acting on different timescales (Van Heerden, Bruggeman & Teusink, 2015; Locasale, 2018). For example, the spatial–temporal distribution of glycolytic enzymes within the cell has been subject to recent research. Different studies across kingdoms suggest that glycolytic enzymes are not only freely dispersed in the cytosol but can form condensates and be found attached to cellular structures like plasma or organelle membranes (reviewed in Kierans & Taylor, 2024). This led to the hypothesis of the ‘metabolon’ or ‘glycosome’ – a functional, organelle‐like, complex in which glycolysis enzymes co‐localise to channel the intermediates between the enzymes of this pathway (Fuller & Kim, 2021). These structures are predicted to form transiently to reduce competition for glycolysis intermediates with other pathways, e.g. during situations of hypoxic stress in yeast and mammalian cells (Kierans *et al*., 2023; Fuller & Kim, 2021; Jin *et al*., 2017) or nutrient deprivation in yeast (Narayanaswamy *et al*., 2009).

While the EMP pathway supplies many other pathways with metabolites, it also depends on outside reactions to recover the NAD^+^ which was reduced by GAPDH to NADH. In most cells, reoxidation of NADH occurs either by cellular respiration (using oxygen as the terminal electron acceptor), *via* anabolic redox reactions, or *via* metabolites as electron acceptors in fermentation, which are then released as overflow. For example, pyruvate is used by LDH to form lactate during homolactic fermentation in animals, while ethanol fermentation converts the intermediate pyruvate to ethanol in yeast and several microbes, but also in fish of the genus *Carassius* (Fagernes *et al*., 2017). Some bacteria use mixed acid fermentation, a more complex process that can produce lactate, ethanol, acetate, formate, succinate and CO_2_ (Escherich, 1888). Still other bacteria use pyruvate as an electron acceptor leading to acetoin, butanediol and ethanol or to short‐chain fatty acids such as propionate and butyrate.

But how do cells that are capable of both respiration and fermentation ‘decide’ which of these metabolic pathways to use? To answer this question, their specific outcomes and benefits must first be understood. Because glucose is not further oxidised beyond pyruvate in glycolysis, fermentation produces only a small fraction of the stoichiometrically maximum possible 38 ATPs, in mammals, that can be generated through oxidative phosphorylation per molecule of glucose (Aguilera & Benítez, 1988). In the yeast *S. cerevisiae*, this would be a maximum of 28 ATP due to the different molecular composition of the respiratory chain (de Kok *et al*., 2012). However, biological efficiency is significantly more complex than can be described by the stoichiometric ATP yield. Fermentation can be the more favourable mode of metabolism, even in the presence of oxygen. While glycolysis produces ATP with ten cytosolic enzymes, energy production *via* the citric acid cycle and the respiratory chain requires complex, membrane‐bound enzymatic machinery, compartmentalisation, metabolite transporters and oxygen. It is thus more costly because it requires an upfront energy investment in the biosynthesis of enzymes and transporters and has a much slower turnover (Basan *et al*., 2015; Malina *et al*. 2021; Chen & Nielsen, 2019). Hence, fermentation can be advantageous and is used by many cells, both eukaryotic and bacterial, even in the presence of oxygen – a behaviour called, for historical reasons, either the Crabtree or the Warburg effect (Pfeiffer & Morley, 2014; Vazquez *et al*., 2010) (see Section V.1.*a*). The reverse behaviour is termed the Pasteur effect and involves cells shifting from fermentation to aerobic respiration upon high oxygen availability, with an accompanying decrease of their anabolism owing to the generation of more molecules of ATP per molecule of glucose.

In recent years, it has become increasingly evident that low rates of glucose uptake, and thus low glycolytic flux, are linked with respiratory metabolism. However, high rates of glycolysis cause a switch to aerobic fermentation. For microbes, it has often been conjectured that the level of extracellular glucose determines whether a cell uses respiration or aerobic fermentation (Meijer *et al*., 1998). However, recent experiments suggest that it is more precisely the *rate* of glycolytic flux which determines the fate of pyruvate (i.e. further processing *via* the citric acid cycle *versus* the fermentative pathway). Experiments performed in genetically modified yeast with different glucose uptake rates confirmed glycolytic flux as the decisive variable for the metabolic phenotype of a cell (Elbing *et al*., 2004). Besides the fate of pyruvate, studies on off‐branching pathways demonstrated that more glucose is diverted into storage metabolism at low glycolytic flux in *Saccharomyces cerevisiae* (e.g. Küenzi & Fiechter, 1972; Lillie & Pringle, 1980; François & Parrou, 2001; Teusink *et al*., 1998).

Glycolytic flux has a similar impact on the mode of metabolic operation in other species as well. For instance, *Bacillus subtilis* and *Escherichia coli* start to produce acetate at high glycolytic rates (Basan *et al*., 2015; Chubukov *et al*., 2013); mammalian cells (e.g. cancer cells) excrete lactate (Tanner *et al*., 2018) (the Warburg effect, see Section V.1.*a.iii*). Plants are generally resistant to changes in glycolysis flux (Rontein *et al*., 2002), and deviations are rather triggered by low oxygen availability [e.g. in root tissues (Drew, 1997) or during flooding (Roberts *et al*., 1984)]. However, the pollen of maize (*Zea mays* L.; Freeling & Bennett, 1985) and tobacco (*Nicotiana tabacum* L.; Bucher *et al*., 1995), as well as some plants exposed to environmental stressors have been found to produce acetaldehyde and ethanol even under aerobic conditions (Kimmerer & Kozlowski, 1982; Tadege, Dupuis & Kuhlemeier, 1999). All of these ‘fermentation’ end compounds originate from pyruvate and are eventually excreted into the extracellular environment *via* different routes. Genetic manipulations that increase or decrease the glycolytic flux lead to metabolic phenotypes reflected by respectively increased or decreased degrees of fermentation. For instance, replacing a ‘low‐activity’ with a ‘high‐activity’ PK allele (*pyk1*) in *Schizosaccharomyces pombe* led to an increased glycolytic rate and a switch to fermentative metabolism (Kamrad *et al*., 2020). Similarly, in *S. cerevisiae*, when flux is restricted by replacing a highly processive PK with one of lower activity, respiratory activity increases (Grüning *et al*., 2011). Moreover, in *Pichia pastoris*, a yeast species that normally only uses respiration, overexpression of a single specific transcription factor led to upregulation of the glycolytic genes and a significant increase in glucose uptake and fermentative metabolism (Ata *et al*., 2018). Thus, glycolytic flux seems to be a ‘decision maker’ for the guidance of molecules through the metabolic network.

**Fig. 7:**
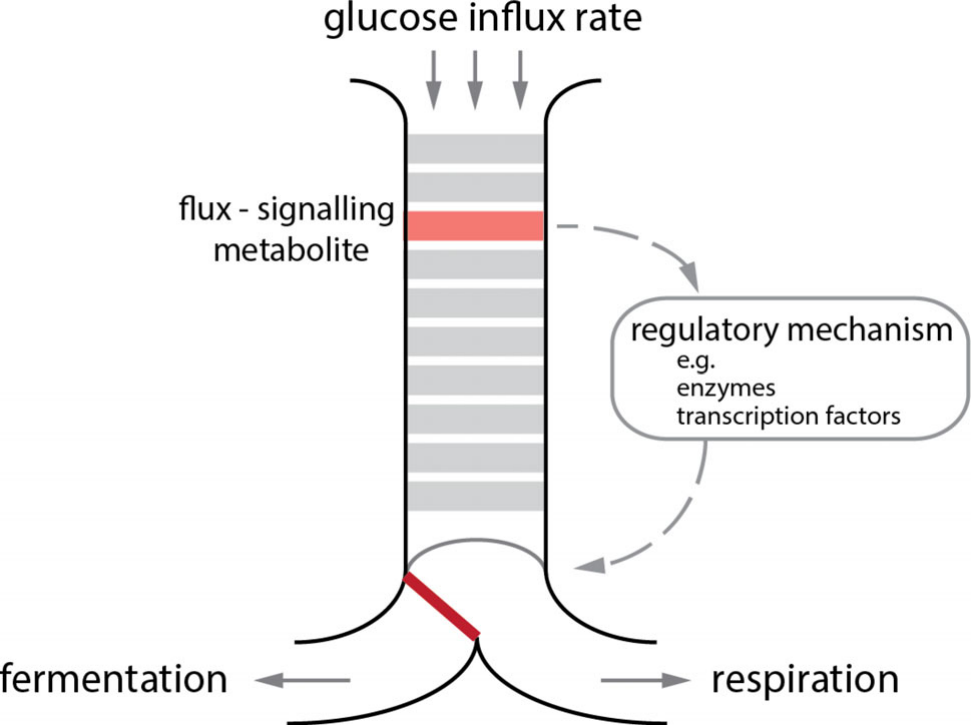
Molecular mechanisms of glycolytic flux sensing and their implications for cellular energy metabolism. High glycolytic flux correlates with reduced levels of oxidative phosphorylation and fermentation of pyruvate, while low glycolytic flux correlates with pyruvate influx into the citric acid cycle and increased rates of respiration. ATP and some intermediates have been proposed as flux‐signalling metabolites, among these phosphoenolpyruvate (PEP), cyclic AMP (cAMP) and fructose‐1,6‐bisphosphate (F1,6BP). In particular, F1,6BP and flux are linearly correlated in many organisms, and F1,6BP modulates the activity of several enzymes and signalling proteins. Although this basic concept is widely accepted, some important mechanistic details remain elusive. For example, it is subject to ongoing research how the metabolites are shared or transported across organelles.

This raises the question of how cells sense glycolytic flux. It has been proposed that certain metabolites – e.g. those whose concentrations strictly correlate with the flux through the respective pathway, ideally but not necessarily in a linear manner – act as flux signals (Fig. 7). If such a flux‐signalling metabolite is also involved in biomolecular interactions (e.g. binding to enzymes, RNA or proteins), it could exert regulatory functions in a flux‐dependent manner (Kotte, Zaugg & Heinemann, 2010; Huberts, Niebel & Heinemann, 2012; Litsios *et al*., 2018). One such example is cAMP. In bacteria, where glucose is taken up and phosphorylated through PEP group translocation, PEP/pyruvate (Bettenbrock *et al*., 2007) has been shown to correlate with the phosphorylation state of the EIIA component of the phosphotransferase system, which in turn regulates the levels of cAMP (Bettenbrock *et al*., 2007). The levels of PEP and pyruvate were also proposed to exert a flux‐signalling function (Kotte *et al*., 2010; Litsios *et al*., 2018). The archetypal flux‐signalling metabolite, however, is the glycolytic intermediate F1,6BP. Its concentration at steady‐state conditions was shown to correlate linearly with the magnitude of glycolytic flux in yeasts (Christen & Sauer, 2011; Huberts *et al*., 2012), *Escherichia coli* (Kochanowski *et al*., 2013), *Bacillus subtilis* (Chubukov *et al*., 2013) and mammalian cells (Tanner *et al*., 2018), and it is likely that the level of F1,6BP even reports on dynamic flux changes. Specifically, F1,6BP concentrations rise abruptly when a glucose pulse is provided to budding yeast growing under glucose‐limited conditions (Visser *et al*., 2004). This specific metabolite increase is consistent with the increased glucose influx.

But how is this linear relationship between flux and metabolite levels established, and how is it made independent of any possible changes in enzyme expression? In *Escherichia coli*, the combination of all glycolytic enzymes from FBA down to PK was reported to be responsible for the establishment of the linear flux–F1,6BP correlation. This set of reactions includes critical feedforward allosteric activation of PK by F1,6BP, which is responsible for the linearisation of the input–output relationship between flux and F1,6BP levels over a broad F6P concentration range (Kochanowski *et al*., 2013). In a theoretical analysis, at high expression levels of the enzymes in lower glycolysis, changes in these enzymes' abundances did not change in the input–output relationship (Kochanowski *et al*., 2013). And, indeed, these enzymes seem to be over‐abundant; for example, it was shown in *Drosophila* (Eanes *et al*., 2006) and mammalian cells (Tanner *et al*., 2018) that the levels of the enzymes of lower glycolysis do not exert any control over the flux.

If F1,6BP levels carry information about the glycolytic flux, what is the mechanism that links these levels to regulatory actions? F1,6BP was shown to control the activity of transcription factors in bacteria. In *Escherichia coli*, for instance, it regulates the activity of Cra, likely *via* its derived compound F1P (Bley Folly *et al*., 2018). In *Bacillus subtilis*, F1,6BP negatively modulates the activity of CggR, a transcriptional repressor of lower glycolytic genes (Doan & Aymerich, 2003; Zorrilla *et al*., 2007), and modulates PEP group translocation (Hueck & Hillen, 1995), which is responsible for glucose uptake. F1,6BP also directly modulates the activity of enzymes in different organisms. For example, in mammalian cells F1,6BP inhibits the interaction of FBA with the cytoskeleton (Kusakabe, Motoki & Hori, 1997). In *Lactococcus lactis*, F1,6BP activates PK (Kochanowski *et al*., 2013) and is necessary for the production of lactate (Voit, Neves, and Santos 2006). In *Escherichia coli*, increasing levels of F1,6BP inhibit two enzymes of the ED pathway, namely PEPS and G6PD (Piazza *et al*., 2018). The inhibition of PEPS occurs indirectly *via* allosteric activation of its regulating kinase and effectively redirects high glycolytic flux from respiration towards fermentation. G6PD is responsible for the production of NADPH, thus its inhibition by F1,6BP might represent a control mechanism of flux allocation between glycolysis and the PPP. F1,6BP also impacts the activity of signalling proteins – specifically, it was found to couple glycolytic flux to activation of the small G‐protein Ras (Peeters *et al*., 2017). In mouse embryo fibroblasts and in liver, it has also been found that increased levels of F1,6BP in response to glucose availability are sensed by FBA and prevent the formation of a lysosomal complex required for the activation of AMP‐activated protein kinase, a central regulator of energy homeostasis. (Zhang *et al*., 2017). In budding yeast and isolated rat liver, F1,6BP obstructs oxidative phosphorylation (Díaz‐Ruiz *et al*., 2008) by inhibiting mitochondrial complexes III and IV (Díaz‐Ruiz *et al*., 2008), likely by closing mitochondrial unspecific channels (Zizi *et al*., 1994; Lee, Zizi & Colombini, 1994). Furthermore, in ccRCC kidney cancer cells, F1,6BP suppresses the activity of the NADPH oxidase isoform NOX4 (Wang, Wu & Qiu, 2019). These regulatory actions are in line with the above discussed model in which cells tend to use fermentation at high glycolytic rates, when F1,6BP levels are high. This metabolic mode likely requires inhibition of oxidative phosphorylation, which could partly be accomplished by F1,6BP.

In concordance with the idea that glycolytic flux determines the global metabolic phenotype, differences in glycolysis between individual cells can lead to metabolic heterogeneity within a cell population. Single‐cell‐based analyses of an *Escherichia coli* population revealed a split into two phenotypes upon a sudden switch from glucose to a gluconeogenic substrate. This phenotype is determined by the glycolytic flux prior to the nutrient shift (Kotte *et al*., 2010). Furthermore, in budding yeast, a fraction of wild‐type cells are naturally unable to cope with a sudden surge in glucose availability due to a subsequent imbalance between the reactions of upper and lower glycolysis. Such cells fail to reach a steady state. The high activity of the ATP‐investing upper part of glycolysis does not match the ATP‐producing lower part, thus leading to the accumulation of intermediates such as F1,6BP. A main factor influencing metabolic success is the transient efficient release of inorganic phosphate upon glucose addition, which is dynamically ensured by a futile cycle installed in trehalose metabolism (Wang *et al*., 2019). Such findings highlight the need to assess metabolic rates in individual cells and also demonstrates the decisive role of glycolytic flux in cell fate decisions upon nutrient shifts. But why is glycolytic flux such a powerful conductor in the concert of metabolic pathways? Why do cells excrete certain energetically valuable metabolites upon high glucose uptake/glycolytic flux? In recent years, different hypotheses have been presented to explain this counterintuitive behaviour. These explanations range from constraints in membrane occupancy (e.g. Zhuang *et al*., 2011; Szenk, Dill & de Graff, 2017) and protein allocation (e.g. Basan *et al*., 2015; Chen & Nielsen, 2019) to thermodynamics (e.g. Noor *et al*., 2014; Park *et al*., 2016). According to the protein‐allocation hypothesis, the amount of protein required for respiration is problematic compared to what is needed for fermentation (simply because the latter consists of a shorter metabolic pathway). At high glycolytic rates, which in principle allow for rapid cell growth, the cell would also need to synthesise more ribosomes (Metzl‐Raz *et al*., 2017). If there are limited protein‐synthesis resources, switching to the shorter and less‐efficient fermentative pathway might still be the optimal strategy (Xia *et al*., 2022; Mori *et al*., 2023).

Meanwhile, the thermodynamics hypothesis suggests that there is an upper limit for the Gibbs energy dissipation rate (i.e. the rate at which energy is lost during metabolic operation) (Niebel *et al*., 2019). Arguably, many microbial cells are selected for fast growth in nutrient‐replete conditions. When they grow under low‐glucose‐uptake conditions, the overall cellular Gibbs energy dissipation rate is also low. Pyruvate is directed to respiration, as this is the most stoichiometrically efficient way to exploit a consumed glucose molecule. However, if the glucose uptake rate increases, the cellular Gibbs energy dissipation rate increases too – until it reaches its upper limit. In order to increase the rate of glucose uptake further (which will cause additional dissipation), cells now need to reduce the flux through respiration (to ‘spare’ dissipation) and instead redirect pyruvate to a fermentative pathway. Thus, this flux reshuffling enables cells to accommodate higher glucose uptake and growth rates at the expense of using glucose in a less‐efficient manner.

#### (a) Pasteur, Crabtree and Warburg effects and their historical definitions

The pivotal role of glycolysis in metabolism has been established by scientific investigations stretching over two centuries. Its impact on global metabolic operation was first exemplified by the discovery of the Pasteur effect and, subsequently, of the Crabtree and Warburg effects.

##### (i) Pasteur effect

The Pasteur effect – named after Louis Pasteur, who first observed this phenomenon in the 19th century (Pasteur, 1858) – refers to the inhibition of fermentation and decrease of glycolytic rate by high levels of oxygen. In the presence of oxygen, cells can generate more ATP *via* oxidative phosphorylation than solely *via* glycolysis (Barnett & Entian, 2005).

##### (ii) Crabtree effect

The Crabtree effect, described by Herbert Crabtree in the 1920s, refers to the acceleration of glycolysis and inhibition of respiration by high levels of glucose or other fermentable substrates (Crabtree, 1929). The inhibition of mitochondrial respiration and promotion of fermentation by high glucose concentrations led this mode of metabolism to become popularly known as the ‘counter‐Pasteur’ or ‘inverted Pasteur’ effect (De Deken, 1966; Vadlakonda *et al*., 2013). The Crabtree effect is often used to describe metabolism in microbes, especially in different yeasts, although it is not exclusive to them. It has the same outcome as the Warburg effect (de Alteriis *et al*., 2018).

##### (iii) Warburg effect

The Warburg effect, described by Otto Warburg also in the 1920s, refers to a mode of metabolism found in many tumours, which show increased glucose uptake and fermentation rate and high levels of lactic acid secretion even under sufficient oxygen supply (Warburg, 1925; Warburg, Wind & Negelein, 1927). Thus, the effect is also known as ‘aerobic glycolysis’. Warburg performed his initial experiments with tumour tissue and not with tumour cells (Warburg, 1925), reporting that dysfunctional respiration and the subsequent increase in fermentation could be the main cause for cancer formation (Warburg, 1930; Warburg, 1956a,b). Although some tumours indeed have impaired respiration due to mutations in mitochondrial genes and/or reduced citric acid cycle activity (Bartman *et al*., 2023), the dysfunctionality of mitochondrial respiration was later refuted in other tumours and as a general explanation for the Warburg effect (reviewed in Frezza & Gottlieb, 2009; Cassim *et al*., 2020).

It became known early on that the Warburg effect is not consistent across different tumours and that non‐cancerous cell types can also display this mode of metabolism (Warburg, 1929), reviewed in (Potter, Newport & Morten, 2016). With today's (likely still incomplete) knowledge, it seems that (*i*) there are several different mechanisms and regulatory events that lead to metabolic states with the outcomes of what was once described as the Warburg effect, and (*ii*) cells rarely switch completely from respiration to glycolysis or *vice versa* – rather, they undergo a shift in the ratio between these modes. For example, tumours are almost always heterogeneous in terms of nutrient and oxygen supply (Sengupta & Pratx, 2016; Crabtree, 1929). Warburg performed his experiments in tumour slices which were likely not hypoxic; however, solid tumours eventually outgrow their oxygen supply, and the tumour microenvironment therefore demands changes in metabolism. Expression of glycolytic enzymes is upregulated under hypoxia (Robin, Murphy & Theodore, 1984), e.g. mediated by hypoxia‐inducible factor (HIF) (Semenza *et al*., 1994; Kierans & Taylor, 2021). Thus, strictly speaking, cells switch to anaerobic glycolysis, not to aerobic glycolysis according to the commonly used definition of the Warburg effect. However, such glycolytic regions produce and export large amounts of lactate which can then serve as the substrate for oxidative phosphorylation in neighbouring well‐oxygenated parts – almost in a pseudo‐organ way (Li *et al*., 2022b; Hsu & Sabatini, 2008). This phenomenon has been termed the ‘Reverse Warburg effect’ (Sonveaux *et al*., 2008; Pereira‐Nunes *et al*., 2020).

**Fig. 8:**
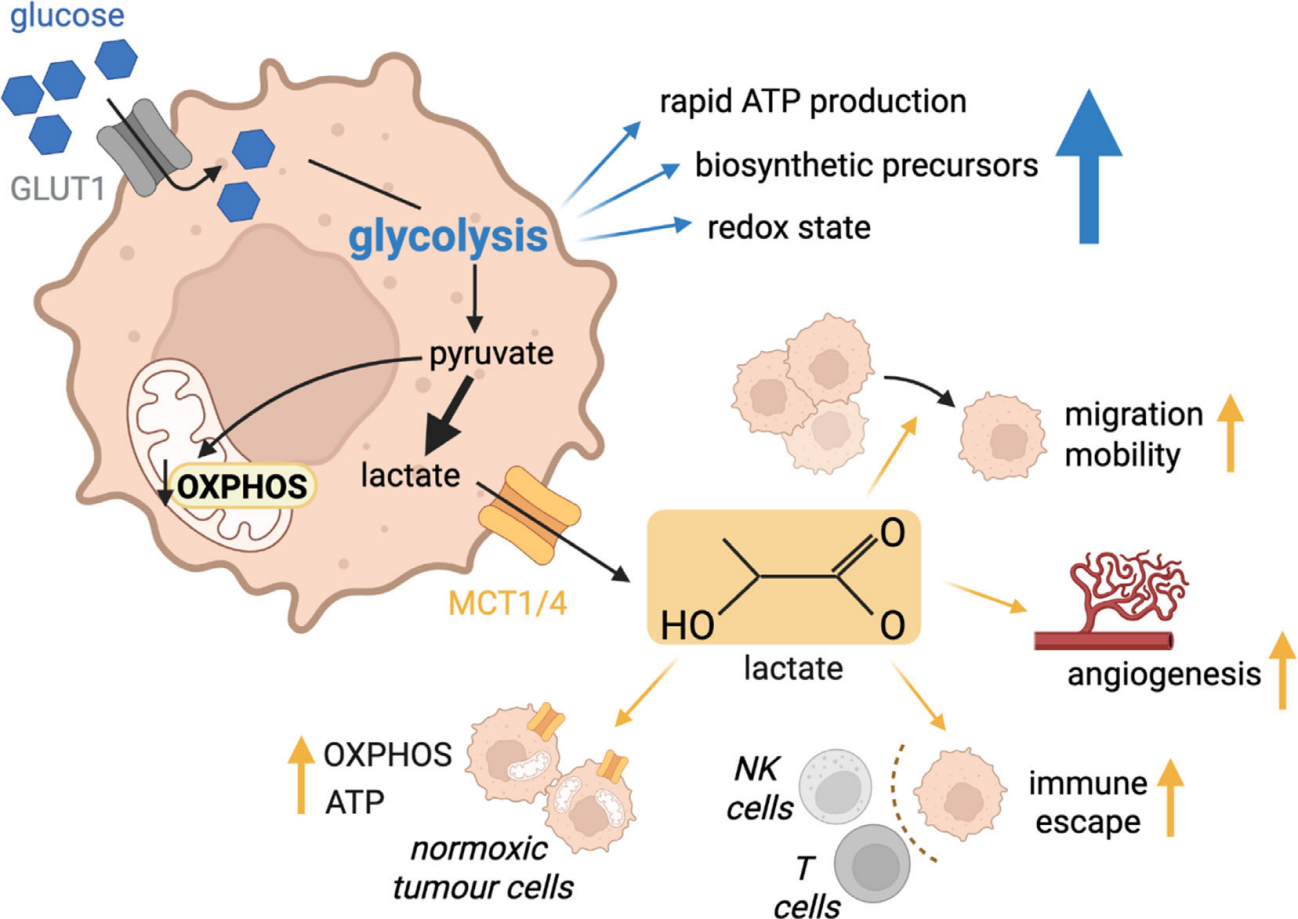
Metabolic changes in tumours and their physiological implications. The Warburg effect describes the metabolic behaviour of many, but not all, cancer cells as increasingly consuming glucose and shifting from respiration to lactic fermentation. In the oxygen‐depleted regions of tumours, glycolysis runs at increased rate and produces an excess of pyruvate, which is not channelled into energy production through mitochondrial oxidative phosphorylation but rather converted into lactate. Under these conditions, lactic fermentation provides access to rapid ATP production and enables the energy metabolism to flow. In the tumour microenvironment, lactate plays a multitude of roles and regulates critical processes such as promoting oxidative phosphorylation in the well‐oxygenated peripheral cancer cells (‘reverse Warburg effect’) and stimulating growth. Additionally, lactate promotes the creation of new blood vessels, increases cell motility and metastasis potential, and protects cancer cells against the host immune system. MCT1/4, monocarboxylate transporters 1–4; OXPHOS, oxidative phosphorylation; NK cells, natural killer cells.

Furthermore, large amounts of lactate acidify the tumour microenvironment, which in turn, might promote tumour growth (Warburg *et al*., 1927). Exported lactate plays important signalling roles and can stimulate angiogenesis, migration and metastasis and inhibit immune cells such as T cells or natural killer (NK) cells (Fig. 8) (reviewed in San‐Millán & Brooks, 2017; Pérez‐Tomás & Pérez‐Guillén, 2020). It also directly regulates the cell cycle (Liu *et al*., 2023). Aerobic glycolysis thus directly contributes to tumour immune escape (Hanahan & Weinberg, 2011). Alternatively, oncogenic mutations can drive a switch to aerobic glycolysis or stabilise this mode of metabolism. Hence, the Warburg effect seems to be context dependent, but still beneficial to tumour development and growth (reviewed in Hsu & Sabatini, 2008; Sengupta & Pratx, 2016; Liberti & Locasale, 2016; Jose, Bellance & Rossignol, 2011).

### (2) Inborn errors of glycolysis enzymes that lead to human metabolic disease

**Fig. 9:**
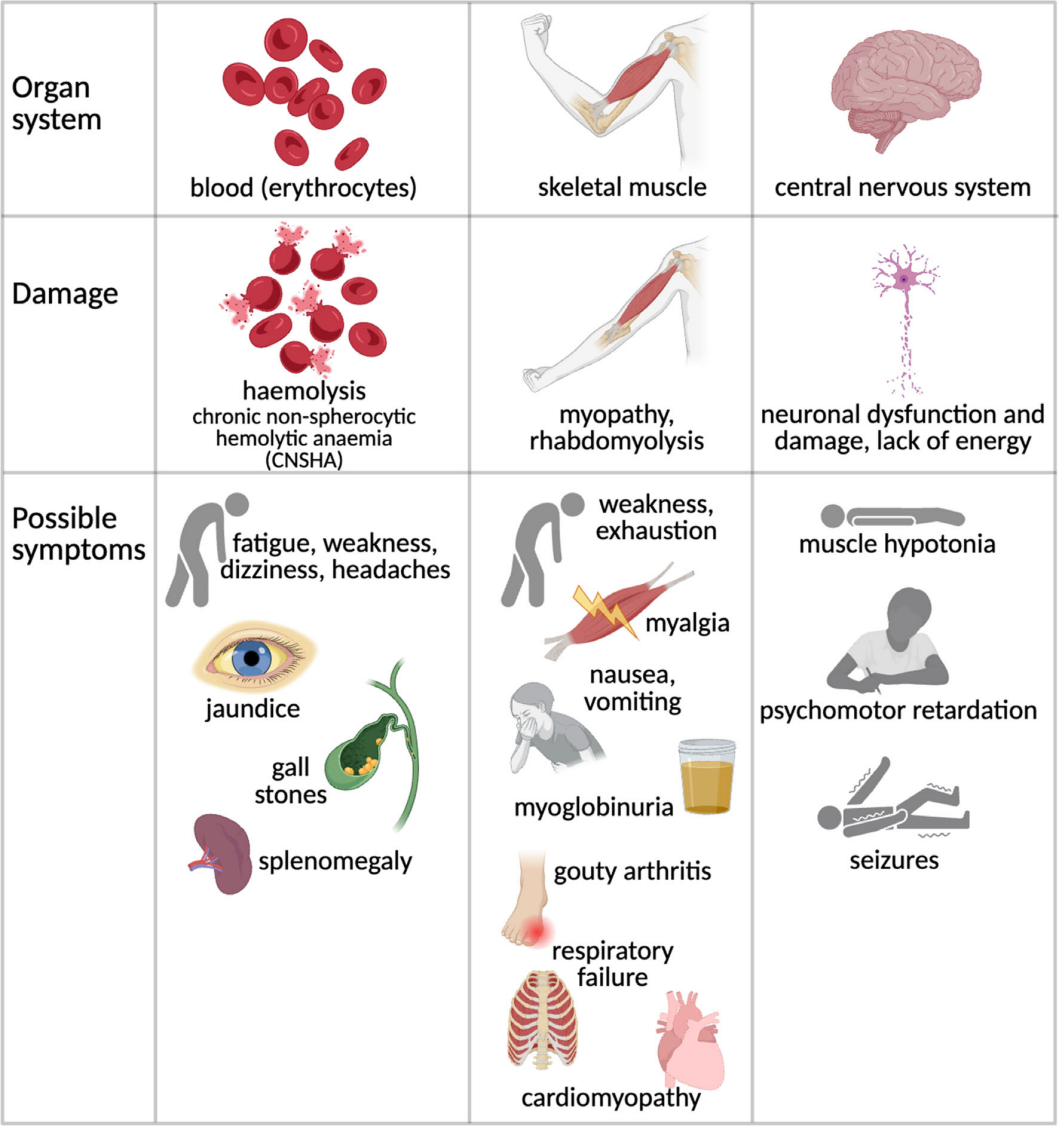
Organs commonly affected by monogenic disorders of glycolysis and their related symptoms. Monogenic diseases affecting glycolytic enzymes mostly cause their respective symptoms due to an energy deficit in cell types that depend on ATP generated in glycolysis.

Inborn errors of metabolism have been described for almost all enzymes in glycolysis, except for GAPDH. Glycolytic enzymopathies are usually very rare but severe. All are autosomal recessive disorders save for X‐linked recessive PGK deficiency and autosomal dominant GK deficiency. Many of these affect erythrocyte biology. Mature erythrocytes lack mitochondria, making them completely dependent on glycolysis for the production of ATP. Hence, a common symptom of glycolytic enzymopathies is haemolytic anaemia (or chronic non‐spherocytic haemolytic anaemia, CNSHA), resulting in clinical features including jaundice, splenomegaly, an increased incidence of gallstones and common symptoms of anaemia (Fig. 9) (Kugler & Lakomek, 2000). Glycolysis is also the most important energy source in other tissues including types of skeletal muscle fibres, making metabolic myopathy another leading symptom of glycolytic enzymopathies.

#### (a) Glycolytic disorders

##### (i) Hexokinase deficiency

There are four isozymes of human HK in most cell types, each encoded by a different gene (*HK1–4*). Erythrocytes express two major isoforms formed by alternative splicing of the *HK1* gene: HK‐R and HK‐1. Hexokinase deficiency (OMIM database #235700) is caused by mutations in the *HK1* gene on chromosome 10q22 (Jamwal *et al*., 2019) and manifests clinically with CNSHA of varying severity. Mutations of *HK1* are reportedly very rare (Jamwal *et al*., 2019).

Under physiological conditions, HK4 (GK) phosphorylates only glucose at a low affinity. GK is expressed only in mammalian liver and pancreatic islet β‐cells. GK deficiency can cause diabetes type II (OMIM #125853; 125851), while an activating GK mutation causes hyperinsulinemic hypoglycaemia (OMIM #602485); both of these disorders are autosomal dominant (Matschinsky, 2005). Homozygous mutations in the *GK* gene causing complete GK deficiency result in permanent neonatal diabetes mellitus‐1; this is a rare autosomal recessive disorder characterised by severe hyperglycaemia, requiring insulin treatment soon after birth (Njølstad *et al*., 2001).

##### (ii) Phosphoglucose isomerase deficiency

Pathogenic *PGI* variants, located on chromosome 19q13, are mostly point mutations that lead to enzymatic instability, but nonsense and splicing variants have also been described (Fermo *et al*., 2019). PGI deficiency (OMIM #613470) was first reported in 1968 (Baughan *et al*., 1968), and so far 70 cases have been identified. Decreased PGI activity results in increased G6P levels and a lower rate of glycolysis. This disorder leads to CNSHA (Hirono *et al*., 2001; Kugler & Lakomek, 2000) and has also been associated with hydrops fetalis, immediate neonatal death and neurological impairment (e.g. hypotonia, neurodevelopmental delay and seizures) (Ravindranath *et al*., 1987; Park *et al*., 2020). Tissue‐specific isozymes have not been detected, and decreased PGI activity therefore affects all tissues (Paglia & Valentine, 1974).

A patient with CNSHA and drug‐resistant epilepsy was recently treated with a ketogenic diet (Park *et al*., 2020); this reduced seizures significantly and attenuated haemolysis. Based on this report, the authors hypothesised that the neurological phenotype sometimes seen in PGI deficiency might result from an energy deficit in the brain, which may be improved by providing non‐carbohydrate energy in the form of ketones. Other suggested causes for the neurological phenotype are the link between monomeric PGI and neuroleukin or confounding factors, such as kernicterus or other genetic defects that may contribute to the clinical phenotype (Kugler & Lakomek, 2000; Fermo *et al*., 2019).

##### (iii) Phosphofructokinase deficiency

PFK deficiency (OMIM #232800), or glycogen storage disease VII (GSD VII), is also named Tarui disease after Seiichiro Tarui, who first observed the condition in 1965 (Tarui *et al*., 1965). About 100 cases have been reported worldwide (Musumeci *et al*., 2012).

PFK is a strongly regulated enzyme of glycolysis (Filosto *et al*., 2019). It is a tetrameric enzyme which can be composed of the subunits: PFK‐L (liver), encoded by the *PFKL* gene on chromosome 21q22; PFK‐M (muscle), encoded by *PFKM* on 12q13; and PFK‐P (platelet), encoded by *PFKP* on 10p15 (DiMauro & Spiegel, 2011). PFK‐P is also known as fibroblast type (PFK‐F) (Toscano & Musumeci, 2007). The tetramers formed by these subunits vary among tissues (Musumeci *et al*., 2012). In mature human muscle, only the M subunit is expressed, and the M4 homotetramer is therefore the only PFK isoform produced; meanwhile, erythrocytes express both the M and L subunits, which form the corresponding M4 and L4 homotetramers but can also form hybrid isoforms. These differences have clinical consequences; for instance, in the typical PFK deficiency caused by mutations in the *PFKM* gene, PFK activity is completely abrogated in muscle cells but only partially deficient in erythrocytes (DiMauro & Spiegel, 2011).

Human PFK deficiency is categorised into four clinical phenotypes – classic, infantile, late‐onset and haemolytic – which differ by age of onset and symptoms (Filosto *et al*., 2019). The classic form is the most common type of PFK deficiency, with onset in early childhood characterised by myalgia and contractures following isometric or intense dynamic exercise (Filosto *et al*., 2019). Jaundice, nausea and vomiting following strenuous exercise are common; less‐frequent symptoms include rhabdomyolysis, as well as haemolytic anaemia causing myoglobinuria a few hours post‐exercise (Toscano & Musumeci, 2007). A prominent secondary abnormality is gouty arthritis due to ‘myogenic hyperuricemia’ (DiMauro & Spiegel, 2011), so called because of hyperproduction of uric acid as a consequence of metabolic crisis in the exercising muscle (Nakajima *et al*., 2002).

The rapidly progressive fatal infantile form has been reported in infants in association with progressive loss of muscle tone (‘floppy babies’) and muscle weakness (Toscano & Musumeci, 2007). Survival rate is low, and the cause of death (which usually occurs within two years) is often respiratory failure.

The late‐onset form is commonly characterised by myopathy and weakness, although some individuals have difficulty with sustained exercise starting in childhood.

Finally, the haemolytic form is characterised by haemolytic anaemia with no signs or symptoms of muscle involvement. Erythrocytes break down due to their partial PFK deficiency, leading to an increase in plasma bilirubin levels. However, affected people usually experience no symptoms (Filosto *et al*., 2019).

##### (iv) Aldolase A deficiency (GSD XII)

Aldolase A (ALDOA, F1,6BP aldolase A) deficiency (GSD type XII, OMIM #611881) is caused by pathogenic variants in *ALDOA* located on chromosome 16p11. It is a very rare cause of CNSHA (alone or in combination with neurological abnormalities) (Beutler *et al*., 1973; Miwa *et al*., 1981), and myopathy or isolated episodic rhabdomyolysis with onset generally within the first months of life (Kreuder *et al*., 1996). If not promptly recognised, death from severe rhabdomyolysis can occur in myopathic patients (Yao *et al*., 2004). As ALDOA is the only aldolase isozyme expressed in erythrocytes and muscle, its deficiency is predicted to affect these tissues more severely than others as a consequence of impaired ATP generation (Wamelink, Valayannopoulos & Garavaglia, 2016). Only eight patients from five families have been reported so far (Mamoune *et al*., 2014).

##### (v) Triose phosphate isomerase deficiency

TPI is catalytically active only in its stable homodimeric form (Orosz, Oláh & Ovádi, 2009). TPI deficiency (OMIM #615512) was initially described in 1965 (Schneider *et al*., 1965), and fewer than 50 patients were identified up to the early 2000s (Schneider, 2000). It is caused by pathogenic variants in the *TPI1* gene, located on chromosome 12p13.31, with most patients carrying the same conserved substitution, E104D. Recent work suggests that, rather than *directly* inactivating TPI, the prevalent substitution E104D instead alters TPI dimerization (Schneider *et al*., 1996; Ralser *et al*., 2006; Conway *et al*., 2018), thus producing a less‐active mutant through enzyme degradation rather than through a direct reduction in specific activity (Ralser *et al*., 2006; Segal *et al*., 2019). The resultant block in glycolysis allows DHAP to accumulate, which causes increased formation of the reactive methylglyoxal, leading to the increased formation of AGEs (Orosz *et al*., 2009). The presence of mutant TPI may also result in the formation of toxic protein aggregates (Orosz *et al*., 2009). Other, rare TPI variants can also impair substrate binding to the active site (Conway *et al*., 2018) and reduce the enzyme's thermostability (Li *et al*., 2013).

Since there is only one isozyme of TPI, this deficiency impacts several tissues (Glader, 2013) and manifests as a rare multisystem disorder. It is the most severe glycolytic enzymopathy and lethal, frequently in early childhood (Orosz *et al*., 2009) Clinically, patients with the E104D variant present a severe phenotype that requires ventilator support in the second year of life (Sarper *et al*., 2013), and most patients die before the age of six (Singer *et al*., 2016). Genotype identification is therefore critical for prediction of life expectancy in TPI deficiency patients (Orosz, 2012). Characteristic symptoms of TPI deficiency include chronic haemolytic anaemia, cardiomyopathy, severe neurological dysfunction and susceptibility to recurrent infections (Schneider, 2000). Neonatal jaundice may also occur (Gregg & Prchal, 2018). Associated neurological abnormalities include spasticity, motor retardation and hypotonia (Glader, 2013) that vary in onset and severity but do not appear to be directly correlated with enzyme activity; a genotype–phenotype relationship has been suggested (Conway *et al*., 2018). Muscle weakness can affect breathing and heart function (cardiomyopathy). Diaphragm weakness can cause breathing problems and ultimately leads to respiratory failure.

Homozygosity of pathogenic *TPI* variants is rare (Valentin *et al*., 2000). To our knowledge, no null alleles have been reported in homozygosity, which points to incompatibility of a complete lack of TPI activity with foetal life. By contrast, cases of compound heterozygosity have been reported more often (Orosz 2012), meaning that different combinations of heterozygous pathogenic variants might result in an overall enzyme activity that is still able to support life (Glader, 2013; Mohrenweiser & Fielek, 1982). Indeed, heterozygous carriers appear asymptomatic if TPI activity is at least 50% that of unaffected cells, while in homozygous or compound heterozygous patients, TPI activity of erythrocytes, platelets or lymphocytes is between undetectable and 30% (Conway *et al*., 2018; Hollán *et al*., 1993; Valentin *et al*., 2000).

##### (vi) Phosphoglycerate kinase deficiency

PGK deficiency (OMIM #300653) was first described by Kraus, Langston & Lynch (1968); since then, fewer than 50 patients have been reported (Echaniz‐Laguna *et al*., 2019). PGK deficiency is a rare X‐linked metabolic disorder caused by pathogenic variants in the *PGK1* gene located on chromosome 10q13.1. Most affected patients are hemizygous males. Heterozygous females may exhibit only varying degrees of haemolytic anaemia depending on random X‐inactivation (Hirono *et al*., 2001).

PGK is mainly expressed as the PGK1 isoform, except in testes, where the enzyme is present as PGK2 (Echaniz‐Laguna *et al*., 2019). Tissues with high energy demand are especially sensitive to decreased PGK activity; therefore, PGK deficiency is a multisystem disorder with three major clinical presentations: CNSHA, central nervous system involvement (including seizures, intellectual disability, stroke and parkinsonism) and myopathy (with exercise intolerance, cramps and recurrent myoglobinuria). Most patients only show one or two of these presentations in different combinations (Hirono *et al*., 2001). The onset of PGK deficiency is generally in childhood, but an infantile onset has also been reported; its prognosis is variable, depending on the severity of the anaemia and on the presence of the other manifestations (Wamelink *et al*., 2016). Recently, a new phenotype was reported with predominant peripheral nervous system involvement resembling Charcot–Marie–Tooth disease (Echaniz‐Laguna *et al*., 2019). Some cases have been identified with juvenile and early‐onset parkinsonism, often responsive to levodopa (Morales‐Briceño *et al*., 2019).

##### (vii) Phosphoglycerate mutase deficiency

PGAM muscle deficiency (OMIM #261670), or GSD X, is caused by pathogenic variants in the *PGAM2* gene on chromosome 7p13, encoding muscle PGAM‐2 (PGAM2 enzyme). Only 16 cases of PGAM deficiency have been reported until 2018 (Kanungo *et al*., 2018; Tarnopolsky, 2018; Koo & Oskarsson, 2016). Although the disease has been associated with autosomal recessive inheritance, manifesting heterozygotes have been reported, an unexpected finding for a disease in which even homozygotes are affected only in extreme circumstances (Naini *et al*., 2009). Manifesting heterozygotes have also been reported in families with other defects in anaerobic glycolysis – e.g. McArdle disease (Hadjigeorgiou, 2009). People heterozygous for the mutated *PGAM2* gene may present some symptoms of PGAM deficiency associated with a 50% reduction in PGAM activity at the biochemical level (Bresolin *et al*., 1983).

Patients with PGAM deficiency typically present with exercise‐induced myalgias, muscle contractures, hyperCKemia and myoglobinuria with onset from childhood into as late as the 6th decade. Patients are asymptomatic at rest, but exercise can provoke severe symptoms including rhabdomyolysis (Koo & Oskarsson, 2016). Histochemistry and electron microscopy on muscle biopsies of PGAM muscle deficiency patients has revealed mild glycogen accumulation (Hadjigeorgiou 2009); this has been reported to be a frequent association with tubular aggregates (ordered stacks of tubules originating from the sarcoplasmic reticulum) that do not appear to be causal, although the specific trigger remains unknown (DiMauro & Spiegel, 2011). Tubular aggregates have never been associated with other, more common muscle GSDs or with other defects of terminal glycolysis (Naini *et al*., 2009).

##### (viii) Enolase deficiency

β‐enolase deficiency (OMIM #612932), or GSD XIII, is an extremely rare disorder, first diagnosed in 2001 (Comi *et al*., 2001). This patient carried two heterozygous missense variants and had a severe muscle enolase deficiency (5% of residual activity). Since then, only four more patients have been identified (Wigley *et al*., 2019). The disease is caused by pathogenic variants in the *ENO3* gene located on chromosome 17p13.2, which encodes β‐enolase. Clinically, enolase deficiency presents with exercise intolerance, cramps and exercise‐induced myalgia. As often seen in metabolic myopathies, the late appearance of fixed exercise intolerance may reflect permanent muscle damage, although a psychogenic origin is possible (Comi *et al*., 2001). Rhabdomyolysis followed by acute renal insufficiency with anuria was described in two patients by Wigley *et al*. (2019). Muscle biopsy showed a slight variability in fibre size, while electron microscopy demonstrated an accumulation of glycogen β particles in the sarcolemma.

##### (ix) Pyruvate kinase deficiency

PK deficiency (OMIM #266200) is the most common glycolytic enzymopathy; it is an important cause of CNSHA and has been reported in several hundred families (Hirono *et al*., 2001). PK plays a central role in the energy metabolism of erythrocytes since it catalyses the second reaction of ATP production. Hence, PK deficiency is thought to cause ATP depletion and an accumulation of glycolytic intermediates proximal to the metabolic block – particularly 2PG, 3PG and 2,3BPG – which may further impair glycolytic flux (Bianchi *et al*., 2019).

The *PKLR* gene is located on chromosome 1q22 and encodes two PK isoenzymes (the L‐ and R‐types) which are expressed in liver and erythrocytes, respectively. In most cases, heterozygous carriers are asymptomatic, although exceptional cases with clinical symptoms have been reported (Hirono *et al*., 2001). Clinical severity can vary from affected foetuses deceased *in utero* with anaemia and nonimmune hydrops (Gilsanz *et al*., 1993) to very mild haemolysis. The majority of patients are detected at birth, while some only present symptoms during times of great physiological stress. The anaemia tends to improve with age. Splenomegaly is reported in about 80% of patients.

In human erythrocytes, PK deficiency has been found to protect against infection by and replication of *P. falciparum* (Ayi *et al*., 2008). Positive selection of the *PKLR* gene has been reported in sub‐Saharan African and Pakistani populations, which may be attributable to the malaria parasite (Berghout *et al*., 2012).

#### (b) Diagnosing inborn errors of glycolysis

For some defects, specific glycolysis intermediates can be measured, and indeed, in several instances altered metabolite concentrations led to the original discovery of the enzyme defects. For instance, in HK deficiency, 2,3BPG and G6P are decreased in erythrocytes; in TPI deficiency, the TPI substrate DHAP accumulates in erythrocytes; and in PK deficiency, 2PG, 3PG and 2,3BPG accumulate (Wamelink *et al*., 2016). Histochemical tests can be informative for PFK, PGAM and ALDOA deficiency. In enolase deficiency, lactate does not rise during a forearm ischemic test. Measurement of the enzyme activity in erythrocytes or muscle and/or identification of pathogenic genetic variants in the corresponding genes is also necessary to confirm a diagnosis.

#### (c) Treating inborn errors of glycolysis

Current management for most glycolytic disorders remains supportive, including erythrocyte transfusions, chelation therapy to remove iron overload due to haemolysis, splenectomy in cases of severe anaemia, avoiding strenuous exercise to avoid rhabdomyolysis or assisted ventilation to treat paralysis of the diaphragm in case of TPI deficiency (Wamelink *et al*., 2016). Bone marrow transplant has been reported for HK deficiency, with a positive outcome at one year follow‐up (Khazal *et al*., 2016), and for PGK deficiency in cases with severe neurological deterioration (Rhodes *et al*., 2011). Transplantation should be considered prior to severe manifestations of the disease. Haematopoietic stem cell transplantation has been pursued in a small number of PK‐deficient patients with mixed outcomes (Grace, Layton & Barcellini, 2019). A targeted allosteric activator of PK has recently been approved for treatment of adults in the US, EU and UK and is currently being tested for paediatric use (Al‐Samkari & van Beers, 2021; Al‐Samkari *et al*., 2024). Enzyme replacement therapies for glycolytic defects have been discussed since the late 1990s. Despite promising early results – for instance, in the treatment of TPI deficiency *in vitro* (Ationu *et al*., 1999a,b) – enzyme replacement therapies remain unavailable for patients.

An infant with PFK deficiency (Swoboda *et al*., 1997) and, recently, a patient with PGI deficiency presenting with CNSHA and drug‐resistant epilepsy (Park *et al*., 2020) were treated with a ketogenic diet. Symptoms improved in both.

Although there is often no specific treatment for the patient themselves, detection of the index case may provide the opportunity for genetic counselling and prenatal diagnosis in further family members (Sarper *et al*., 2013). Furthermore, recent success with new modalities, such as protein‐stabilising drugs targeting the cystic fibrosis transmembrane conductance regulator in the treatment of cystic fibrosis (Graeber & Mall, 2023) may act as a door opener for the development of new therapeutics for glycolytic enzymopathies.

### (3) Glycolysis in immunity and infection

(Dys‐)regulation of glycolytic activity and metabolic reprogramming are frequently observed as physiological or pathological changes. As aforementioned, glycolysis is of particular importance in cancer research, but also in the fields of immunology and infectious disease. Indeed, biochemical analysis of immune cell metabolism reaches back to the 1960s (Oren *et al*., 1963). However, the concept and term ‘immunometabolism’ were only established in the last decade and now represent a vigorous area of research (O'Neill, Kishton & Rathmell, 2016). Some key examples of the involvement of glycolysis in immunological processes are provided below.

Glycolysis has been shown to be upregulated in cells of the innate and adaptive immune responses, such as dendritic cells (Krawczyk *et al*., 2010; Wculek *et al*., 2019) and B cells (Doughty *et al*., 2006), in response to stimulation of Toll‐like receptors (TLRs) and antigen receptors, respectively. Likewise, a shift to aerobic glycolysis occurs in both pro‐inflammatory and anti‐inflammatory (M1 and M2) macrophages upon activation, although it is more pronounced in the M1 type (Zhao, Raines & Huang, 2020; Rodríguez‐Prados *et al*., 2010). This metabolic shift had been principally described as early as 1970 (Hard, 1970). Macrophages express the pattern recognition receptor TLR4 on their surface. TLR4 interacts with lipopolysaccharide (LPS), an antigenic component of the outer membrane of Gram‐negative bacteria (Pålsson‐McDermott & O'Neill, 2004). Activation of TLR4 by LPS increases the levels of succinate, an intermediate product of the citric acid cycle, and leads to the stabilisation of hypoxia inducible factor‐1alpha (HIF‐1α) (Tannahill *et al*., 2013; Selak *et al*., 2005). Stimulation of macrophages with LPS also leads to upregulation of PKM2 and the formation of complexes between inactive PKM2 dimers (or monomers) and HIF‐1α (Palsson‐McDermott *et al*., 2015). PKM2‐bound HIF‐1α is stabilised and drives transcription of HIF‐1α‐regulated genes. For example, HIF‐1α induces expression of the cytokine interleukin 1β (IL‐1β), thereby promoting the pro‐inflammatory macrophage response. Glycolytic enzymes are also upregulated in response to increased HIF‐1α‐mediated transcription. In LPS‐stimulated macrophages, GLUT1, HK3, 6‐phosphofructo‐2‐kinase/fructose 2,6‐bisphosphatase 3, phosphoglucomutase 2 and ENO2 are upregulated, correlating with the observed increase in glycolytic activity (Tannahill *et al*., 2013). Therefore, PKM2 modulates the shift to aerobic glycolysis observed in activated macrophages *via* its interaction with HIF‐1α, similar to the modulation of the Warburg effect described in tumour cells (Luo *et al*., 2011).

Glycolysis is also involved in the activation and differentiation of T cells. Before they encounter a cognate antigen, T cells are largely metabolically inactive – they display low rates of glycolysis, generate ATP predominantly *via* oxidative phosphorylation, and have a low biosynthetic turnover (Pearce *et al*., 2013). Upon activation, T cells differentiate into their effector phenotypes as either CD4^+^ T helper cells (T_h_) or CD8^+^ cytotoxic T cells (T_c_). This process is accompanied by large metabolic profile changes including a switch to aerobic glycolysis (and a corresponding reduction in oxidative phosphorylation), increased glutaminolysis and PPP, and higher rates of biosynthesis of proteins, lipids, and nucleotides (Pearce *et al*., 2013; Soto‐Heredero *et al*., 2020). This metabolic reprogramming supports growth and clonal expansion of activated T cells and likely involves multiple signalling pathways. Signalling through the T cell co‐stimulatory receptor CD28 has been shown to increase expression of GLUT1 (Frauwirth *et al*., 2002). The transcription factors HIF‐1α and Myc have both been suggested to mediate the activation‐induced upregulation of glycolytic enzymes (Wang *et al*., 2011; Shi *et al*., 2011; Almeida *et al*., 2016). Interestingly, the switch to aerobic glycolysis seems to promote both the proliferation of activated T cells and the differentiation of T cells into effector populations. For example, HIF‐1α‐dependent upregulation of glycolysis has been shown selectively to promote differentiation of the T_h_17 lineage, but not T_h_1 or T_h_2 differentiation, whereas blocking of glycolysis with 2‐DG had an opposite effect and promoted the generation of regulatory T cells (Shi *et al*., 2011).

Reminiscent of the Warburg effect, lactate secretion is also observed in activated T cells (Fox, Hammerman & Thompson, 2005; Brand, 1985; Almeida *et al*., 2016). Several hypotheses have been discussed to explain upregulation of glycolysis in an immunological context. Like other cells, immune cells require balancing of the NADH and NADPH pools, as well as sufficient energy supplies during the transition from low metabolic activity to fast proliferation and protein secretion. Furthermore, the glycolysis intermediate PEP has been shown to accumulate in activated T cells; this leads to increased intracellular Ca^2+^ levels and sustains signalling through nuclear factor of activated T cells, which is important for regulating T cell effector functions (Ho *et al*., 2015; Soto‐Heredero *et al*., 2020). Many other metabolites have been implicated in immune cell signalling and epigenetic regulation of the inflammatory response (reviewed in Soto‐Heredero *et al*., 2020).

A striking link between glycolysis and cytokine production was observed in T‐cells: the levels of produced interferon γ (IFN‐γ) and IL‐2 are decreased even when oxidative phosphorylation is inhibited (Chang & Wei, 2011). When T‐cells are cultured in glucose instead of galactose medium, IFN‐γ messenger RNA (mRNA) is preferentially associated with polysomes and therefore translated at higher rates. GAPDH binding to the 3′ untranslated region of IFN‐γ mRNA when not engaged in glycolysis, thereby inhibiting translation, has been suggested as the mechanism underlying this regulation (Chang & Wei, 2011). These observations highlight the role glycolytic enzymes can play as coupling factors between metabolism and immune cell effector function.

Following the increasing interest in immunometabolism, studies have emerged investigating the role of metabolism in infectious disease (Ayres, 2020). There is considerable metabolic crosstalk between pathogens and hosts: pathogens compete for glucose with the host, can alter oxygen levels at sites of infection, regulate or hijack host metabolism, and deploy various metabolic strategies to avoid detection by the host immune system, while hosts try to sense ‘foreign’ metabolites to detect pathogens and subsequently focus their immune cell metabolism on counteracting infection (Olive & Sassetti, 2016).

An example of a metabolic ‘arms race’ between host and pathogen comes from the bacterium *Staphylococcus aureus*. Infected host cells induce non‐specific immune responses upon detection of a pathogen. These include the upregulation of inducible nitric oxide synthase, an enzyme producing nitrogen radicals. Nitrogen radicals (NO·, nitric oxide) are toxic to cells by reacting with thiol groups and metalloproteins, both enriched in the respiratory chain (Bogdan, 2015). Pathogens relying on oxidative phosphorylation can therefore be semi‐specifically targeted by nitric oxide (Olive & Sassetti, 2016). Some strains of *Staphylococcus aureus* have evolved to resist nitric oxide and express the enzyme LDH1 (Richardson, Libby & Fang, 2008). LDH1 expression leads to an increase in lactate fermentation, thus ensuring energy production for the bacterium under both aerobic and anaerobic conditions. Nitric oxide‐resistant *Staphylococcus aureus* strains are therefore insensitive to the inhibition of the oxidative phosphorylation pathway by NO·, instead requiring glycolysis for ATP generation (Vitko, Spahich & Richardson, 2015).

Viruses rely on host cell metabolism to support their life cycle and propagation, and can interfere with the regulation of glycolysis by host cells. For example, hepatitis C virus and several members of the *Herpesviridae* family of DNA viruses deregulate glycolysis (Mayer *et al*., 2019; Goodwin, Xu & Munger, 2015). Viral strategies for enhancing glycolysis include upregulating glucose transporters to increase substrate availability (Yu, Maguire & Alwine, 2014); targeting the tightly regulated enzymes PFK‐1 (Abrantes *et al*., 2012; Munger *et al*., 2006) or HK (Ramière *et al*., 2014) to increase glycolytic flux; and inducing aerobic glycolysis *via* microRNAs (miRNAs) to support latency (Yogev *et al*., 2014). In some cases, metabolic changes induced by viral infections might have an indirect cause, for example, they could be triggered by changes in proliferation and protein biosynthesis. Nonetheless, the shifted metabolic state of infected cells is a potential target for the development of broadly active antiviral therapeutics. For example, while no vaccines or antiviral treatments are available for either Dengue virus or human norovirus despite the high global disease burdens they cause, glycolysis has been identified as a virulence factor for both viruses. Targeting glycolysis by specific metabolic inhibitors could therefore present a promising therapeutic avenue (Allonso *et al*., 2015; Fontaine *et al*., 2015; Passalacqua *et al*., 2019).

## VI. THE ROLES OF GLYCOLYSIS IN BIOTECHNOLOGY AND METABOLIC ENGINEERING

The structure of metabolism generates a series of constraints that determine, and often limit, the metabolic flux in the biochemical reaction network. For instance, fermentation converts pyruvate, the end product of glycolysis, to other metabolites in order to balance the NADH generated in the conversion of glucose to pyruvate. The two most frequently applied routes for NADH balancing are the conversion of pyruvate into lactic acid by LDH or into ethanol by two enzymes, pyruvate decarboxylase (PDC) and alcohol dehydrogenase. The latter route is used by yeast, and this has been exploited by humans for thousands of years. The first reports on human production of fermented beverages date to around 7000 BCE in China (Sicard & Legras, 2011), and there is also evidence for wine and beer production in Mesopotamia around 5000 BCE (Rasmussen, 2015). Over these millennia, there has been selection for yeast strains with a very efficient glycolytic pathway. This is particularly the case for *Saccharomyces cerevisiae*, which is used for production of bread, beer and wine. This yeast can carry a glycolytic flux on the order of 20 millimoles per gram dry biomass per hour, which is probably the highest glycolytic flux of any organism. This efficiency has evolved through optimisation of glycolytic enzymes and, more importantly, through optimising regulation such that there is a high degree of coordinated expression of these individual enzymes. The yeast has also adapted to allow substantial proteome allocation to glycolysis during rapid growth (Nielsen, 2019). Lactic acid bacteria, which are used for producing fermented milk products, also have a very high flux capacity. In these bacteria, pyruvate is converted to lactic acid *via* LDH. This highly conserved enzyme is also present in human cells, where it plays an important role under anoxic conditions in muscle cells and during rapid growth of cancer cells.

With its traditional applications in the production of fermented beverages, it was natural also to use *Saccharomyces cerevisiae* for production of ethanol for fuel (Nielsen, 2019). Today, this yeast produces about 110 billion litres of bioethanol used as a blend‐in fuel into gasoline. In this process, yeast is grown anaerobically with glucose (or sucrose) as the feedstock, and about 47% of the glucose mass is recovered as ethanol (>92% of the theoretical yield). The remainder is converted into biomass, CO_2_, and glycerol. While the yeast cells (biomass) and the CO_2_ can be recovered as co‐products, glycerol is an undesired byproduct formed due to a redox imbalance in cell growth, and there has therefore been much interest in reducing or eliminating its formation; e.g. by functional expression of Calvin cycle enzymes to use CO_2_ as electron acceptor for NADH reoxidation which decreases glycerol formation (Guadalupe‐Medina *et al*., 2013). During cell growth, some of the glucose is converted into biomass, and this process involves a net production of NADH and consumption of NADPH. As the conversion of glucose to ethanol is redox‐balanced, the only way for the cell to remove the accumulated NADH under anaerobic conditions is to convert some of the glucose to glycerol. However, through engineering of nitrogen assimilation (normally an NADPH‐consuming process) to consume NADH instead, it was possible to reduce glycerol production by about 50%, resulting in a 5% increase in ethanol production (Nissen *et al*., 2000). This illustrates the deep interconnections between glycolysis and many other metabolic pathways, which can present both opportunities and difficulties in cellular metabolism engineering (Nielsen & Keasling, 2016). In another approach to reduce glycerol production, the two glycolytic enzymes GAPDH and PGK were partly surpassed by expressing the non‐phosphorylating GAPN (Bro *et al*., 2006). The conversion of GAP to 3PG by this enzyme produces NADPH but does not form ATP, and hence it replaces production of NADH and ATP with NADPH. This allows a net consumption of NADH and production of NADPH when glucose is converted to ethanol, also reducing glycerol production by about 50% and increasing ethanol production by about 5% (Bro *et al*., 2006).

Considering the high flux capacity of glycolysis, there has also been much interest in recruiting this pathway for producing many other metabolites. Thus, microbial cell factories are widely used for production of food and feed ingredients, pharmaceuticals, chemical building blocks and biofuels. In many cases, these products are based on metabolites synthesised from precursor glycolytic intermediates, and it therefore becomes worthwhile to redirect flux towards this precursor to ensure efficient production of the desired product. One example is acetyl‐CoA, which is a precursor for synthesis of fatty acids and isoprenoids. Acetyl‐CoA can be derived from pyruvate by several different routes, including a direct, non‐oxidative route through the enzyme PKT to synthesise acetyl‐CoA from phosphorylated sugars. PKTs can convert either (*i*) Xu5P to GAP and Ace‐P or (*ii*) G6P to E4P and Ace‐P (Bergman *et al*., 2016). This Ace‐P can then be converted directly to acetyl‐CoA by a phosphate acetyltransferase enzyme. PKTs are present in relatively few bacteria, but they can be heterologously expressed in other microorganisms. This has been exploited to establish a NOG pathway by combining the expression of F6P‐targeting PKTs together with enzymes of the PPP (Bogorad *et al*., 2013). The NOG pathway enables conversion of 1 mole of F6P to 3 moles of acetyl‐CoA without any consumption or formation of ATP and NADH, and it thus provides a high‐yielding acetyl‐CoA pathway that can be exploited to produce acetyl‐CoA‐derived products. A similar strategy was exploited for production of the sesquiterpene farnesene (Meadows *et al*., 2016), which is used as a blend in biodiesel. Here, PKT expression was combined with many other genetic modifications to rewire the glycolytic flux to obtain a high yield of the acetyl‐CoA‐derived product.

The high‐capacity glycolytic pathway of *Saccharomyces cerevisiae* has evolved to convert sugars rapidly into ethanol. This is an inherent trait of *Saccharomyces cerevisiae*, and as discussed above, this route is used even in the presence of oxygen and high glucose concentration (the Crabtree effect, see Section V.1.*a*) (Dai *et al*., 2018). However, ethanol production is undesirable in many biotechnological applications; this has spurred efforts to decouple glycolytic flux from ethanol production, as this pathway can then be used to feed precursors for other useful products. Ethanol production can be abolished in *Saccharomyces cerevisiae* by deleting all three genes encoding PDCs (Dai *et al*., 2018; Flikweert *et al*, 1996). However, this has a detrimental effect on growth and requires establishment of an alternative route to the acetyl‐CoA required for lipid biosynthesis. An example for this situation is a study in which pyruvate oxidase was expressed together with deletion of the PDCs (Dai *et al*., 2018). Pyruvate oxidase converts pyruvate to acetate, which can be activated to acetyl‐CoA by acetyl‐CoA synthase. Even with several other genetic mutations, growth of the engineered organism was very slow. However, through adaptive laboratory evolution (ALE), it was possible to isolate mutants with a higher growth rate following whole‐genome sequencing. The faster‐growing mutants had a mutation in a key component of the Mediator complex (Dai *et al*., 2018). This complex is involved in transcriptional regulation, and the mutation causes global transcriptional reprogramming that downregulated glycolytic genes and sugar transporters and upregulated genes coding for enzymes involved in pyruvate oxidation (e.g. the citric acid cycle and respiration) (Dai *et al*., 2018). This mutation established a new balanced level of all the glycolytic enzymes adjusted to a lower flux level, enabling the cells to grow faster. Similarly, the use of pyruvate for fatty acid production was evaluated to see if this route could replace ethanol production. Yeast can be engineered to produce high levels of free fatty acids (Zhou *et al*., 2016), but the cells still produce ethanol as the dominant product. However, through ALE following deletion of the PDCs, it was possible to eliminate ethanol production and still obtain high growth rates (Yu *et al*., 2018). The ALE‐derived strains displayed mutations in PK that attenuated its activity; therefore, in this case, it was necessary to adjust the glycolytic flux to a level allowing pyruvate to be drained towards fatty acid biosynthesis (Yu *et al*., 2018). The resulting strain could produce very high levels of free fatty acids with applications as food ingredients, lubricants and biofuels.

These examples illustrate the difficulty of engineering the glycolytic pathway in a directed fashion. In early studies, it was not possible to increase glycolytic flux by overexpressing the individual glycolytic enzymes (Schaaff *et al*., 1989) or groups of enzymes (Peter Smits *et al*., 2000). Based on mathematical modelling of glycolysis, we now know that this is due to an almost equal distribution of flux control over all the enzymes in the pathway (Nilsson & Nielsen, 2016) – i.e. it would only be possible to increase glycolytic flux if *all* enzymes are overexpressed. This is not possible, however, as the glycolytic enzymes already account for a large fraction of the cellular proteome; complete overexpression would therefore require a major proteome reallocation from other key cellular processes, impacting cell growth and indirectly affecting glycolytic flux. However, even at fast growth, the glycolytic pathway does seem to have some extra capacity, as it is possible to increase flux by installing a pull on ATP. This was illustrated by expressing a non‐proton‐pumping ATPase in *Escherichia coli* (Koebmann *et al*., 2002a), but the above example of GAPN expression in yeast also indicates this. Overall, these studies show that due to the very extensive regulation of glycolysis, which is important for controlling its many different functions, it is difficult to modulate flux through this pathway. With advancements in synthetic biology it may, however, be possible to gain new insights into the many different levels of regulation and to use this information for bioengineering. One excellent example is expression of all the yeast glycolytic genes on a single artificial chromosome in cells where the endogenous genes were deleted (Kuijpers *et al*., 2016). With this system, it is possible to control the altered expression of individual genes better and to study how the pathway can operate in different modes.

## VII. CONCLUSIONS

(1) Glycolysis stands as a pivotal metabolic pathway whose understanding has profound implications for both basic and applied sciences. In this review we have charted the course of glycolytic research, from its evolutionary origins and biochemical nuances to its multifaceted roles in health, disease, and biotechnology.
(2) The integration of advanced analytical techniques and modelling approaches has revolutionised our ability to study glycolytic activity and regulation with unprecedented precision. As such, glycolysis remains a blueprint for advancing biochemistry and cell biology.
(3) As we continue to uncover the complexities of glycolysis and its interconnections with other metabolic processes, the insights gained will not only enhance our comprehension of cellular metabolism but also pave the way for innovative therapeutic and biotechnological applications.
(4) The enduring relevance of glycolysis underscores its central place in the study of life's biochemical foundations and its potential to drive future scientific breakthroughs.
